# Patterns of variation in cis-regulatory regions: examining evidence of purifying selection

**DOI:** 10.1186/s12864-017-4422-y

**Published:** 2018-01-26

**Authors:** Thijessen Naidoo, Per Sjödin, Carina Schlebusch, Mattias Jakobsson

**Affiliations:** 1 Department of Organismal Biology, Uppsala University, Uppsala, Sweden; 2 Science for Life Lab, Uppsala, Sweden

**Keywords:** Regulatory regions, Purifying selection, Selection efficacy, Non-coding DNA, Functional elements, Population genetics

## Abstract

**Background:**

With only 2 % of the human genome consisting of protein coding genes, functionality across the rest of the genome has been the subject of much debate. This has gained further impetus in recent years due to a rapidly growing catalogue of genomic elements, based primarily on biochemical signatures (e.g. the ENCODE project). While the assessment of functionality is a complex task, the presence of selection acting on a genomic region is a strong indicator of importance. In this study, we apply population genetic methods to investigate signals overlaying several classes of regulatory elements.

**Results:**

We disentangle signals of purifying selection acting directly on regulatory elements from the confounding factors of demography and purifying selection linked to e.g. nearby protein coding regions. We confirm the importance of regulatory regions proximal to coding sequence, while also finding differential levels of selection at distal regions. We note differences in purifying selection among transcription factor families. Signals of constraint at some genomic classes were also strongly dependent on their physical location relative to coding sequence. In addition, levels of selection efficacy across genomic classes differed between African and non-African populations.

**Conclusions:**

In order to assign a valid signal of selection to a particular class of genomic sequence, we show that it is crucial to isolate the signal by accounting for the effects of demography and linked-purifying selection. Our study highlights the intricate interplay of factors affecting signals of selection on functional elements.

**Electronic supplementary material:**

The online version of this article (10.1186/s12864-017-4422-y) contains supplementary material, which is available to authorized users.

## Background

The mammoth task of identifying functional elements in the human genome began decades before the genomic era and still continues today. Much of the pre-genomic efforts were focused on the discovery and functional characterization of protein coding genes, using linkage to identify their locations, and experimental approaches, often employing sequence disruption to evaluate functionality [1]. While thousands of protein coding genes had already been discovered prior to the release of the draft sequence of the human genome [2, 3], this landmark event represented a drastic acceleration in the identification of both protein coding genes and non-coding elements, and provided a launchpad for further plans to identify additional elements. The search for non-coding functional elements started early on [4–6], but the release of the human genome sequence provided much-needed impetus to evaluate methods and technologies available for the identification of functional elements. This effort culminated in the form of the ENCODE Project Consortium, which undertook a comprehensive annotation of functional elements in the human genome. The rapid advances in DNA sequencing technology and genomic assays in the past decade allowed for the release of the aforementioned annotation in 2012 [7]. ENCODE utilised a primarily biochemical approach to map functional elements; using such signatures as methylation, DNase sensitivity and transcription factor occupancy to determine regions in the genome displaying potential functionality. These biochemical signatures, while indicative of activity at a site or region, can also occur stochastically [8], and so, cannot be regarded as indisputable evidence of functionality [9]. With this in mind, the database of elements provided by ENCODE still contain a promising list of candidates to be evaluated for functionality.

Comparative genomics approaches have also been used to identify functional elements, through the search for patterns of conservation in multi-species sequence alignments [10]. Conserved sequences exist due to a lowered substitution rate; caused by the removal of deleterious mutations from regions subject to purifying selection by virtue of their biological importance. These methods have also been used to provide an estimate for the proportion of functional sites in mammalian genomes [10]; estimated at ~5%. This estimate is drastically different to estimates from the ENCODE Project, which range between 20% and 80%, based on biochemical signatures [7]. Since the publication of the ENCODE results, these estimates have been disputed, often due to the discrepancy with estimates based on evolutionary constraint and definitions of functionality [11]. While biochemical signatures cannot be held up as sole evidence of function, comparative genomic estimates of function are also not without caveat. A majority of highly conserved regions detected by comparative genomics investigations have yet to be verified experimentally, through biochemical or functional assays [12]. This need is illustrated by the ultra-conserved elements [13], which are genomic regions longer than 200 bases that maintain 100% identity in human, rat and mouse genomes. While some of these regions have been assigned functionality – e.g. transcriptional enhancers [14] – most are still functionally a mystery [15]. Since the release of the ENCODE data, additional conserved regions have been assigned functions; however, this is mainly due to the expansion of annotated genomic space [12]. Still, this provides support for an integrated approach, which incorporates multiple strategies. Another major drawback of comparative genomics is its inability to detect lineage-specific constraint [9, 16]. These methods are better suited for detecting functional regions that have been under selective pressure for very long periods of evolutionary time, in contrast to detecting functional regions affected by recent selective pressure [17] and high rates of turnover [9, 18]. Population genetic methods have been used to address the former case, in examining regions of the genome under recent selection. Ward and Kellis [16] estimated that a further 4% of the genome was under lineage-specific constraint in humans. ENCODE-annotated elements have also been implicated as showing signals of purifying selection [19, 20]. The patterns of variation uncovered by population genetic methods, however, are affected both by selection and by demographic factors [21]. The use of a selection-neutral reference can be used to control for the effects of demography [21, 22]. Additional confounding factors such as linked-purifying (or background) selection [23, 24] may also increase the difficulty of elucidating valid signals of selection on elements in the genome. Indeed, Hernandez et al. [25] found selection signals in conserved non-coding regions and noted that the proximity of these regions to exons may have been responsible for these observations.

In this study we undertook a comprehensive analysis of patterns of variation in regulatory elements of the human genome among a diverse dataset of populations from across the world, with multiple sub-Saharan Africans, including Khoe-San populations that capture the deepest split among humans (>100 kya) compared to other African and non-African groups [26]. In addition, we relied on a selection-neutral genomic reference and the spatial organisation of elements to control for the effects of demography and linked-purifying selection, respectively. Our results indicate differing selective pressures across regulatory elements; depending as well on proximity to coding sequence.

## Results

### Signs of demography

**Fig. 1:**
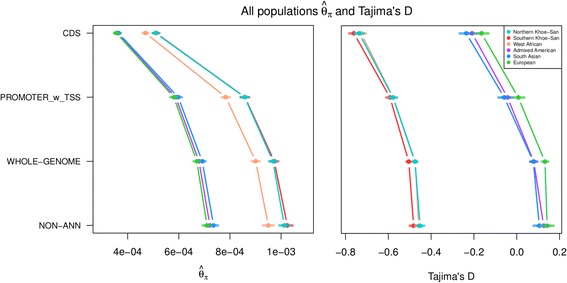
Estimated means and 95% confidence intervals of *θ*_*π*_ and Tajima’s D in the six global pools for non-annotated regions (NON-ANN), protein coding sequence (CDS), predicted promoter regions (PROMOTER_w_TSS), and the whole genome, denoting the clear delineation between African and Non-African populations

When examining the results for the non-annotated class, we observed the well-known reduced diversity in non-Africans, which has been attributed to the Out-of-Africa bottleneck [27]. The African populations (*θ*_*π*_NKS = 0.00101, *θ*_*π*_SKS = 0.00102, and *θ*_*π*_WAF = 0.00095) all exhibited higher levels of diversity in comparison to the non-African populations (*θ*_*π*_AMR = 0.00072, *θ*_*π*_SAS = 0.00074, and *θ*_*π*_EUR = 0.00071) (Fig. 1 and Additional file 1: Figure S1; Table 1 for details of populations). Tajima’s D for the non-annotated class appeared to be consistent with expectations from the general features of the populations’ demographic histories, with a negative Tajima’s D in African populations (D_NKS_ = −0.451, D_SKS_ = −0.482, and D_WAF_ = −0.458) reflecting population expansion, and a positive Tajima’s D in non-Africans (D_AMR_ = 0.126, D_SAS_ = 0.105, and D_EUR_ = 0.149) (Fig. 1 and Additional file 1: Figure S2) reflecting a strong bottleneck that overshadows a recent expansion [28]. Due to the apparent delineation between African (AFR) and non-African (N-AFR) populations, the combined averages for the two broad groupings were used primarily, with population-specific results shown where necessary.

**Table 1:** Description of sample groups

Global Pool (code)	Population (code)	Country	Sample size
Northern Khoe-San (NKS)	Ju/‘hoansi (JUH)	Namibia	5
Northern Khoe-San (NKS)	!Xun (XUN)	Angola	4
Southern Khoe-San (SKS)	Karretjie (KAR)	South Africa	4
Southern Khoe-San (SKS)	Nama (NAM)	Namibia	5
West African origin (WAF)	Luhya (LWK)	Kenya	5
West African origin (WAF)	Yoruba (YRI)	Nigeria	4
admixed Indigenous American (AMR)	Mexican ancestry (MXL)	USA	5
admixed Indigenous American (AMR)	Peruvian (PEL)	Peru	4
European origin (EUR)	Italian (TSI)	Italy	4
European origin (EUR)	Northern & Western European ancestry (CEU)	USA	5
South Asian (SAS)	Gujarati ancestry (GIH)	USA	4
South Asian (SAS)	Punjabi (PJL)	Pakistan	5

### Overall levels of diversity and selection in regulatory regions

**Fig. 2:**
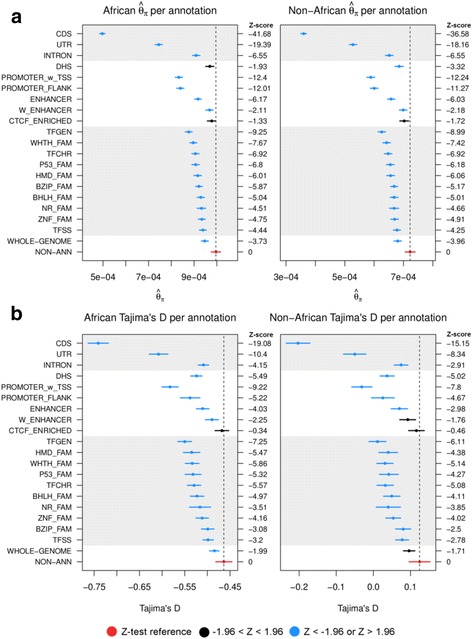
Estimated means and 95% confidence intervals of **a** *θ*_*π*_ and **b** Tajima’s D in Africans and Non-Africans for genomic classes across the genome. The threshold for significant difference to non-annotated sequence was set at *p* < 0.05 (Z < −1.96 or Z > 1.96). Details of the classes found in Table 2

For protein coding genes, the coding sequence (CDS) was, invariably, the most conserved (least diverse) category in the genome by far (*θ*_*π*_AFR = 0.00050, *θ*_*π*_N-AFR = 0.00036), followed by the untranslated regions (UTR) (*θ*_*π*_AFR = 0.00074, *θ*_*π*_N-AFR = 0.00053). Intronic sequence also showed a noticeable decrease in diversity (*θ*_*π*_AFR = 0.00091, *θ*_*π*_N-AFR = 0.00065, Fig. 2a, Table 2).

**Table 2:** Genomic classes used in the study

Category Code	Category Description
CDS	Protein coding sequence regions
UTR	Protein coding untranslated regions
INTRON	Protein coding intronic regions
DHS	DNase hypersensitive regions
Genome Segmentations	
PROMOTER_w_TSS	Predicted promoter region including transcription start site
PROMOTER_FLANK	Predicted promoter flanking region
ENHANCER	Predicted enhancer
W_ENHANCER	Predicted weak enhancer
CTCF_ENRICHED	CTCF enriched elements
Transcription Factor Binding Sites	
TFGEN	General transcription factors
TFCHR	Chromatin-modifying transcription factors
TFSS	Sequence-specific transcription factors
BHLH_FAM	Basic helix-loop-helix protein family
BZIP_FAM	Basic leucine zipper family
HMD_FAM	Homeobox-domain family
NR_FAM	Nuclear hormone receptor family
P53_FAM	P53-like transcription factor family
WHTH_FAM	Winged helix-turn-helix family
ZNF_FAM	Zinc finger protein family
Reference Regions	
NON_ANN	Non-annotated regions at least 200 kb away from CDS
WHOLE_GENOME	Whole genome average

The genome segmentation classes exhibited varying patterns of variation. Promoters showed several signs of being under purifying selection, with the lowest levels of diversity compared to the non-annotated class (*θ*_*π*_AFR = 0.00083, *θ*_*π*_N-AFR = 0.00059), and the lowest values of Tajima’s D (D_AFR_ = −0.58245, D_N-AFR_ = −0.03135). Promoter flanking regions also showed similarly low levels of diversity (*θ*_*π*_AFR = 0.00084, *θ*_*π*_N-AFR = 0.00060), but their Tajima’s D estimates, while still significant, were not as low (D_AFR_ = −0.53822, D_N-AFR_ = 0.02555). Enhancers were slightly lower than the whole genome average for both diversity (*θ*_*π*_AFR = 0.00092, *θ*_*π*_N-AFR = 0.00066) and Tajima’s D (D_AFR_ = −0.51041, D_N-AFR_ = 0.06994); quite similar to the intron class. Weak enhancers were significantly less diverse compared to the non-annotated class, but only just (*θ*_*π*_AFR = 0.00097, *θ*_*π*_N-AFR = 0.00070); while only having significantly lower estimates of Tajima’s D in African populations (D_AFR_ = −0.49004, D_N-AFR_ = 0.09227). CTCF enriched regions were not significantly different from non-annotated sequence for either diversity (*θ*_*π*_AFR = 0.00098, *θ*_*π*_N-AFR = 0.00070) or Tajima’s D (D_AFR_ = −0.46795, D_N-AFR_ = 0.11596) (Fig. 2a and b; Z-scores provided in the figures).

The transcription factor (TF) classes, including sequence-specific families, also showed significantly lower levels of diversity than non-annotated sequence; though it was more difficult to discern differences among them than for the genome segmentation classes. General TFs, however, showed signs of being under stronger purifying selection with the lowest diversity estimates (*θ*_*π*_AFR = 0.00088, *θ*_*π*_N-AFR = 0.00063) and lowest Tajima’s D estimates (D_AFR_ = −0.550277, D_N-AFR_ = 0.011069) among TF classes. The collective sequence-specific TF and the BZIP classes were at the other end of the spectrum and only slightly lower than the whole genome average in diversity estimates and Tajima’s D (Fig. 2a and b). Using overall estimates, we could not adequately separate the TF classes for intensity of purifying selection, although we discuss later how we improved the resolution among some of these TF classes.

While the results for genome segmentations and TFs were relatively consistent across summary statistics, some elements displayed conflicting results; such as DNase hypersensitive regions (DHS), which have long been used as a proxy for active chromatin due to the binding of regulatory factors [29, 30]. While Tajima’s D estimates for DHS (D_AFR_ = −0.52428, D_N-AFR_ = 0.03731) showed significant differences to non-annotated sequence, only the non-African diversity estimate (*θ*_*π*_AFR = 0.00097, *θ*_*π*_N-AFR = 0.00069) was significantly different (Fig. 2a and b).

The neutrality index (NI), based on a McDonald–Kreitman (MK) test, was also calculated as an additional measure of selection. While the NI found varying levels of purifying selection across the investigated elements (2.5% – 15%), it was unable to discern significant differences between them; apart from the CTCF enriched regions, which were noticeably lower (2.5%) than the other classes (7.9% - 15%) (Additional file 1: Figure S3).

### Influence of linked-purifying selection

**Fig. 3:**
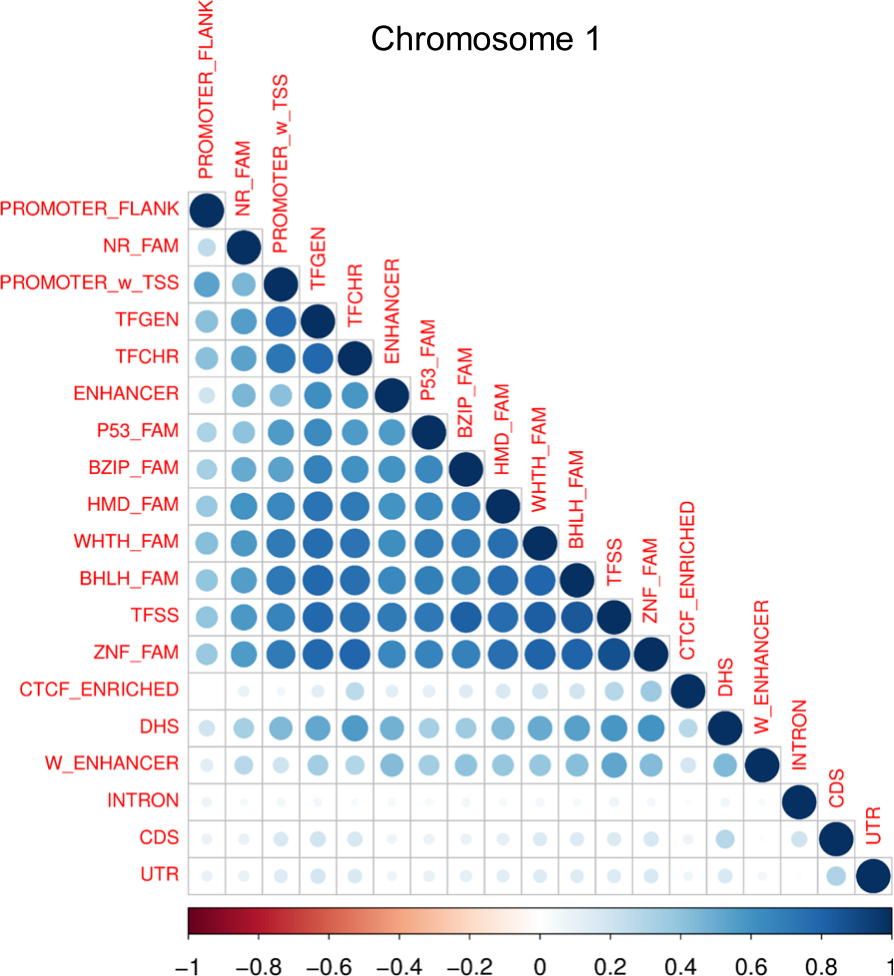
Pairwise correlations (Pearson’s r) for genomic classes on chromosome 1

We determined if the signal noted for the various non-coding elements was caused by selection acting on those particular regions, as opposed to a selection signal caused by linkage to coding regions known to be under purifying selection, i.e. linked-purifying selection. In order to separate these two factors, we examined whether the relative locations of the elements influenced the estimates of the summary statistics used in the search for selection acting on these elements. We sought to roughly ascertain how positionally associated the elements were by performing a pairwise sliding-window correlation analysis [31] across chromosome 1 (Fig. 3). The results obtained for chromosome 1 were corroborated by the analysis of an additional chromosome (chromosome 10; Additional file 1: Figure S4), as both chromosomes showed similar pairwise correlations among the elements.

The TF classes were highly correlated (measured with Pearson’s r), with general, chromatin-modifying and sequence-specific binding regions overlapping with each other substantially. The TF classes also correlated highly with the promoters and enhancers, while showing relatively lower correlations with the promoter flanking regions. Weak enhancers only showed moderate levels of correlation to TF classes and other genome segmentations. The CDS, however, showed weak positional association with TF classes and genome segmentations, with low levels of correlation (chromosome 1: 0.0194 to 0.1962; chromosome 10: 0.0022 to 0.1627). The CDS did show slightly greater correlation with UTR (chromosome 1: 0.3018; chromosome 10: 0.3019), introns (chromosome 1: 0.2024; chromosome 10: 0.2115), and DHS regions (chromosome 1: 0.2825; chromosome 10: 0.2228).

Next, we assessed if that level of correlation in combination with strong purifying selection on CDS was enough to influence the results of the other elements via linked-purifying selection. We investigated if the weak positional associations to CDS were correlated with the estimates of *θ*_*π*_ and Tajima’s D of the other genomic classes and found moderate negative correlation per population (*θ*_*π*_: −0.4054 to −0.4877, Tajima’s D: −0.5211 to −0.6514; Additional file 1: Table S3). We also checked if the total sizes (in bases) of the genomic classes were correlated with the above summary statistics and found only weak positive correlation for Tajima’s D (0.1465 to 0.2459) and almost no correlation for *θ*_*π*_ (0.0603 to 0.0679; Additional file 1: Table S3). These findings indicated that, while the sizes of the genomic classes had potentially little to no effect, linked-purifying selection stemming from CDS likely influenced the results of other genomic classes. In order to quantify this effect, we studied how linked-purifying selection affected potentially neutral sequence in close proximity to CDS (the most intense signal of purifying selection). *θ*_*π*_ and Tajima’s D were computed for discrete bins of non-annotated sequence, at increasing distance from CDS, in order to isolate the linked-purifying selection signal (Additional file 1: Figures S5 and S6). In all populations, *θ*_*π*_ exhibited a consistent pattern, with significant reductions in diversity up to at least 7.5 kb away from CDS (with non-African populations extending till 10 kb). The effect of linked-purifying selection on Tajima’s D, however, was not as clear. Nevertheless, it was still possible to utilise both of these results, when we disentangled the effects of purifying and linked-purifying selection.

**Fig. 4:**
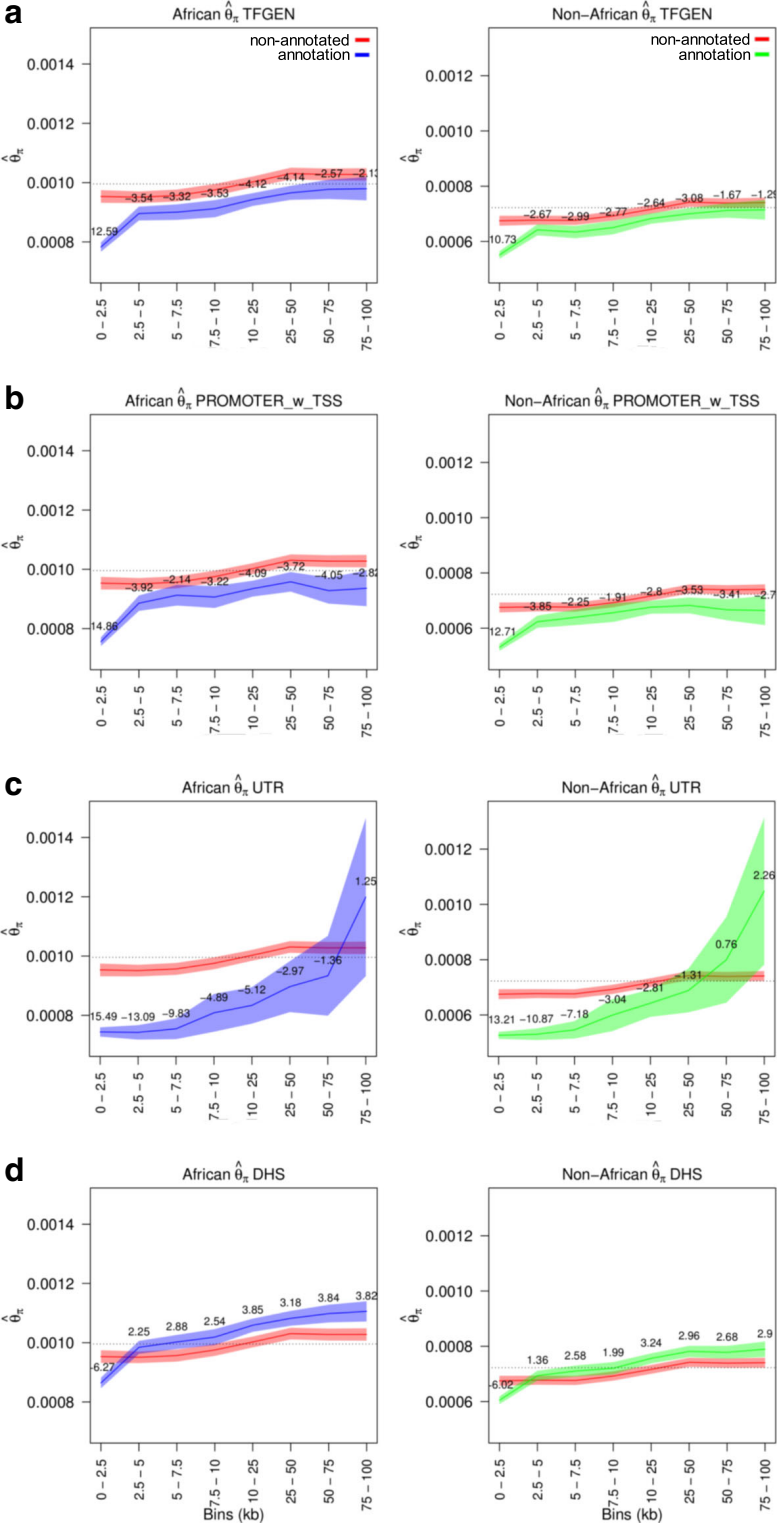
*θ*_*π*_ at varying distance from CDS for non-annotated sequence (red) versus **a** general TFs, **b** Promoters, **c** UTR, and **d** DHS, in African (blue) and non-African (green) populations. Neutral reference (non-annotated sequence at least 200 kb away from CDS) is illustrated by the dotted line. Shaded areas represent 95% confidence intervals, with Z-scores (non-annotated vs. annotation) shown per bin

**Fig. 5:**
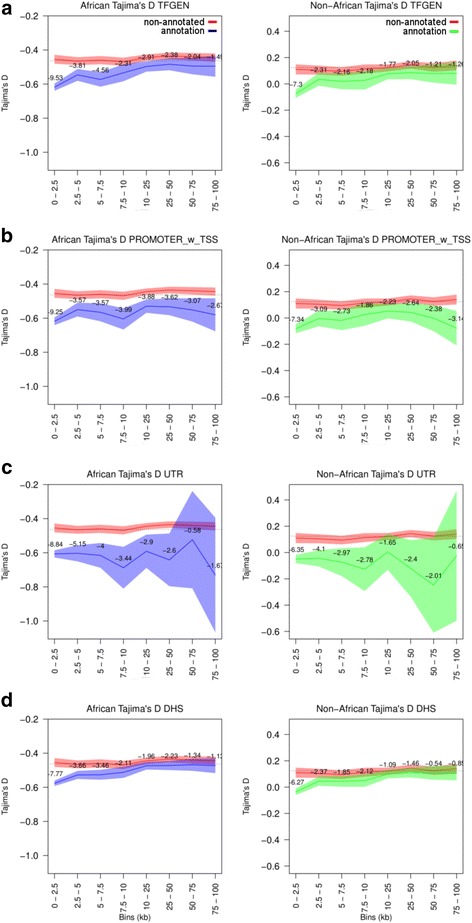
Tajima’s D at varying distance from CDS for non-annotated sequence (red) versus **a** general TFs, **b** Promoters, **c** UTR, and **d** DHS regions, in African (blue) and non-African (green) populations. Neutral reference (non-annotated sequence at least 200 kb away from CDS) is illustrated by the dotted line. Shaded areas represent 95% confidence intervals, with Z-scores (non-annotated vs. annotation) shown per bin

Through demonstrating the effect of linked-purifying selection on non-annotated regions in the vicinity of genomic elements under strong purifying selection (i.e. CDS), we were able to unhitch this effect from other genomic elements in the same vicinity, in a quantifiable manner. We computed *θ*_*π*_ (Fig. 4 and Additional file 1: Figure S7) and Tajima’s D (Fig. 5 and Additional file 1: Figure S8) within discrete bins at increasing distance from CDS for each of the elements under investigation. These distributions (of *θ*_*π*_ and Tajima’s D) were then compared to distributions (of *θ*_*π*_ and Tajima’s D) for non-annotated sequence within each discrete bin, using a Z-test. For both *θ*_*π*_ and Tajima’s D, the distributions for most elements (Figs. 4, 5, and Additional file 1: Figures S7, S8) differed significantly from non-annotated sequence, with stronger signals of selection especially within 2.5 kb of CDS. We interpreted this higher intensity, relative to non-annotated sequence at the same distance from CDS, as purifying selection independent of the linked-purifying selection from CDS. From these results, we observed strong disparities in selection strength between proximal (within 2.5 kb of CDS) and distal regions; and also among the genomic classes included in the study.

### Efficacy of selection

Comparisons of regulatory elements to non-annotated sequence were used to reduce the effects of demography on the site frequency spectrum (SFS) summary statistics. We were, however, unable to remove all effects; notably, the signals were still affected by efficacy of selection. This observation was indicated by noticeably lower signals of selection in non-African populations. In terms of purifying selection, while the ability of selection to remove deleterious alleles from a population is dependent on the selection coefficient, the product of the selection coefficient and the effective population size (Ne) is determinative of its efficacy [32]. The difference between African and non-African populations was not as noticeable in the overall estimates of *θ*_*π*_ (Fig. 2a), but there was some indication of it in the Tajima’s D estimates (Fig. 2b). The effect became more pronounced in both *θ*_*π*_ and Tajima’s D, however, when displayed as a function of distance from CDS (Figs. 4, 5, and Additional file 1: Figures S7 to S44). There has been considerable debate on whether the efficacy of selection has varied across human populations [33–36]. Our results support a lowered efficacy of selection in non-African populations. These results held across all populations, with very consistent spatial patterns of diversity (Additional file 1: Figures S9 to S44).

## Discussion

We searched for signals of selection primarily among predicted regulatory elements and regions bound by TFs, and used two different approaches in order to separate the demographic and selection signals: (i) by comparing results from different population groups, known to have experienced some differences in their more recent demographic histories; and (ii) through the use of a “selection-neutral” reference, comprised of non-annotated regions of the genome, which allowed us to control for the effects of demography on each population group. In using non-annotated sequence as a neutral reference, we assumed that these regions were less constrained, evolutionarily. This assumption appeared justified, as non-annotated sequence contained the most diversity overall when screened, which supported our use of the non-annotated class as a proxy for neutral genomic regions.

Our overall estimates were corroborated by the results of previous studies [16, 19, 20], where regulatory elements were shown to be under more constraint than a neutral reference [19], a bootstrap-generated distribution from a specified background [16] or the genome average [20]. The observations of reduced diversity in specific regions, while consistent with differential levels of purifying selection across the various investigated elements, were also consistent with differential levels of linked-purifying selection. Linked-purifying selection is usually invoked to describe the decrease in genetic diversity of a non-deleterious region of DNA due to purifying selection acting on a linked region [23, 24]. Together with genetic hitchhiking [37], linked-purifying selection is a phenomenon where the consequences of selection at a particular site can alter the population genetic dynamics and the patterns of genetic variation of genetically linked neutral (and non-neutral) sites that compose its genetic background [38]. Unlike in genetic hitchhiking, where the frequency of a neutral allele is altered due to its close proximity to a region undergoing a selective sweep, linked-purifying selection often purges neutral alleles from a population due to their close proximity to deleterious mutations.

Despite the weak positional association of CDS to the TF classes and genome segmentations, the level of association was high enough to influence the results of the other genomic classes, highlighting the effect of linked-purifying selection. In order to quantify this effect, we used the neutral non-annotated sequence near CDS. We demonstrated that this non-annotated sequence, unlike our selection-neutral reference, was affected by its proximity to regions under strong purifying selection; thus allowing us to unhitch this effect from other elements in the same vicinity. Ward and Kellis [16] also showed a consistent reduction in heterozygosity as genetic distance to exons was decreased (Fig. 1c in [16]) when comparing ENCODE-annotated to non-annotated elements. Their signal was constant till roughly 0.01 cM, which on average is comparable to the estimates we found.

### Proximal versus distal regions

We observed strong disparities in selection strength between proximal (within 2.5 kb) and distal regions for most genomic classes. This highlighted the importance of elements found in close proximity to CDS (e.g. general TFs [Figs. 4a and 5a]). Previously, some TFs were shown to exhibit much stronger signals of selection at proximal sites versus distal sites [19, 20]. Linked-purifying selection extending from CDS was proposed as one of the possible reasons for this pattern [19]. Hernandez et al. [25] also invoked linked-purifying selection as one of the main reasons for a trough in diversity extending from exonic regions. Our results show that while linked-purifying selection significantly reduces diversity surrounding CDS (as evidenced by the effect on potentially neutral non-annotated sequence), this reduction comprises only a portion of the effect, with regulatory elements exhibiting further reduced levels, especially within 2.5 kb of CDS.

While proximal regions provided the strongest signals, distal regions (tested up till 100 kb) also showed signals of purifying selection. We were able to show a strong spatial component, with some elements displaying moderate levels of purifying selection up to almost 100 kb from CDS (e.g. promoter regions [Figs. 4b and 5b]), while the signal for others (e.g. CTCF enriched regions [Additional file 1: Figures S7D and S8D]) collapsed immediately outside 2.5 kb of CDS. While these spatial patterns tended to reflect overall estimates of diversity and Tajima’s D (Fig. 2), they further allowed us to uncover differences among TF classes that were difficult to differentiate when looking at overall estimates. This was more apparent for diversity estimates than for Tajima’s D, which appeared to be less sensitive at these sample sizes. The comparison of chromatin-modifying TFs to the P53 family is a good illustration of this (Additional file 1: Figure S7 K and P). While chromatin-modifying TFs displayed a much greater decrease in diversity than P53 TFs within 2.5 kb of CDS, this signal extended only till around 10 kb. In contrast, moderate signals of purifying selection associated with P53 TFs extended till around 50-75 kb.

### Non-coding genic regions

The UTR class, collectively comprised of the 5’-UTR and 3’-UTR elements which play major roles in post-transcriptional (3’-UTR) and translational regulation (5’-UTR and 3’-UTR) [39], showed very strong signals of purifying selection. Notably, the signal was roughly consistent up to 7.5 kb (Figs. 4c and 5c). With close to 95% of UTR sites falling within 7.5 kb of CDS (around 80% within 2.5 kb), the pattern we observed points to strong purifying selection across much of the length of UTR elements. While we did not measure estimates for the 5’-UTR and 3’-UTR regions separately, Mu et al. [19] noted a slightly lower diversity for 3’-UTR elements. Introns also displayed moderate levels of purifying selection (Additional file 1: Figures S7B and S8B), especially close to CDS. While this signal decreased with increasing distance from CDS, low levels were still maintained till at least 50 kb into introns. Some degree of constraint is expected close to exons due to the presence of splice sites and the extent of selection presence may be due to the other functions that introns play, including gene regulation [40, 41].

### Support for genome segmentation accuracy

The genome segmentations used in this study were generated by integrating [42] two unsupervised chromatin state annotation algorithms, ChromHMM [43] and Segway [44]. The use of unsupervised methods allowed for enhanced discovery of potential functionality in the genome, with less bias toward well characterized regions. With distinct signals of purifying selection at most of these predicted annotations, as well as notable differences in the strength of selection among them, our results provide additional support for the utility of these methods for uncovering functional elements in the genome. Both promoters and promoter-flanking regions exhibited more constraint both proximally and distally than enhancers. Notably, the spatial distribution of purifying selection on promoters provided some indication of the importance of distal promoter elements (Figs. 4b and 5b). Enhancers (Additional file 1: Figures S7E and S8E), as well, were under stronger selection than weak enhancers (Additional file 1: Figures S7H and S8H). Overall estimates of diversity and Tajima’s D at CTCF enriched regions were not much different from the neutral reference (Fig. 2); a finding supported by similar results in [16]. This was surprising, since CTCF has several roles, acting in transcriptional activation and repression, as an insulator, and in chromatin structure [45]; and has previously been found in conserved regions [46, 47]. Once positionally de-constructed, however, purifying selection was detected at proximal CTCF sites (Additional file 1: Figures S7D and S8D). This potentially reconciles the prior contradictory findings; indicating that the multi-tasking CTCF may be evolutionarily relevant primarily when binding close to protein coding regions.

### Increased mutation rate in DHS?

While overall diversity and Tajima’s D estimates were contradictory, primarily for African populations, the comparison of DHS results proximal and distal to CDS provides some insight into this apparent contradiction. Proximal DHS elements were moderately constrained, but distal elements were significantly more diverse than non-annotated sequence regions (Fig. 4d). Tajima’s D estimates, however, showed purifying selection acting on DHS elements (Fig. 5d) till around 50 kb away (in African populations). This discrepancy may be due to an increased mutation rate at DHS sites. The ENCODE Project [7] found that DHS elements showed reduced diversity in humans and Thurman et al. [30] noted that this diversity was linked to the mutation rate of the cell lines from which the DHS elements were derived. When normalised for mutation rate, it was shown that DHS elements from only a few cell lines displayed reduced diversity. Sabarinathan et al. [48] was able to show substantially increased mutation rate in DHS elements, particularly those bound by TFs, due to reduced levels of nucleotide excision repair. An increase in mutation rate, under the influence of recombination and selection would result in an increase of *θ*_*w*_ over *θ*_*π*_ [49], thus resulting in a negative value for Tajima’s D. It should be noted, however, that the findings in Sabarinathan et al. [48] were based on somatic mutation rates, and so it is not clear how far this observation extends to germline mutation rates. In addition the increased mutation rate appeared to be a consequence of reduced levels of nucleotide excision repair in regions bound by TFs, suggesting an increased mutation rate at TFs themselves, yet we did not find this effect in any of the TF classes. This may be explained by the presence of purifying selection masking the effect of increased mutation rate.

## Conclusions

In searching for signals of selection among regulatory elements of the human genome, we demonstrated the importance of isolating the signal by accounting for the effects of demography and linked-purifying selection. The signals we uncovered were strongest from elements in close proximity to CDS, even after accounting for the impact of linked-purifying selection. Apart from these strong signals of purifying selection in regulatory elements proximal to CDS, we found variable distributions of selection in distal regions. By conditioning on distance to CDS, it became possible to discern differences in selection among some TF classes, using small sample sizes. Previously Khurana et al. [20] showed that when taking overall estimates into account, very large sample sizes were necessary to pick up clear differences between TF classes. It is, however, expected that high levels of positional correlation between TFs from different families would increase difficulty in discerning differences in selection (Fig. 3). The ability to find purifying selection acting on annotations based on biochemical signatures generated by the ENCODE project [7] again validated their efforts, and increased support for integrated approaches to identifying functionality in the genome. At the same time, it became clear that the presence of an annotated element was not always associated with a signal of purifying selection. The differences in selection efficacy uncovered between African and non-African populations, and the difficulty in removing that demographic signal from our data, is a consequence of the intricate relationship between selection and demography, where the ability of selection to remove deleterious alleles from a population is dependent on the demographic history of that population.

## Methods

### Genome sequence data

The SNP data (limited to autosomes only) used in the study were sourced and extracted from samples in the Complete Genomics diversity panel [50] (4 GIH, 5 MXL, and 4 TSI), selected samples from the 1000 Genomes Project [51] that were sequenced to high coverage and typed on the Complete Genomics platform (5 CEU, 5 LWK, 4 PEL, 5 PJL, and 4 YRI), and from samples used in Schlebusch et al. [26] (5 JUH, 4 KAR, 5 NAM, and 4 XUN) in order to obtain coverage worldwide, with 4–5 individuals per population (Table 1). The populations were assigned to six global pools of nine individuals each – Northern Khoe-San (NKS = JUH + XUN), Southern Khoe-San (SKS = KAR + NAM), West African origin (WAF = YRI + LWK), admixed Indigenous American (AMR = PEL + MXL), South Asian (SAS = GIH + PJL), and European (EUR = CEU + TSI), based on previous investigations of population structure [26, 51, 52]. Levels of population structure of the pooled populations were assessed with a pairwise F_ST_ [53] (Additional file 1: Table S1), with all pooled groups having pairwise F_ST_ values <0.0156. For use in downstream analyses, human ancestral and derived alleles were determined for the variants found in the sequence data using three outgroups, Chimpanzee (panTro4), Gorilla (gorGor3) and Orangutan (ponAbe2); all downloaded from the UCSC genome browser (genome.ucsc.edu/index.html). For further details on how the data were processed, see Additional files.

### Gene annotations

Gene annotations were obtained from Ensembl version 75 (GRCh37.p13; GENCODE 19) (ftp://ftp.ensembl.org/pub/release-75/gtf/homo_sapiens/), with the exception of the intron annotation, which was obtained separately from the UCSC table browser (http://genome.ucsc.edu/cgi-bin/hgTables). More specifically, genomic coordinates were retrieved for CDS, UTR, and INTRONs.

### Regulatory annotations

The regulatory element dataset was based on annotations generated by the ENCODE Project [7]. Experimental data from the three tier-1 cell lines (GM12878, H1-hESC, and K562) and the two tier-2 cell lines (HeLa-S3 and HepG2) were used; including DHS peaks, genome segmentations, and transcription factor binding site (TFBS) peaks. DHS peaks [30], were accessed through the ENCODE Project portal (www.encodeproject.org). Uniform TFBS peaks [54] and predicted functional elements from the combined genome segmentation [42] were downloaded from the ENCODE section of the UCSC genome browser (uniform peaks: http://genome.ucsc.edu/cgi-bin/hgFileUi?db=hg19&g=wgEncodeAwgTfbsUniform; genome segmentations: http://genome.ucsc.edu/cgi-bin/hgFileUi?db=hg19&g=wgEncodeAwgSegmentation).

Annotation files for DHS peaks, genome segmentations, and TFBS peaks were limited to chromosomes 1 to 22, and annotations for the five cell lines (GM12878, H1-hESC, K562, HeLa-S3 and HepG2) were combined and sorted. This was performed using BEDTools version 2.23.0 [55].

### Transcription factor classes

TFBS peaks from a total of 136 TFs were used in the analyses (Additional file 2: Table S2). These included the initial dataset used in the ENCODE integrative analysis [7, 54], as well as additional TFs included prior to the March 2012 freeze. The TFBS peaks, which denote regions of DNA sequence shown to have been bound by specific TFs, were derived from chromatin immuno-precipitation and high-throughput sequencing (ChIP-seq) data [54]. Sequence-specific TFs were classified by Gerstein et al. [54] into families, based on the Luscombe dataset [56] and DNA binding domain data from Interpro (https://www.ebi.ac.uk/interpro/). This information was used in the current study, with additional TFs classified based on the information found in Gerstein et al. [54], and using the PANTHER Classification System (http://pantherdb.org/) [57, 58]. Families with more than five representative TFs in the dataset were also pooled into categories (Additional file 2: Table S2).

### Decomposition of site frequency spectra

In order to uncover the presence of selection on genomic elements, summary statistics were computed across the six global population groups, which allowed us to examine components of the SFS; whose features are often utilised to examine how population-level processes shape the genetic variation within a group. These included average number of pairwise nucleotide differences, *θ*_*π*_ [59], as well as the neutrality test statistic, Tajima’s D [60]. Estimates of means and standard error for Tajima’s D, and *θ*_*π*_ were generated using the weighted block jackknife approach [61, 62], with a five megabase genomic block consecutively removed at each iteration, using custom python scripts.

### Comparing neutral and non-neutral regions using a two-sample Z-test

Selection, however, is not the only population-level process capable of affecting the SFS. Demographic effects also contribute substantially to shaping the SFS; working in conjunction with selection to affect SFS summary statistics [21]. In order to use SFS summary statistics to uncover selection at genomic elements, it was necessary to account for the effect of demography in shaping the signal. This is a difficult task and much population genetic research is aimed at this endeavour [21, 63]. Our approach was to compute SFS summary statistics for a selection-neutral reference as well; comprised of non-annotated sequence. This class was filtered to exclude the annotations mentioned above, as well as non-coding RNA and pseudogenes (from Ensembl version 75), and high occupancy target regions [64]; while being at least 200 kb away from protein coding sequence, and so would presumably be under minimal selection, relative to the rest of the genome. While this would not completely remove all of the effects of selection from the reference signal, by removing the most intense signals of selection, we could provide at least a conservative estimate for levels of selection on genomic elements in comparison to the selection-neutral reference. A two-sample Z-test (genomic element versus non-annotated sequence) for the SFS summary statistics was conducted; providing a statistical measure (effect size) of the level of selection, in the form of a Z-score. Since the normality assumption breaks down for values of |Z| > 2 [65], the significance threshold was set at *p* < 0.05, which is equivalent to a Z-score > 1.96 or a Z-score < −1.96. Z-scores above or below these values, while statistically significant, are not translatable to *p*-values.

### MK test for neutrality

A modified version of the MK test was used as an additional measure of neutrality. Polymorphisms (P) were counted as the number of polymorphic variants in accessible sequence from the combined sample, while substitutions (D) were calculated based on the number of derived alleles that were fixed on the modern human lineage, compared to the three primate outgroups. The postulated non-neutral (n) functional elements were compared to a neutral (s) reference; the non-annotated sequence class. The NI was calculated using [66]:

$$ NI=\frac{P_n/{P}_s}{D_n/{D}_s} $$

### Sliding window correlation analysis

Correlations of the locations of genomic and regulatory elements were computed. A sliding window approach [31] was used in order to incorporate the spatial distribution of elements. The chromosomes were annotated per site for the presence/absence (1/0) of selected element classes. These elements were then mapped to windows, with a step-size of 1.5 kb, as count data per window for each element class. Window sizes of 5 kb, 10 kb and 15 kb were compared (Additional file 1: Table S4). Negligible differences were found due to window size, with extremely high levels of positive correlation (0.959–0.998) between window sizes, and we only show the results from 10 kb windows. The resultant data matrices were used to perform a pairwise correlation analysis (using Pearson’s r) of the element classes. The pairwise correlation matrices were visualised in R using the “corrplot” package; and ordered via hierarchical clustering (method = ward.D).

### Disentangling purifying- and linked-purifying selection

In order to assess the distance that linked-purifying selection extends from CDS under purifying selection, discrete bins of non-annotated sequence were compiled at increasing distance from CDS (i.e. 2.5 kb, 5 kb, 7.5 kb, 10 kb, 25 kb, 50 kb, 75 kb, and 100 kb away), using BEDTools version 2.23.0 [55] and BEDOPS version 2.4.3 [67]. The SFS summary statistics were then computed for each of these bins. These discrete bins were also generated for each of the genomic classes for which we had earlier obtained overall summary statistic estimates. This allowed us to compare the estimates for the genomic classes to that of non-annotated sequence within each bin.

## Additional files

Additional file 1: Supplementary Methods, Tables and Figures. (PDF 8572 kb)

Additional file 2: **Table S2.** Transcription factors used in the study. (XLSX 17 kb)

## Abbreviations

AFR: African
AMR: Admixed Indigenous American
BHLH_FAM: Basic helix-loop-helix protein family
BZIP_FAM: Basic leucine zipper family
CDS: protein coding sequence regions
CEU: Northern & Western European ancestry
ChIP-seq: Chromatin immuno-precipitation and high-throughput sequencing
cM: centimorgan
CTCF_ENRICHED: CTCF enriched elements
DHS: DNase hypersensitive regions
ENHANCER: predicted enhancer
EUR: European origin
GIH: Gujarati ancestry
HMD_FAM: Homeobox-domain family
INTRON: protein coding intronic regions
JUH: Ju/‘hoansi
kb: kilobases
kya: thousand years ago
LWK: Luhya
MK: McDonald–Kreitman
MXL: Mexican ancestry
N-AFR: Non-African
NAM: Nama
NI: Neutrality index
NKS: Northern Khoe-San
NON_ANN: Non-annotated regions at least 200 kb away from CDS
NR_FAM: Nuclear hormone receptor family
P53_FAM: P53-like transcription factor family
PEL: Peruvian
PJL: Punjabi
PROMOTER_FLANK: predicted promoter flanking region
PROMOTER_w_TSS: predicted promoter region including transcription start site
SAS: South Asian
SFS: Site frequency spectrum
SKS: Southern Khoe-San
TF: Transcription factor
TFBS: Transcription factor binding site
TFCHR: chromatin-modifying transcription factors
TFGEN: general transcription factors
TFSS: sequence-specific transcription factors
TSI: Italian
UTR: protein coding untranslated regions
W_ENHANCER: predicted weak enhancer
WAF: West African origin
WHOLE_GENOME: Whole genome average
WHTH_FAM: Winged helix-turn-helix family
XUN: !Xun
YRI: Yoruba
ZNF_FAM: Zinc finger protein family
*θ*_*π*_: Average number of pairwise nucleotide differences
