# Supplementary material for: Patterns of variation in cis-regulatory regions: examining evidence of purifying selection
Source: BMC Genomics. 2018 Jan 26;19:95. doi: 10.1186/s12864-017-4422-y (PMC5787233; doi:10.1186/s12864-017-4422-y)
Supplement: Supplementary file 1 — Supplementary Methods, Tables and Figures. (PDF 8572 kb) [file 12864_2017_4422_MOESM1_ESM.pdf]

Supplementary materials for

Patterns of variation in cis-regulatory regions: examining evidence of purifying selection

Thijessen Naidoo[1], Per Sjödin[1], Carina Schlebusch[1], Mattias Jakobsson[1,2*]

[1] Department of Organismal Biology, Uppsala University, Uppsala, Sweden

[2] Science for Life Lab, Uppsala, Sweden

* correspondence: mattias.jakobsson@ebc.uu.se

Data preparation:

The SNP data utilised in this study is a subset of the data prepared for Schlebusch et al. (in prep.), where DNA samples from individuals were collected with the subjects' informed consent, and the project was approved by the Human Research Ethics Committee (Medical) at the University of the Witwatersrand, Johannesburg (Protocol Number: M1604104), the Working Group of Indigenous Minorities in Southern Africa (WIMSA) and the South African San Council (SASC).

Briefly, DNA libraries of the Khoe-San samples (5 JUH, 4 KAR, 5 NAM, and 4 XUN) were prepared with TrueSeq® DNA Sample preparation kit v2 (cat#FC-121-2001/2002, Illumina Inc.). These were sequenced on an Illumina HiSeq Sequencing System (Illumina Inc., San Diego, CA.) at the SciLife Lab SNP & Seq platform in Uppsala. Bam files were generated by mapping the reads to the 1000 genomes phase 2 reference assembly (hs37d5) using BWA 0.6.2 (BWA-MEM algorithm), and further processed with GATK v.2.5.2, Picard v.1.92 and Samtools. This involved duplicate marking in Picard, realignment around indels in GATK, calculating the MD flag with Samtools calmd and Base Quality Score Recalibration (BQSR) in GATK. SNP calling was performed using the UnifiedGenotyper module in GATK. Sites were filtered following variant quality score recalibrations (VQSR) in GATK, for hardy weinberg equilibrium (HWE), and for at most, 10% missingness. Sequences were high coverage, with an average depth of 56X (Schlebusch et al., in prep.)

Selected samples from the Complete Genomics diversity set and from the 1000 Genomes Project that were sequenced to high coverage and typed on the Complete Genomics platform were downloaded in vcf format from ftp://ftp2.completegenomics.com/vcf_files/Build37_2.0.0/ and http://www.1000genomes.org/announcements/complete-genomics-data-release-2013-07-26, respectively. Files were processed to keep only SNP sites, only individual genotype information was extracted and missing stretches were extended to one line per position. Sites were set as missing if they: were half-called genotypes; were marked as VQLow; were below a depth of 10 and had a genotype quality of 30; and failed a one-sided HWE test at p-value that removed all completely heterozygous sites (calculated per dataset - actual value depends on size of dataset). All sites were kept to allow for merging with other comparative data. Merging procedures were performed using VCFTools v. 0.1.11 vcf-merge (Schlebusch et al., in prep.).

Human ancestral and derived alleles were determined for the SNPs using three outgroups: Chimpanzee (panTro4), Gorilla (gorGor3) and Orangutan (ponAbe2); all downloaded from the UCSC genome browser (genome.ucsc.edu/index.html). For use in the downstream analyses, only sites with an A, C, G or T ancestral allele, and ancestral allele support of 2 or 3 (2 or 3 of the 3 great apes showed the same variant) were chosen.

Strategy for normalising SFS summary statistics

SFS summary statistic estimates taken from different regions of the genome will be affected both by selection and demography. Thus, when using the SFS to search for signals of selection among genomic elements it is necessary to take steps to isolate the true signal from other contributing factors. We used the following strategy to accomplish this:

1. We computed SFS summary statistics (average number of pairwise nucleotide differences, θ_π , and Tajima's D) for several genomic classes.

Accounting for demography:

2. We computed the same SFS summary statistics for a selection-neutral reference, comprised of non-annotated sequence that was at least 200kb away from protein coding sequence; assuming that the estimates obtained would be shaped mainly by demography.
3. We compared the results for the elements of interest to those of the selection-neutral reference using a two-sample Z-test, providing a statistical measure (effect size) of the level of selection on the element, in the form of a Z-score.

Accounting for linked-purifying selection:

4. We assessed the distance that linked-purifying selection extends from protein coding sequence under purifying selection by its effect on neutral non-annotated sequence.
5. We computed SFS summary statistics for discrete bins of non-annotated sequence at increasing distance from protein coding sequence (i.e. 2.5kb, 5kb, 7.5kb, 10kb, 25kb, 50kb, 75kb, and 100kb away). The patterns observed here were a result of the effect of linked-purifying selection on non-annotated sequence.
6. We then computed SFS summary statistics for the elements of interest in the same discrete bins at increasing distance from protein coding sequence.

7. We compared the results for the elements of interest to those of non-annotated sequence in each discrete bin using a two-sample Z-test; unhitching the effect of linked-purifying selection (from protein coding sequence) on the element.

Tables:

Table S1: Weir and Cockerham pairwise Fst within global pools

Global Pool	Population 1	Population 2	Pairwise Fst
NKS	JUH	XUN	0.0145783157
SKS	KAR	NAM	0.015039483
WAF	LWK	YRI	0.0071564576
AMR	MXL	PEL	0.015540374
EUR	CEU	TSI	0.0063865686
SAS	GIH	PJL	0.0024844216

Table S2: Transcription factors used in the study. See additional supplementary file "supplementary_table_2.xls"

Table S3: Pearson's r correlations of SFS summary statistics with association to coding sequence (CDS) and average genomic class size

	θ_π		Tajima's D	
Global Pool	CDS association*	Class Size	CDS association*	Class Size
NKS	−0.405377295655097	0.0661291901418119	−0.57112022869433	0.192025117961138
SKS	−0.418006693065093	0.067530667382393	−0.521083243511829	0.1464870376687
WAF	−0.479441628441071	0.0678984555469901	−0.651406468716945	0.176545669127443
AMR	−0.485187336295403	0.0651569081662575	−0.618304130977804	0.245881612752945
EUR	−0.487354872268102	0.060254561709697	−0.594646674262736	0.211844332215242
SAS	−0.487730903598439	0.0650737150065937	−0.580979087680242	0.232731882750272

*Derived from pairwise sliding-window correlation analysis on chromosome 1

Table S4: Pairwise Pearson's r correlations among window sizes 5kb, 10kb and 15kb on chromosomes 1 and 10

	CHR10_5KB	CHR10_10KB	CHR10_15KB	CHR1_5KB	CHR1_10KB	CHR1_15KB
CHR10_5KB	1	0.9956297587	0.9883554596	0.9763446572	0.9702714626	0.9590562861
CHR10_10KB	0.9956297587	1	0.9977983649	0.9686439172	0.9674369423	0.9587724037
CHR10_15KB	0.9883554596	0.9977983649	1	0.9620192457	0.9658382581	0.9609383344
CHR1_5KB	0.9763446572	0.9686439172	0.9620192457	1	0.9964082262	0.9885086191
CHR1_10KB	0.9702714626	0.9674369423	0.9658382581	0.9964082262	1	0.997530495
CHR1_15KB	0.9590562861	0.9587724037	0.9609383344	0.9885086191	0.997530495	1

Correlations were computed based on CDS association derived from the pairwise sliding-window correlation analysis

Figures

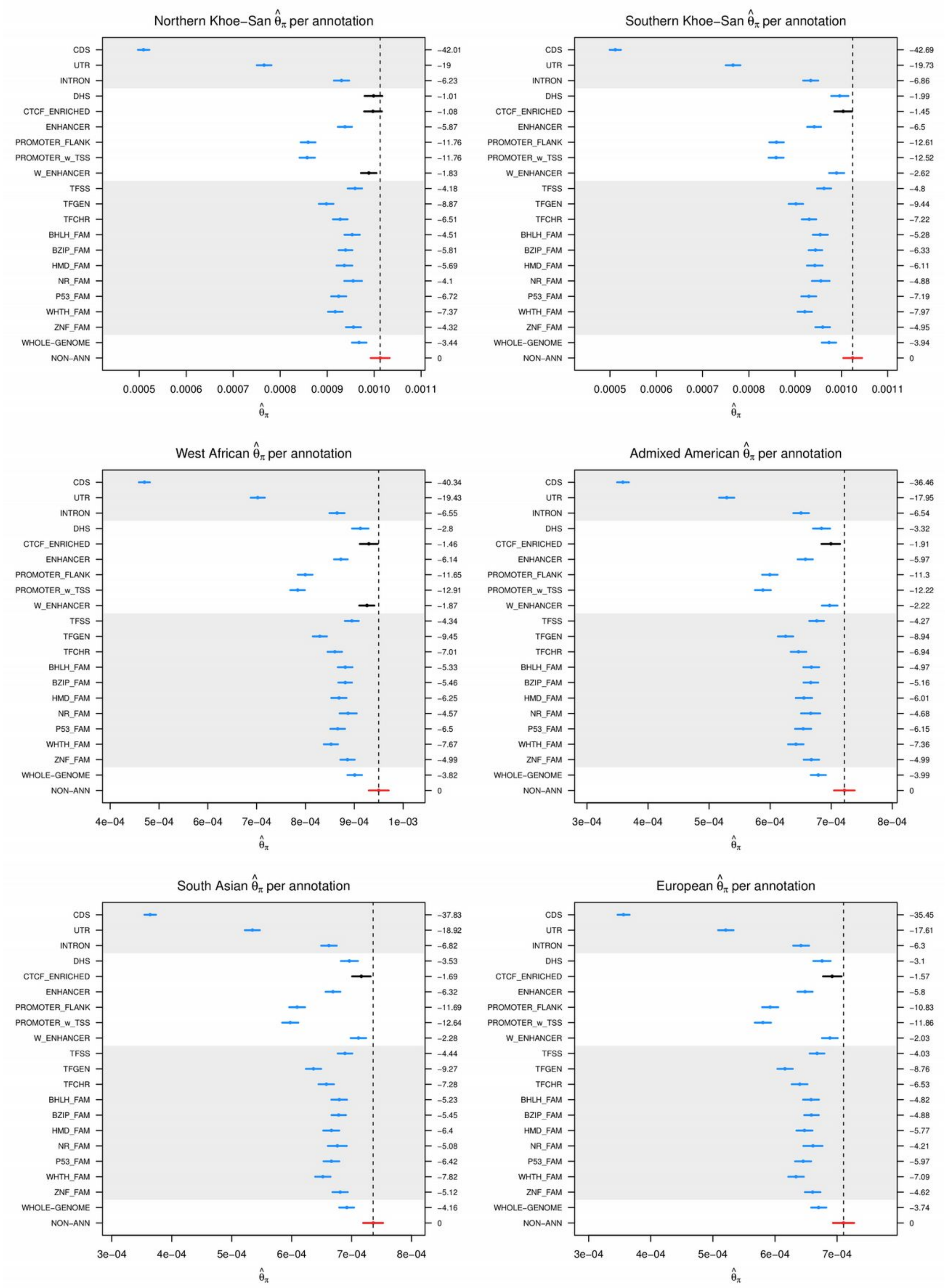

Figure S1: Estimated means and 95% confidence intervals of θ_π in the six global pools for genomic classes across genome. The threshold for significant difference to non-annotated sequence was set at $p < 0.05$ ($Z < -1.96$ or $Z > 1.96$).

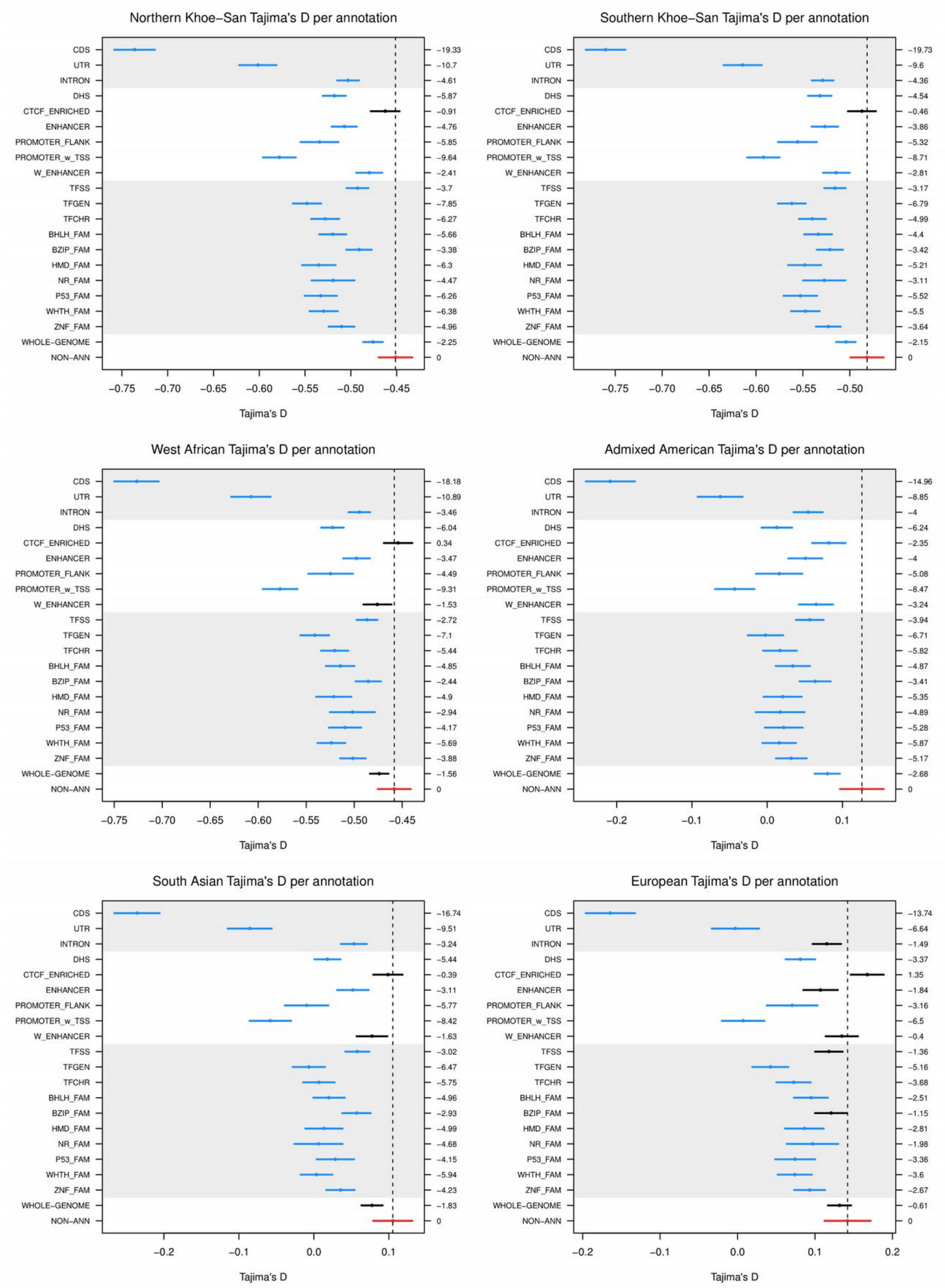

Figure S2: Estimated means and 95% confidence intervals of Tajima's D in the six global pools for genomic classes across genome. The threshold for significant difference to non-annotated sequence was set at $p < 0.05$ ($Z < -1.96$ or $Z > 1.96$).

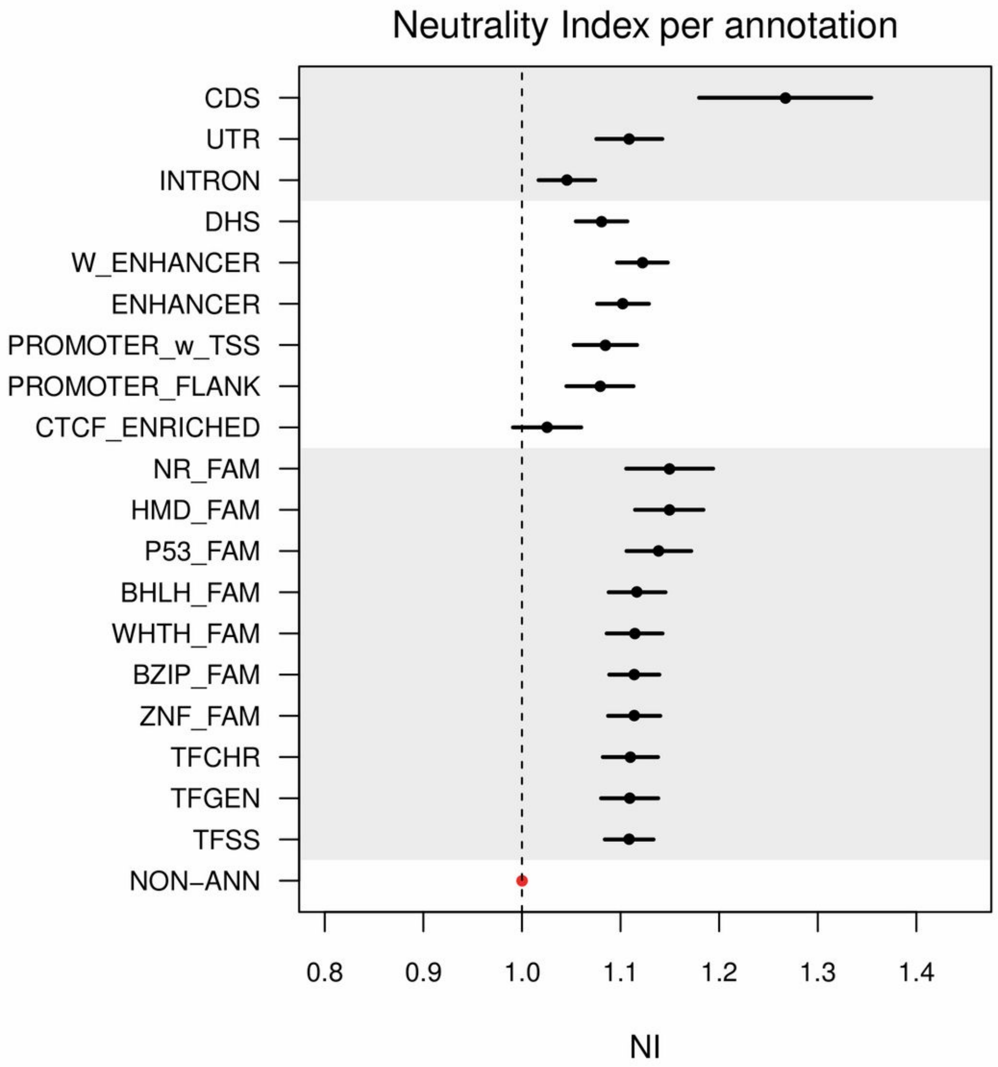

Figure S3: Neutrality Index (NI) per annotation category. Non-annotated sequence (NON-ANN) was used as the neutral reference.

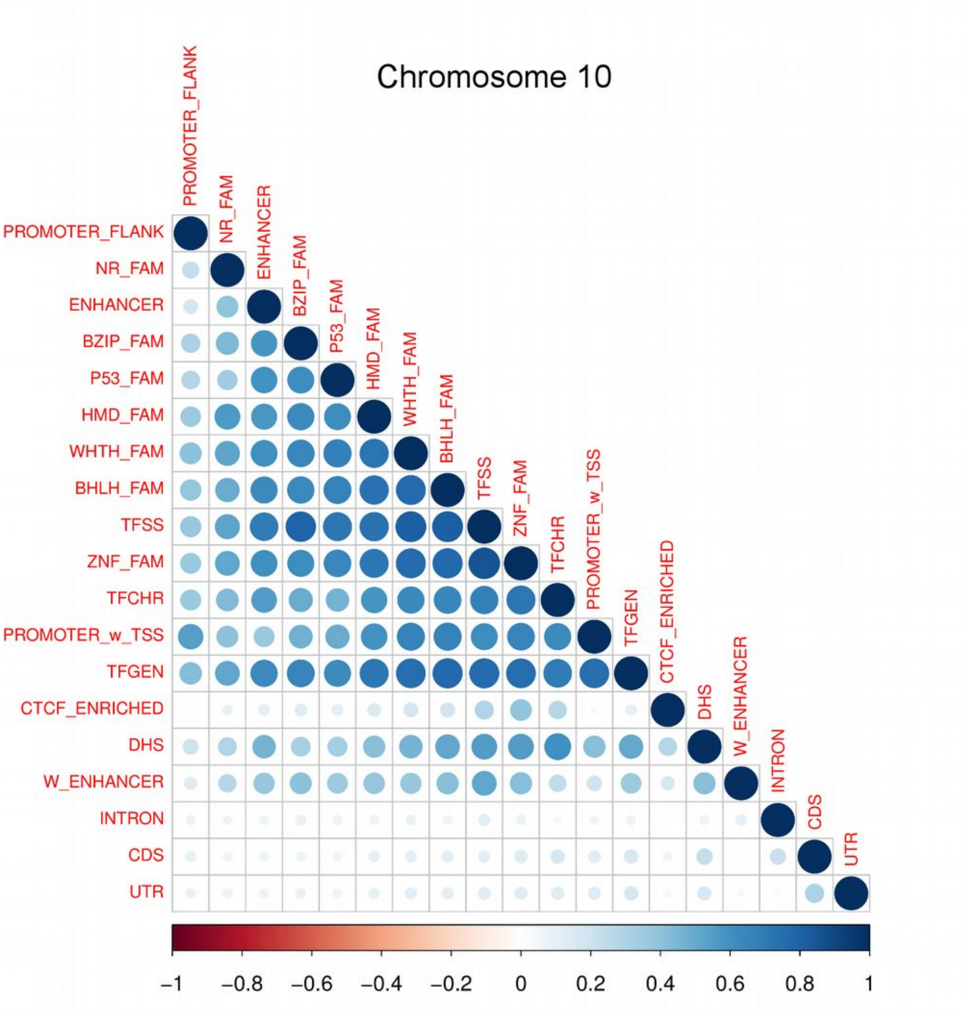

Figure S4: Pairwise correlations (Pearson's r) for genomic and regulatory classes on chromosome 10.

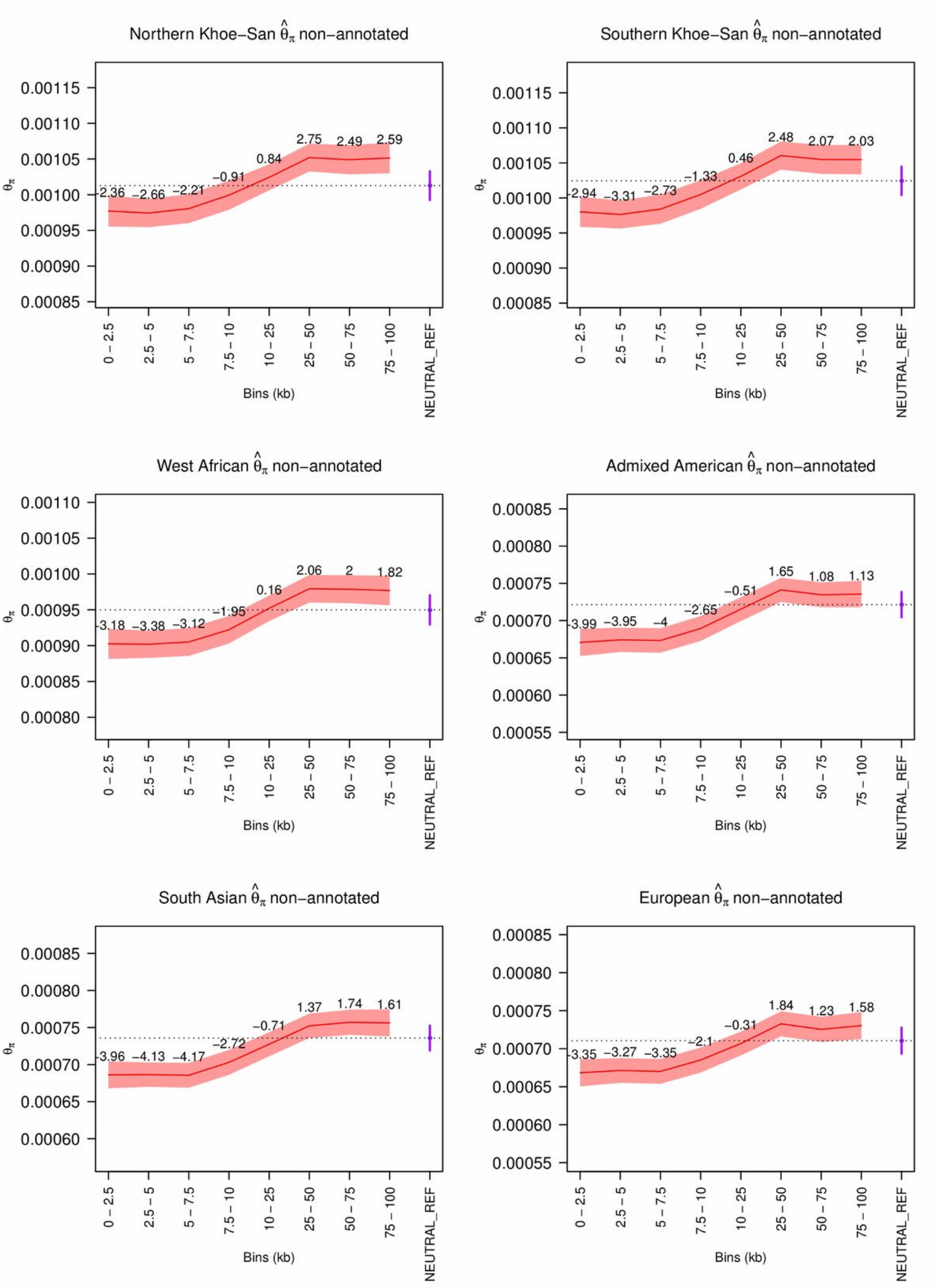

Figure S5: θ_π estimates computed for discrete bins of non-annotated sequence, at increasing distance from CDS up to 100kb, in the six global pools. Z-scores shown per bin represent size of difference to the NON-ANN neutral reference, shown in purple.

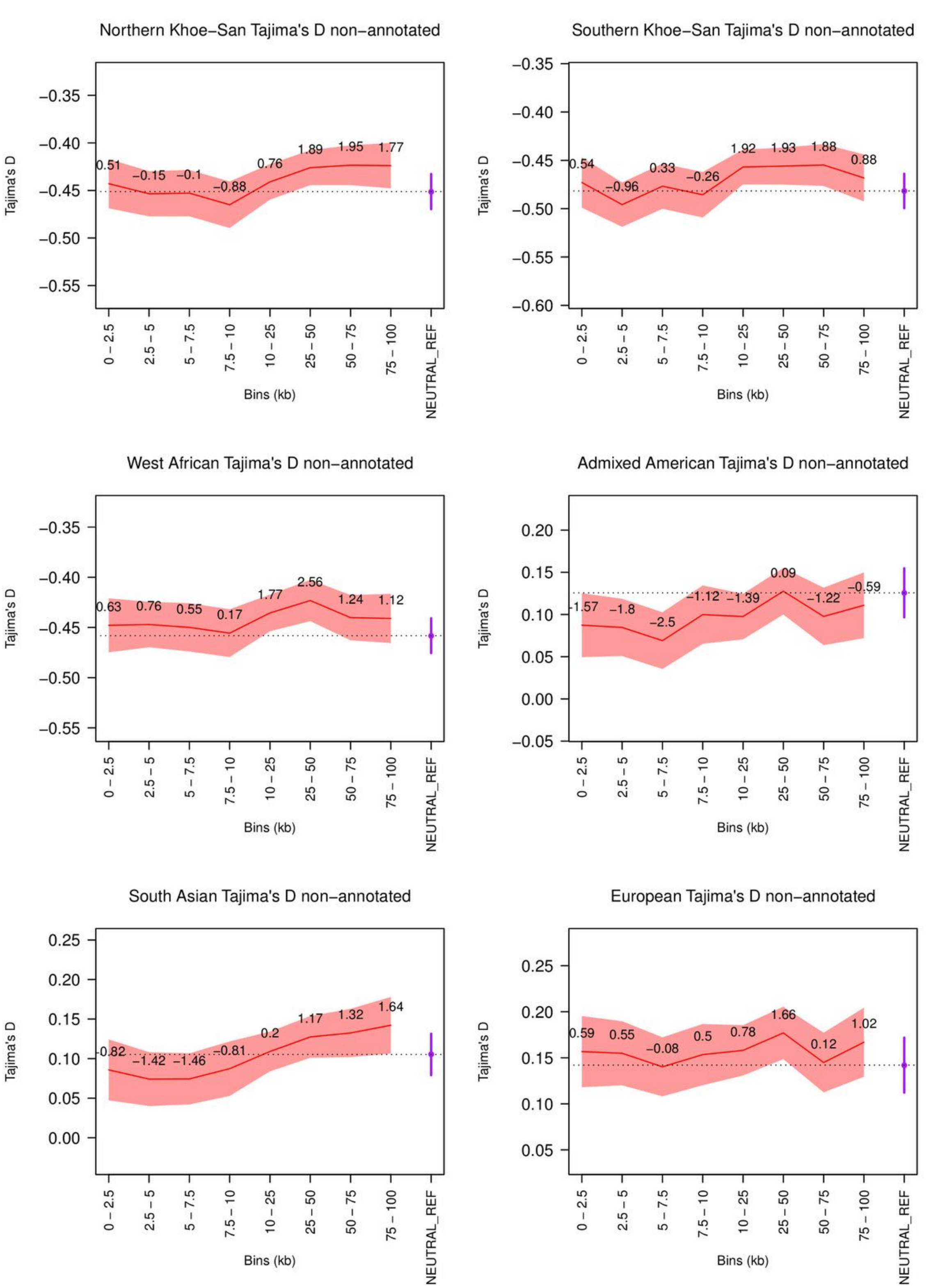

Figure S6: Tajima’s D estimates computed for discrete bins of non-annotated sequence, at increasing distance from CDS up to 100kb, in the six global pools. Z-scores shown per bin represent size of difference to the NON-ANN neutral reference, shown in purple.

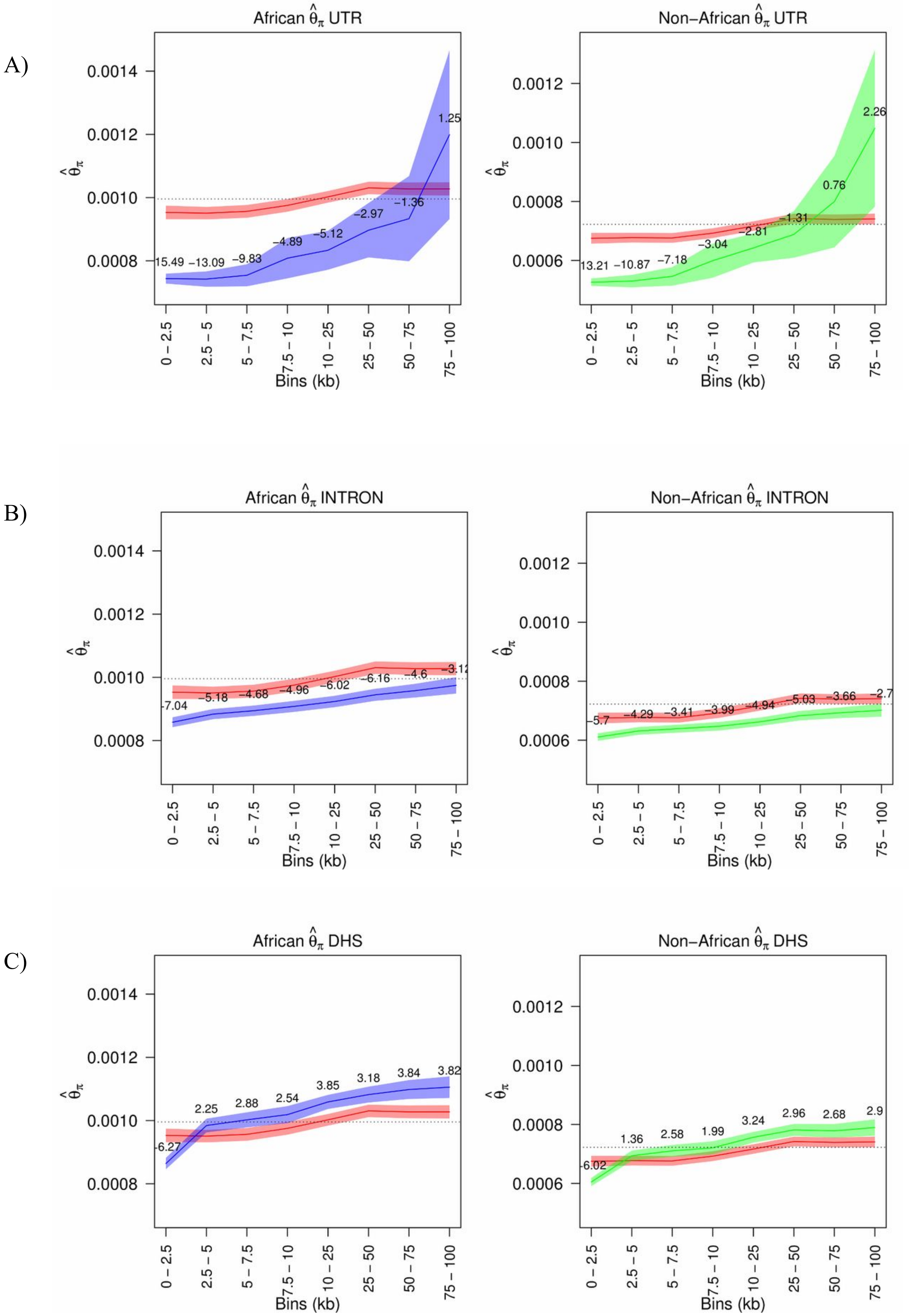

Figure S7: θ_π at varying distance from CDS for non-annotated sequence (red) versus (A) UTR, (B) introns, and (C) DHS, in African (blue) and non-African (green) populations. Neutral reference is illustrated by dotted line. Shaded areas represent 95% confidence intervals, with Z-scores (non-annotated vs. annotation) shown per bin.

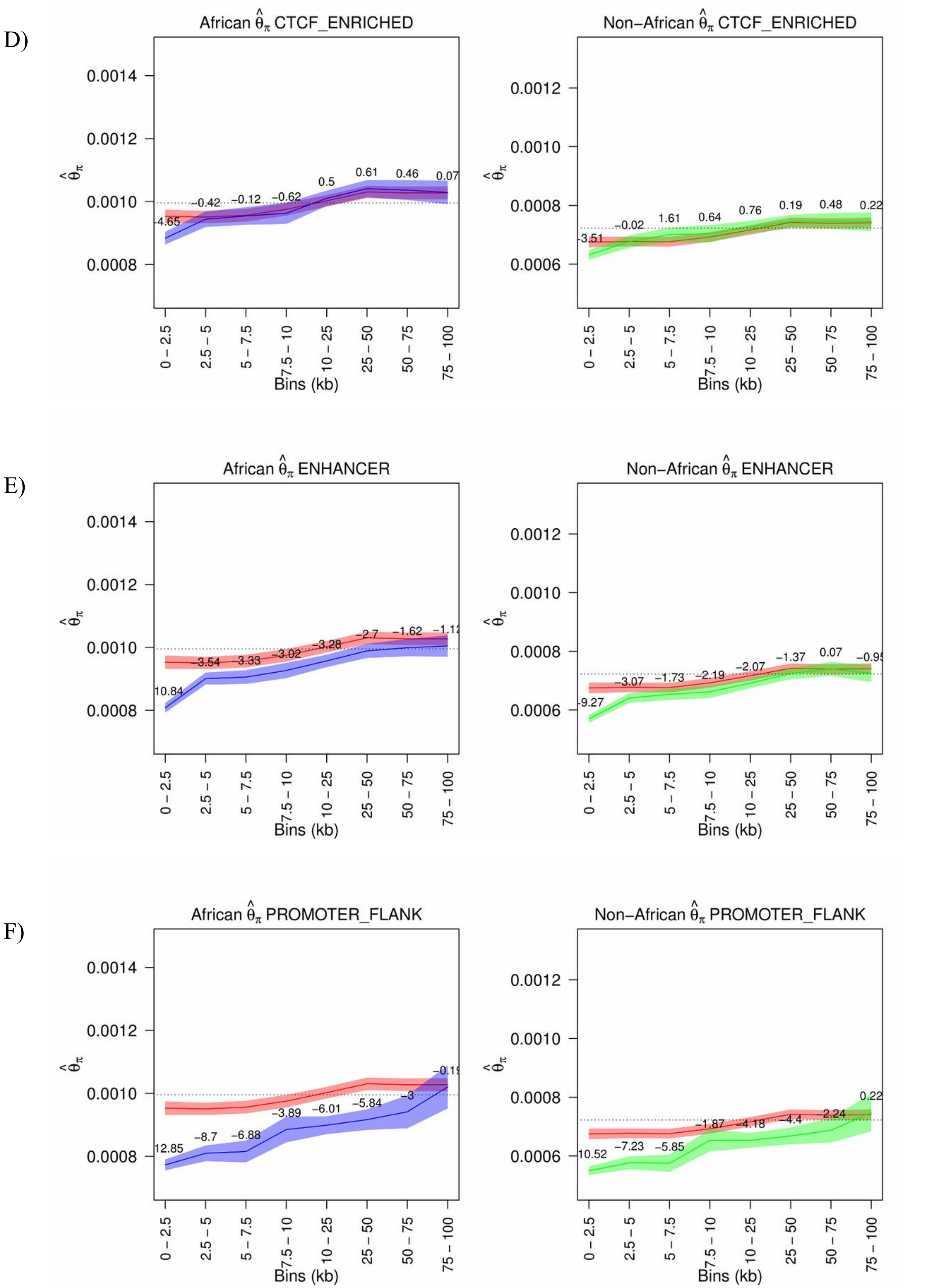

Figure S7: θ_π at varying distance from CDS for non-annotated sequence (red) versus (D) CTCF binding sites, (E) enhancers, and (F) promoter flanking regions, in African (blue) and non-African (green) populations. Neutral reference is illustrated by dotted line. Shaded areas represent 95% confidence intervals, with Z-scores (non-annotated vs. annotation) shown per bin.

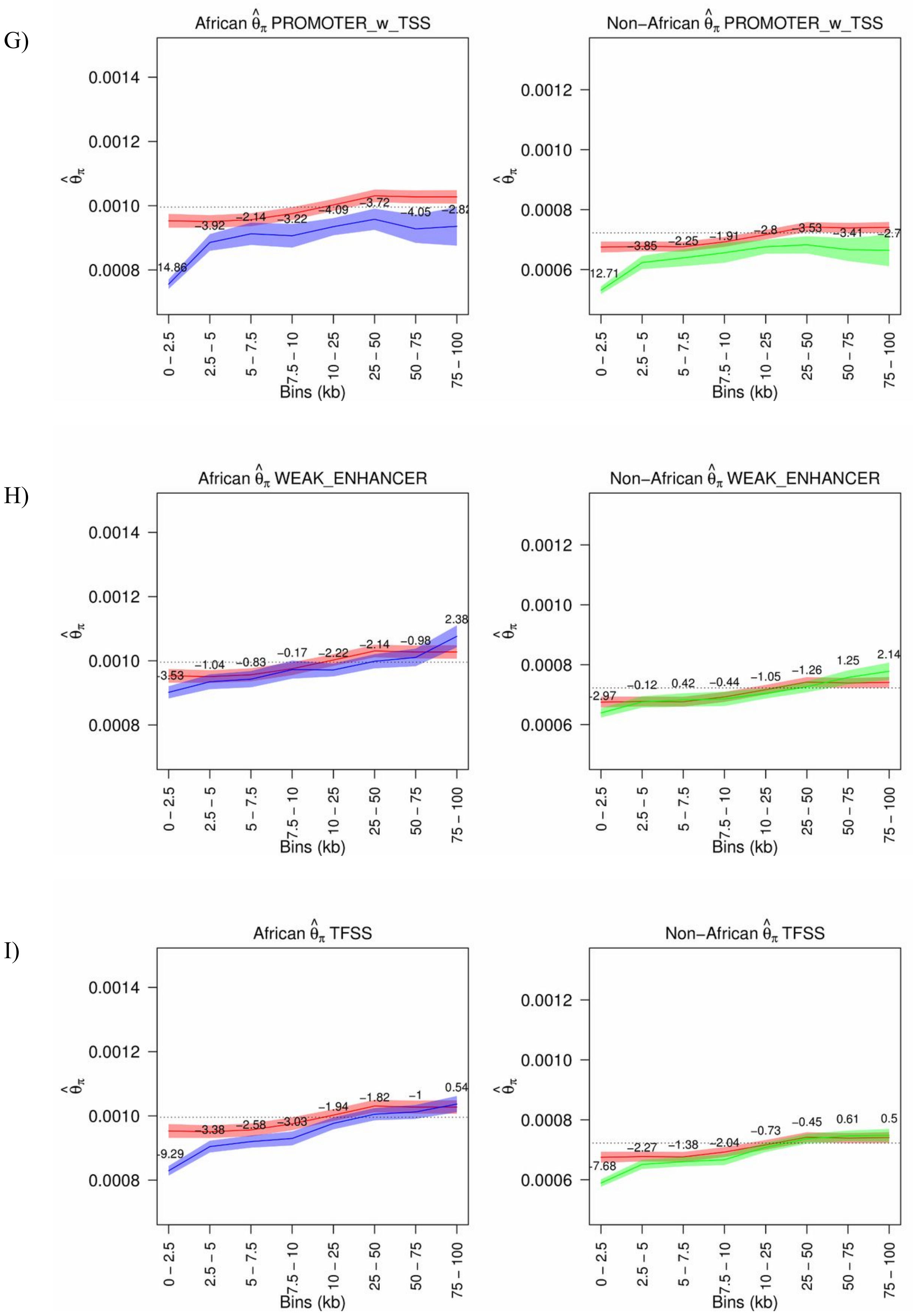

Figure S7: θ_π at varying distance from CDS for non-annotated sequence (red) versus (G) promoters, (H) weak enhancers, and (I) sequence-specific TFs, in African (blue) and non-African (green) populations. Neutral reference is illustrated by dotted line. Shaded areas represent 95% confidence intervals, with Z-scores (non-annotated vs. annotation) shown per bin.

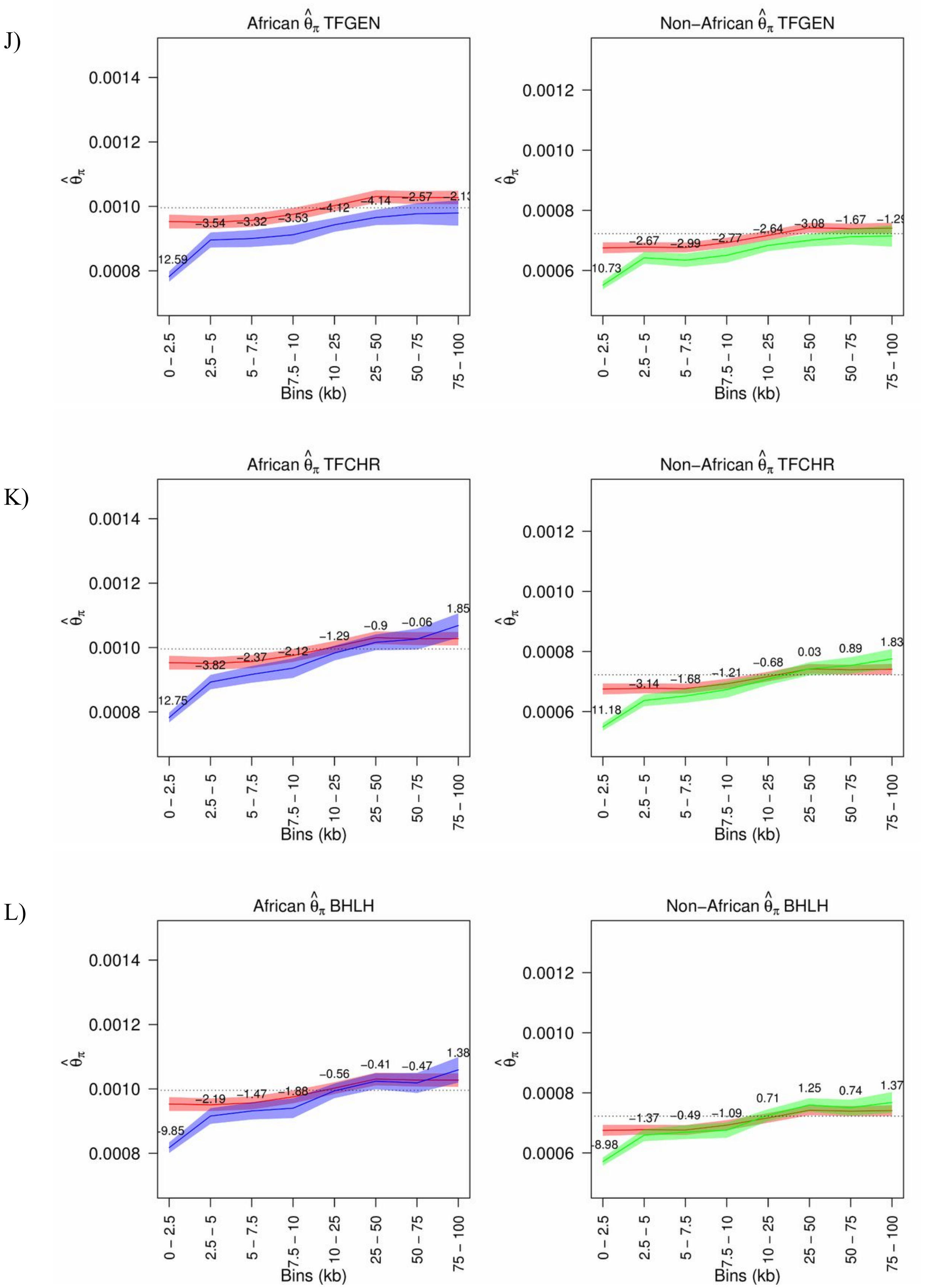

Figure S7: θ_π at varying distance from CDS for non-annotated sequence (red) versus (J) general TFs, (K) chromatin-modifying TFs, and (L) bHLH family TFs, in African (blue) and non-African (green) populations. Neutral reference is illustrated by dotted line. Shaded areas represent 95% confidence intervals, with Z-scores (non-annotated vs. annotation) shown per bin.

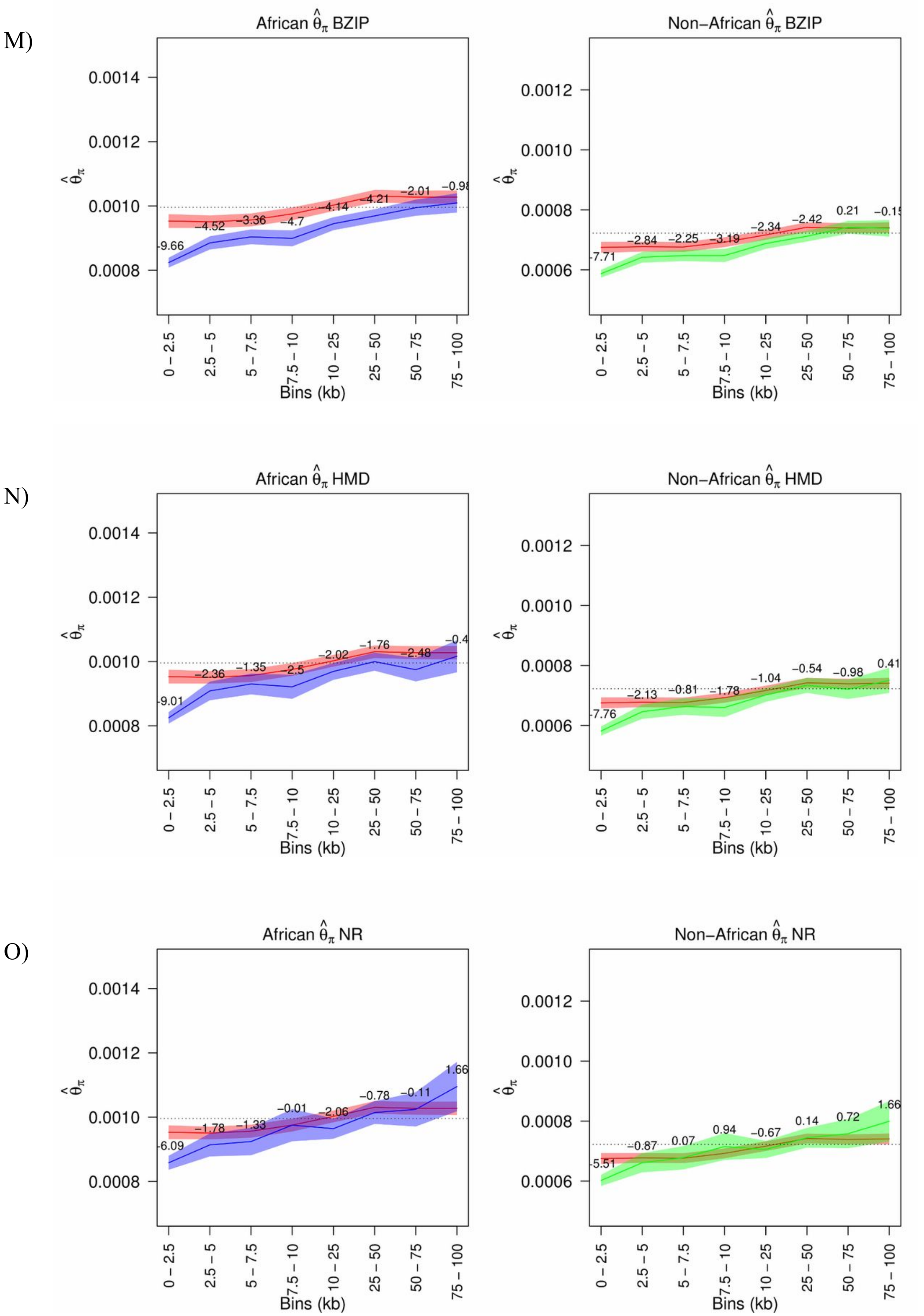

Figure S7: θ_π at varying distance from CDS for non-annotated sequence (red) versus (M) bZIP family TFs, (N) HMD family TFs, and (O) NR family TFs, in African (blue) and non-African (green) populations. Neutral reference is illustrated by dotted line. Shaded areas represent 95% confidence intervals, with Z-scores (non-annotated vs. annotation) shown per bin.

P)

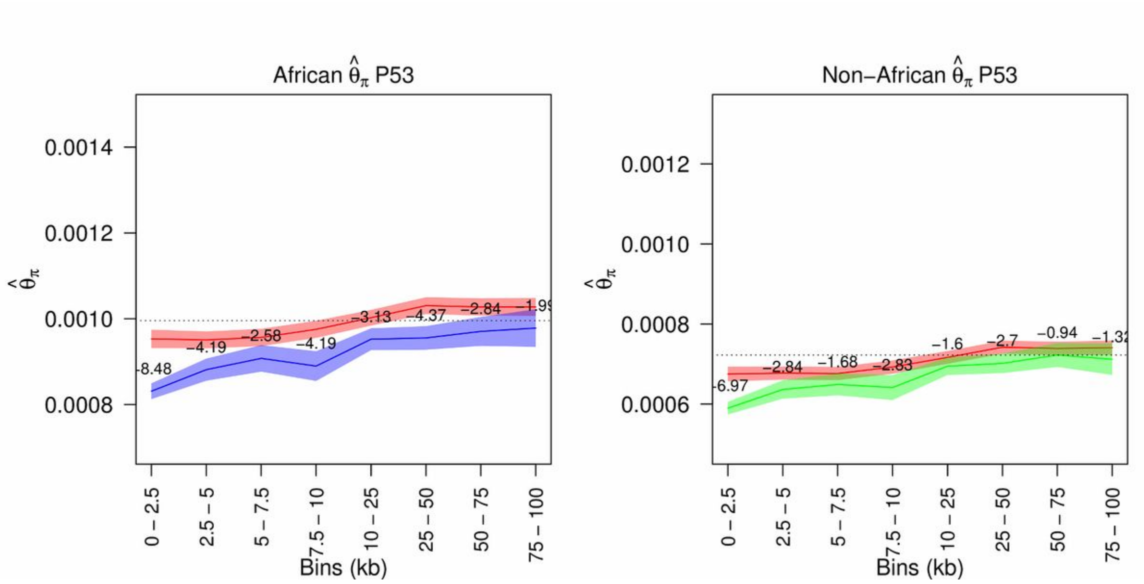

Q)

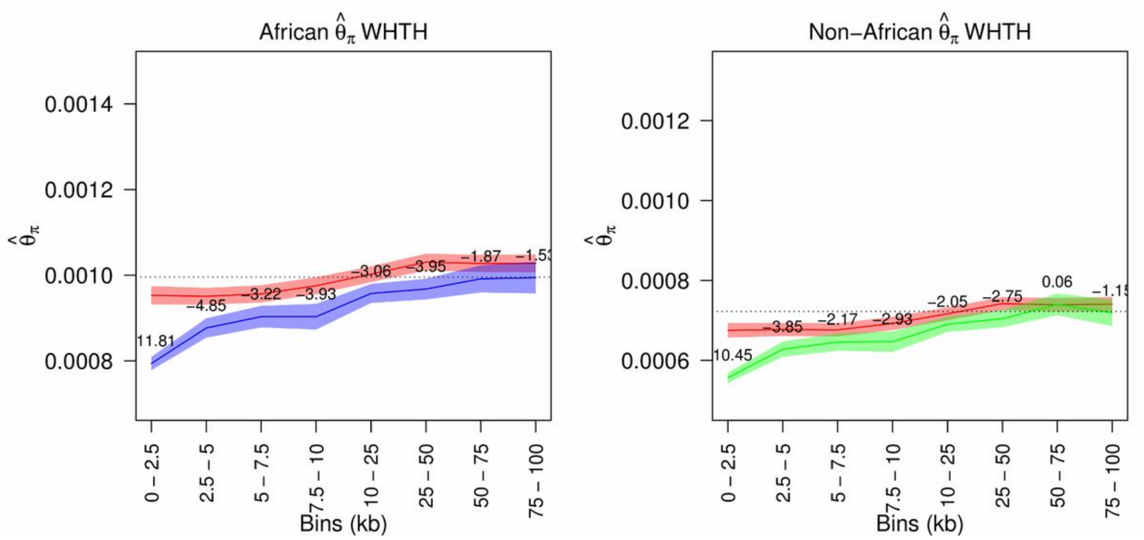

R)

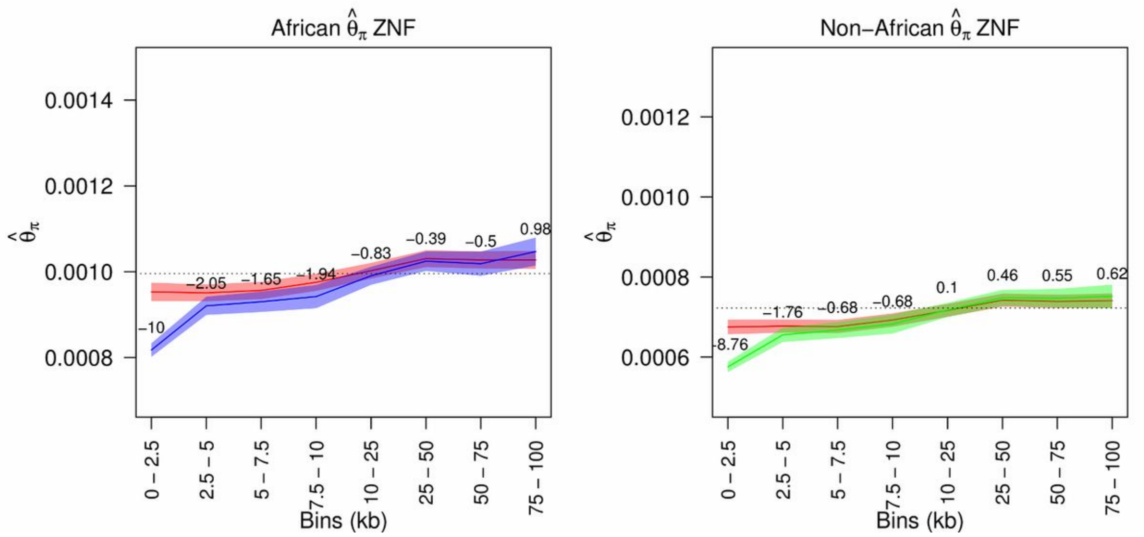

Figure S7: θ_π at varying distance from CDS for non-annotated sequence (red) versus (P) P53 family TFs, (Q) wHTH family TFs, and (R) ZNF family TFs, in African (blue) and non-African (green) populations. Neutral reference is illustrated by dotted line. Shaded areas represent 95% confidence intervals, with Z-scores (non-annotated vs. annotation) shown per bin.

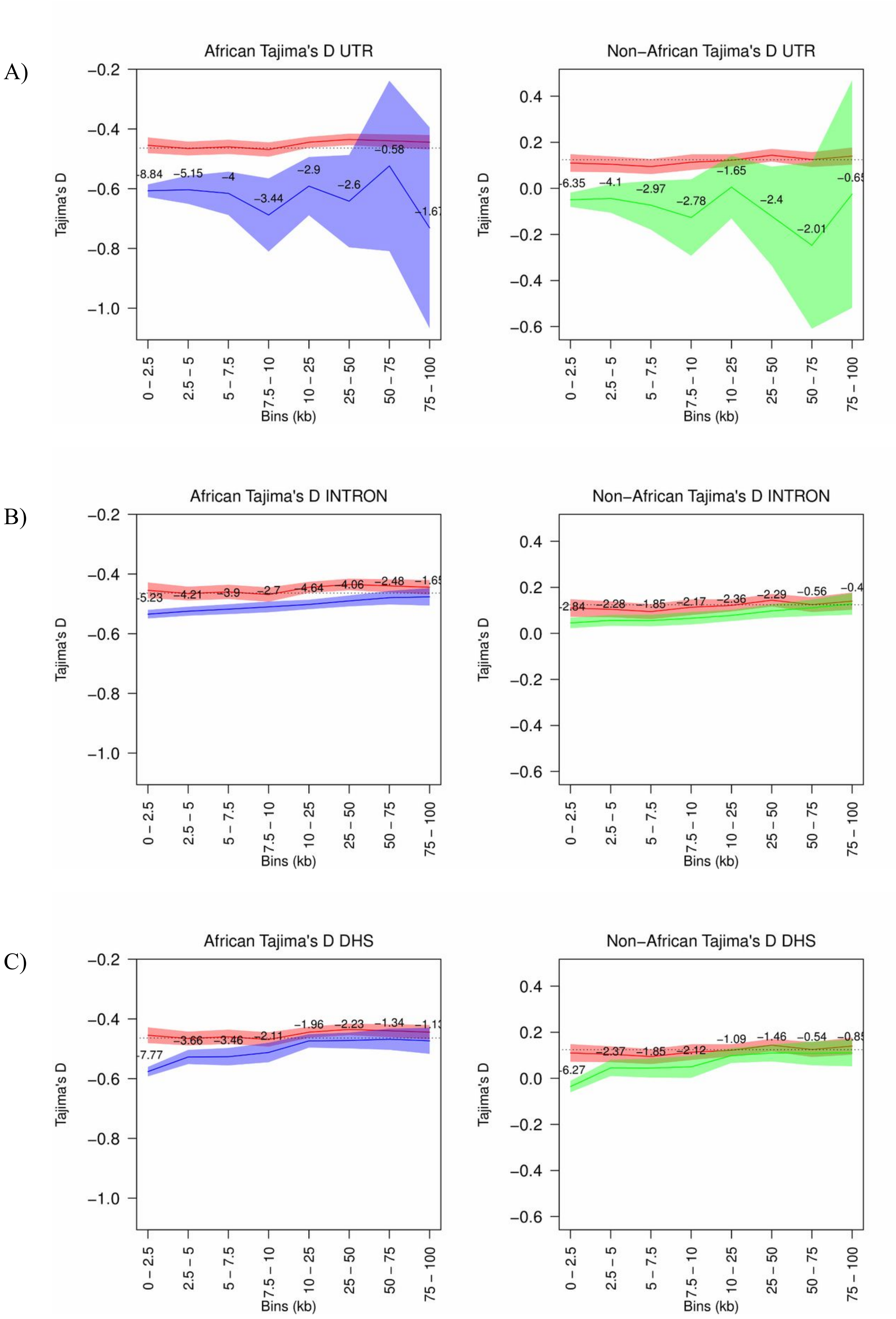

Figure S8: Tajima's D at varying distance from CDS for non-annotated sequence (red) versus (A) UTR, (B) introns, and (C) DHS, in African (blue) and non-African (green) populations. Neutral reference is illustrated by dotted line. Shaded areas represent 95% confidence intervals, with Z-scores (non-annotated vs. annotation) shown per bin.

D)

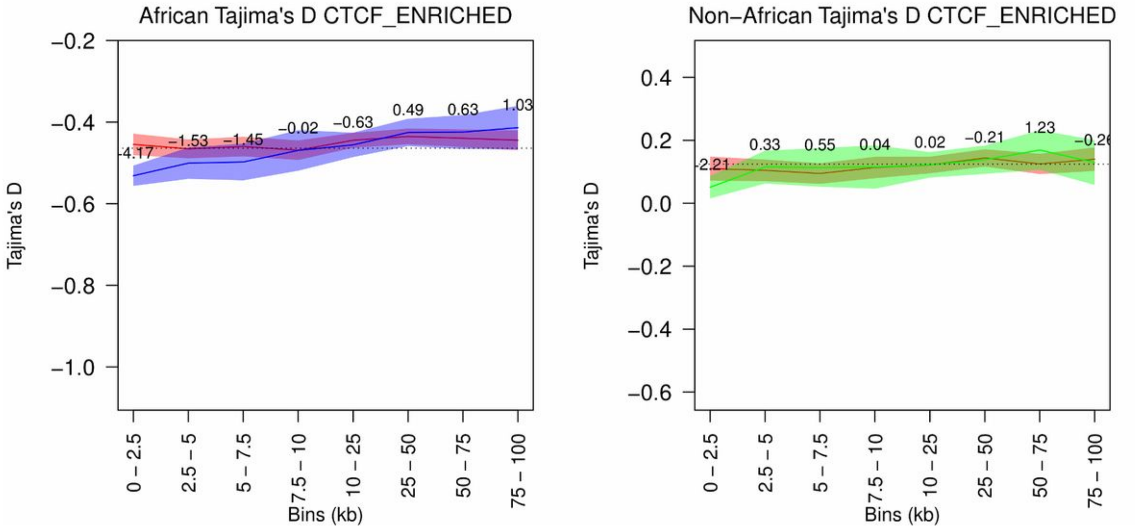

E)

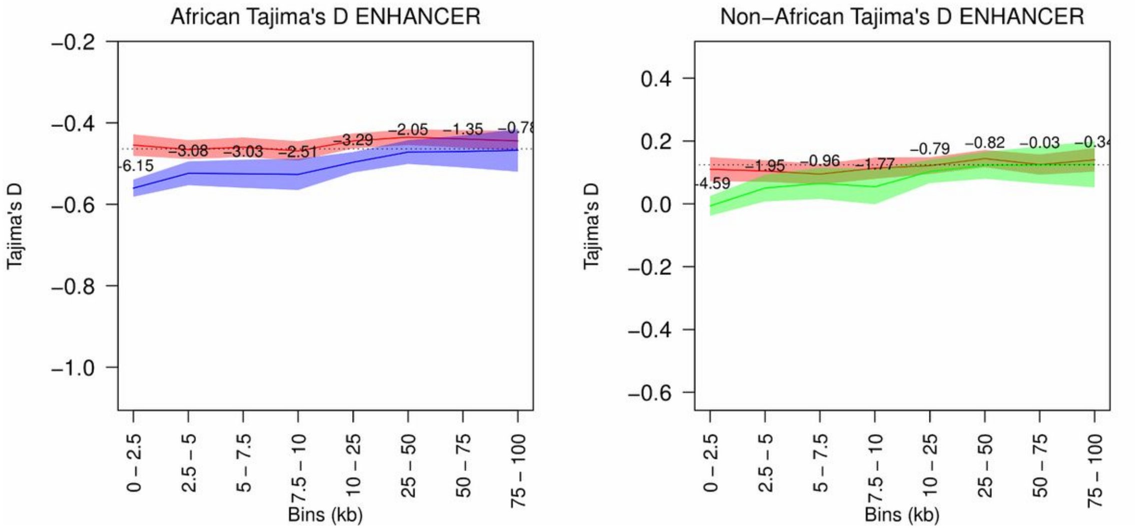

F)

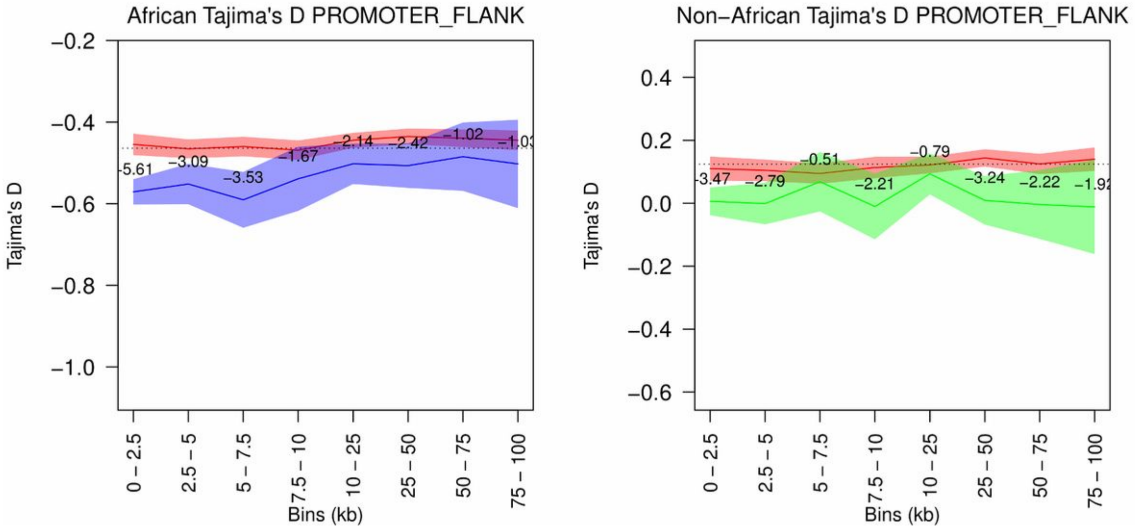

Figure S8: Tajima's D at varying distance from CDS for non-annotated sequence (red) versus (D) CTCF binding sites, (E) enhancers, and (F) promoter flanking regions, in African (blue) and non-African (green) populations. Neutral reference is illustrated by dotted line. Shaded areas represent 95% confidence intervals, with Z-scores (non-annotated vs. annotation) shown per bin.

G)

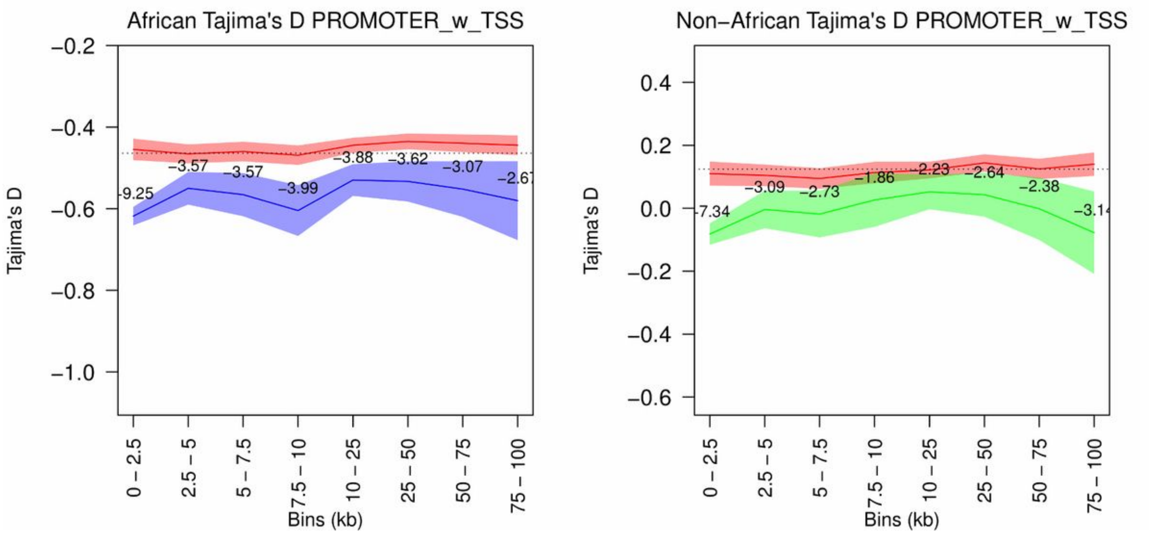

H)

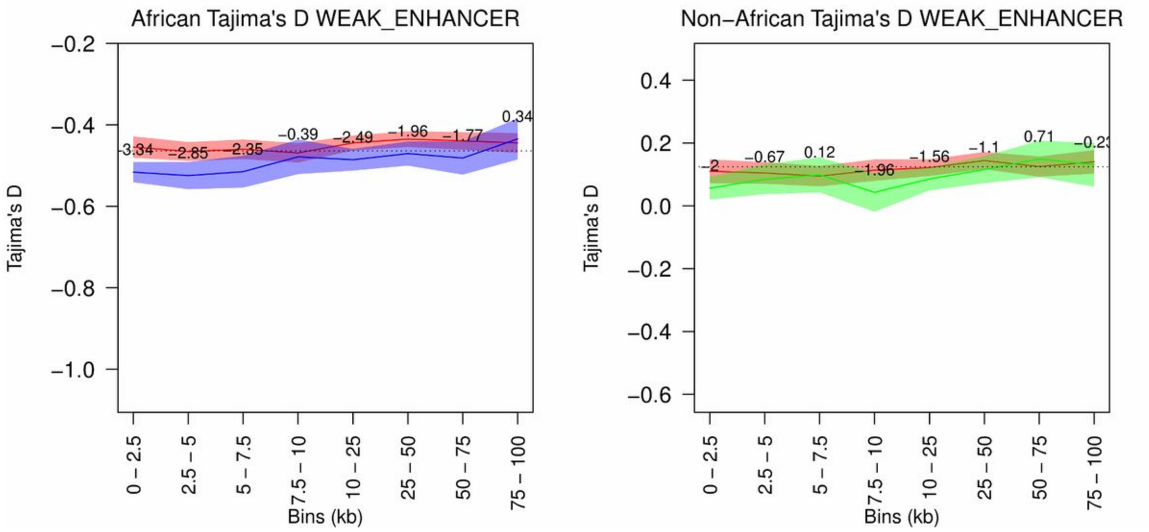

I)

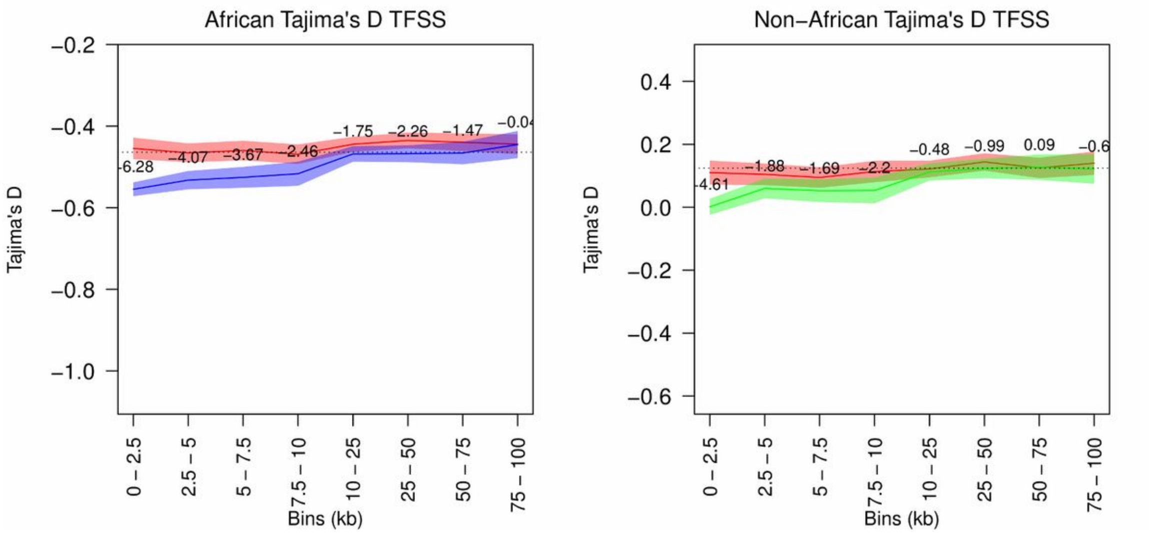

Figure S8: Tajima’s D at varying distance from CDS for non-annotated sequence (red) versus (G) promoters, (H) weak enhancers, and (I) sequence-specific TFs, in African (blue) and non-African (green) populations. Neutral reference is illustrated by dotted line. Shaded areas represent 95% confidence intervals, with Z-scores (non-annotated vs. annotation) shown per bin.

J)

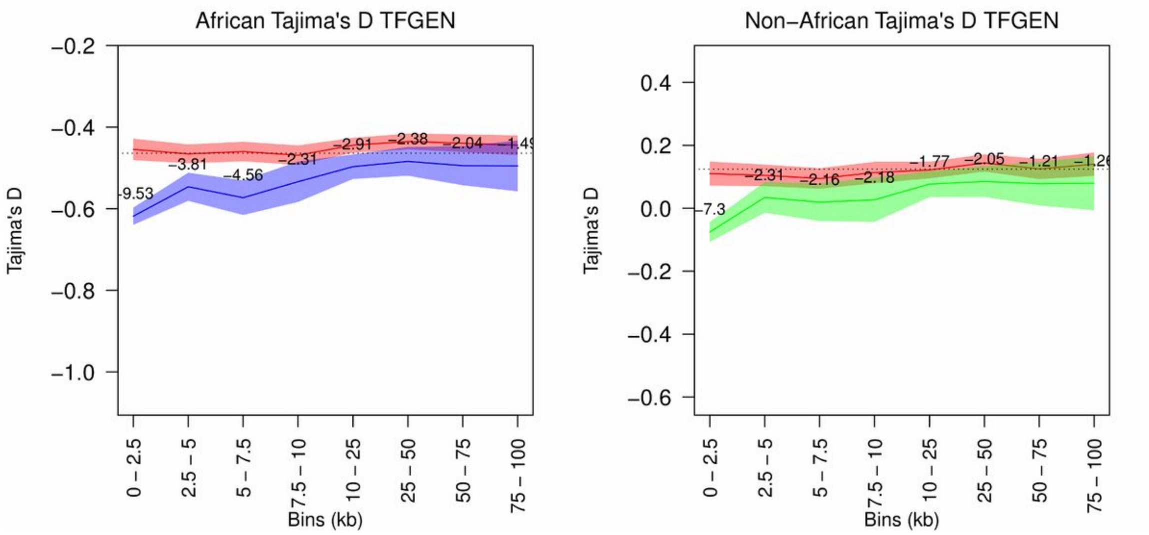

K)

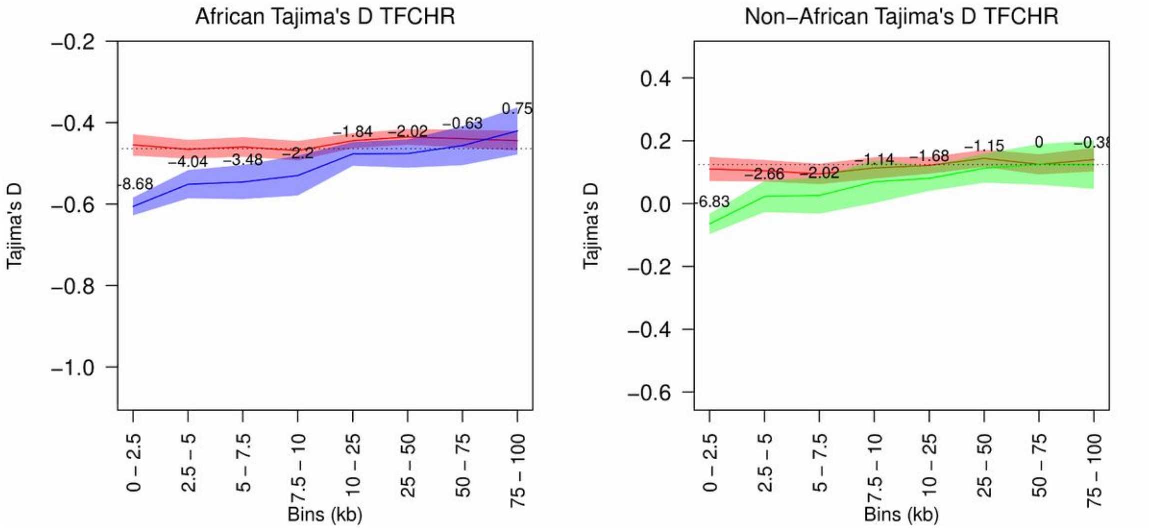

L)

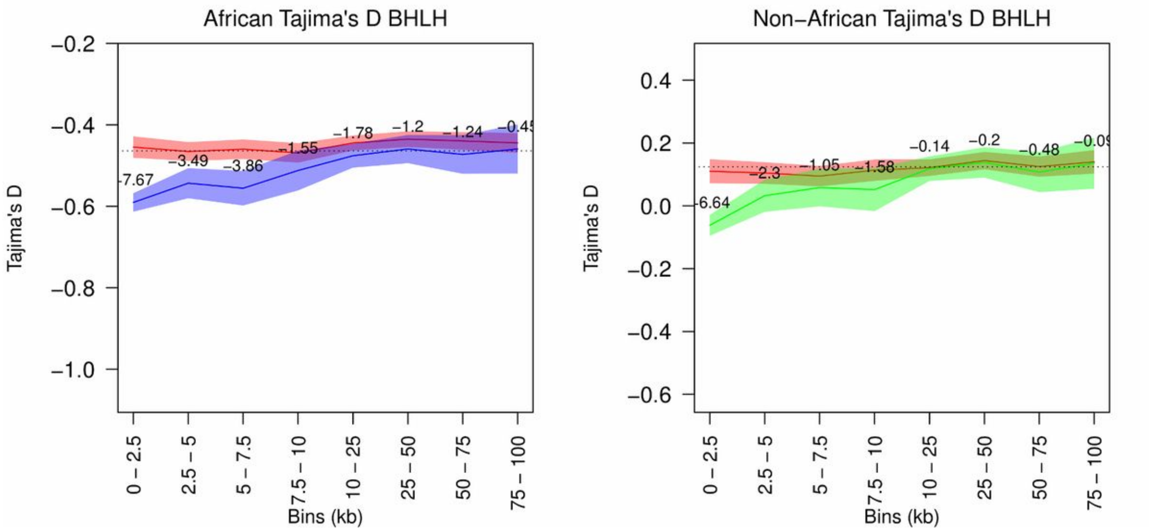

Figure S8: Tajima's D at varying distance from CDS for non-annotated sequence (red) versus (J) general TFs, (K) chromatin-modifying TFs, and (L) bHLH family TFs, in African (blue) and non-African (green) populations. Neutral reference is illustrated by dotted line. Shaded areas represent 95% confidence intervals, with Z-scores (non-annotated vs. annotation) shown per bin.

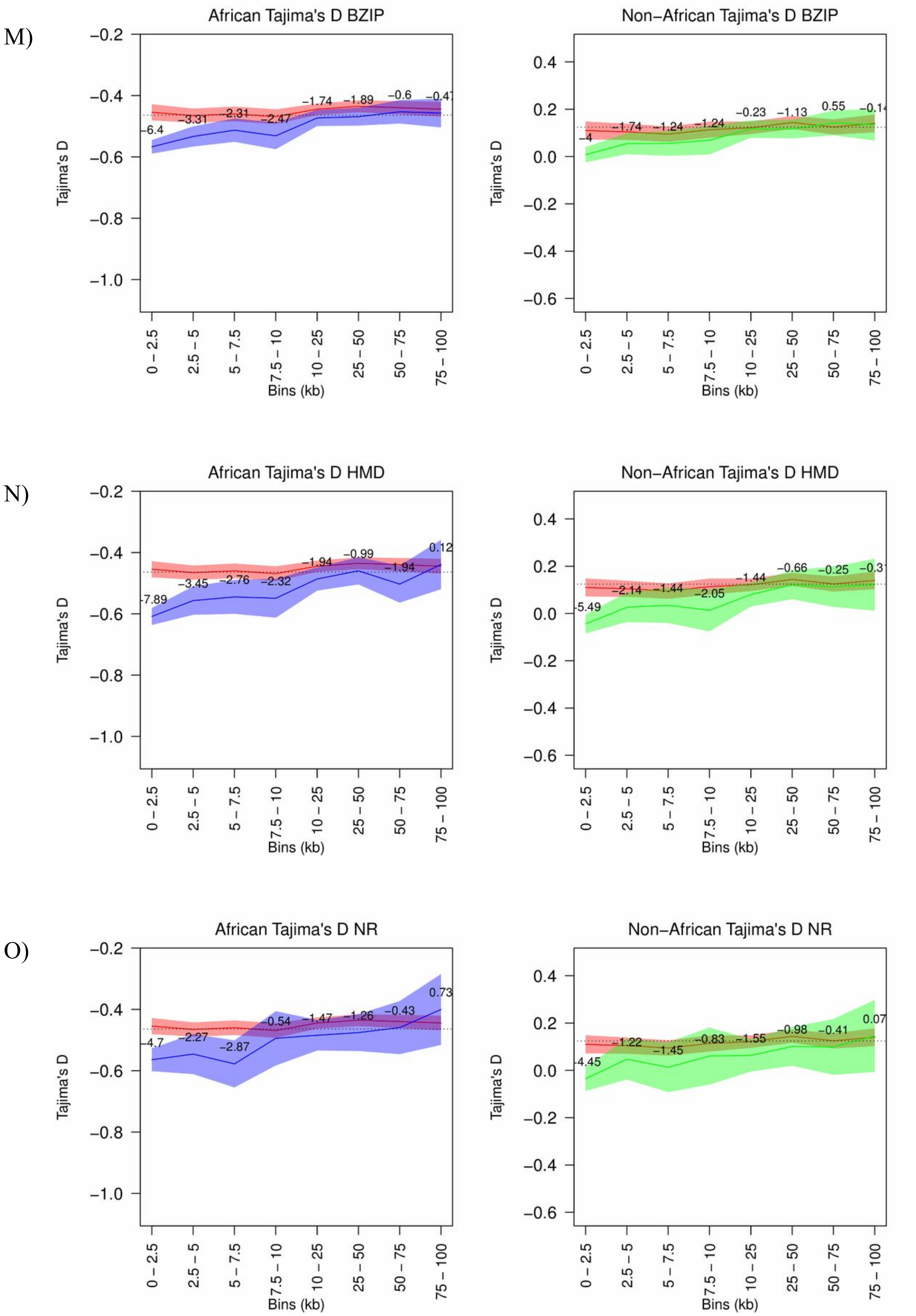

Figure S8: Tajima's D at varying distance from CDS for non-annotated sequence (red) versus (M) bZIP family TFs, (N) HMD family TFs, and (O) NR family TFs, in African (blue) and non-African (green) populations. Neutral reference is illustrated by dotted line. Shaded areas represent 95% confidence intervals, with Z-scores (non-annotated vs. annotation) shown per bin.

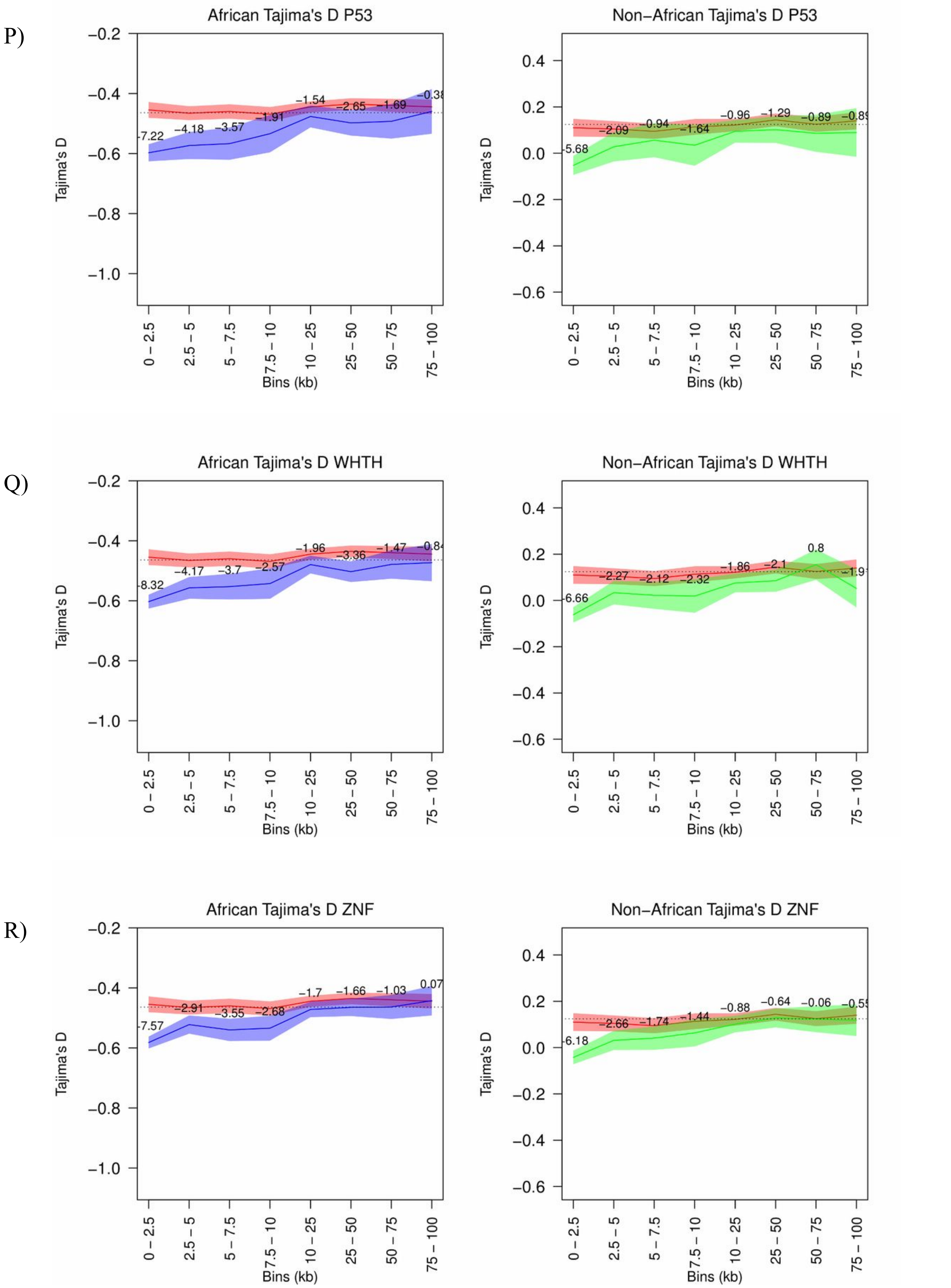

Figure S8: Tajima’s D at varying distance from CDS for non-annotated sequence (red) versus (P) P53 family TFs, (Q) wHTH family TFs, and (R) ZNF family TFs, in African (blue) and non-African (green) populations. Neutral reference is illustrated by dotted line. Shaded areas represent 95% confidence intervals, with Z-scores (non-annotated vs. annotation) shown per bin.

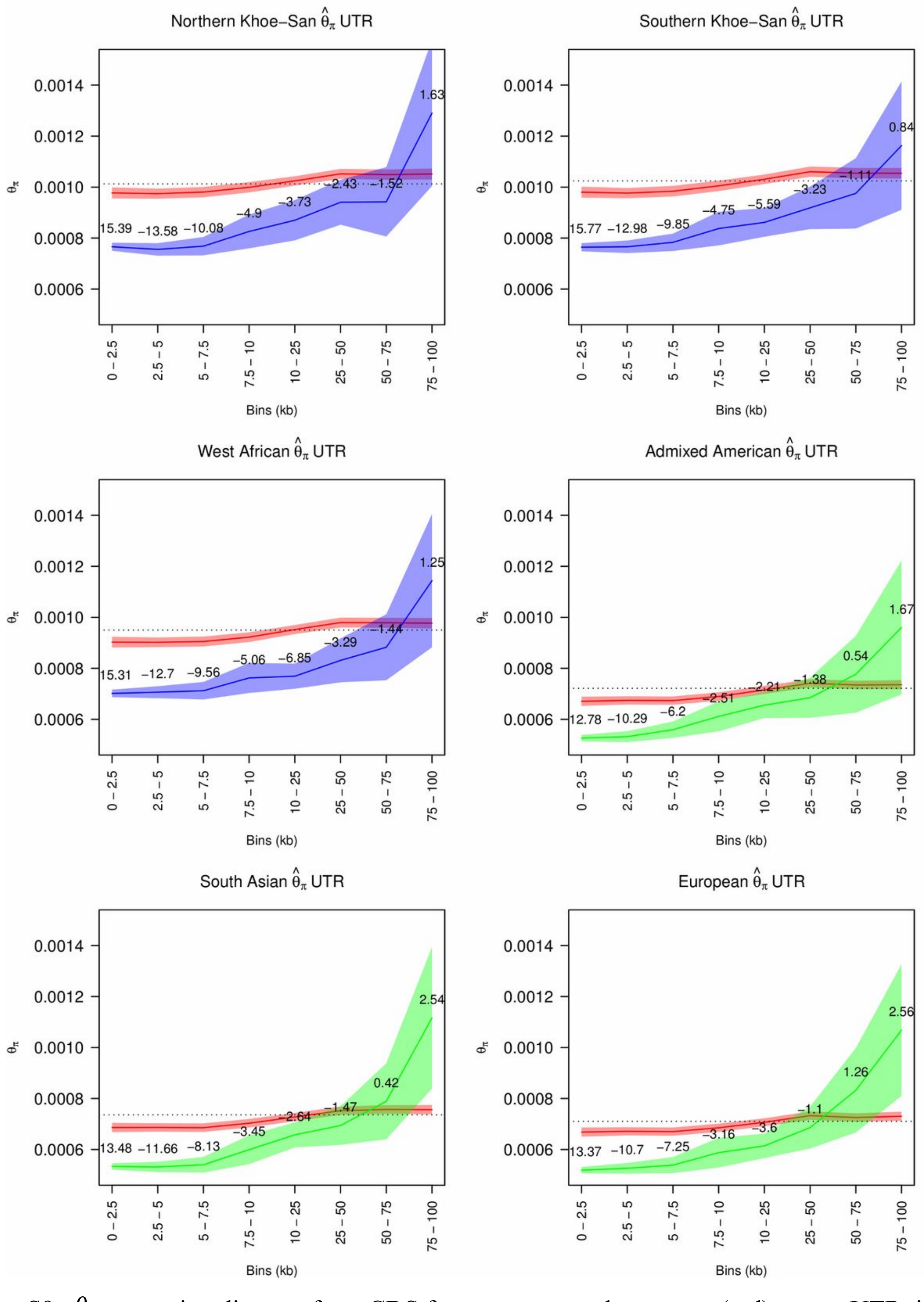

Figure S9: θ_π at varying distance from CDS for non-annotated sequence (red) versus UTR, in the six global pools. Neutral reference is illustrated by dotted line. Shaded areas represent 95% confidence intervals, with Z-scores (non-annotated vs. annotation) shown per bin.

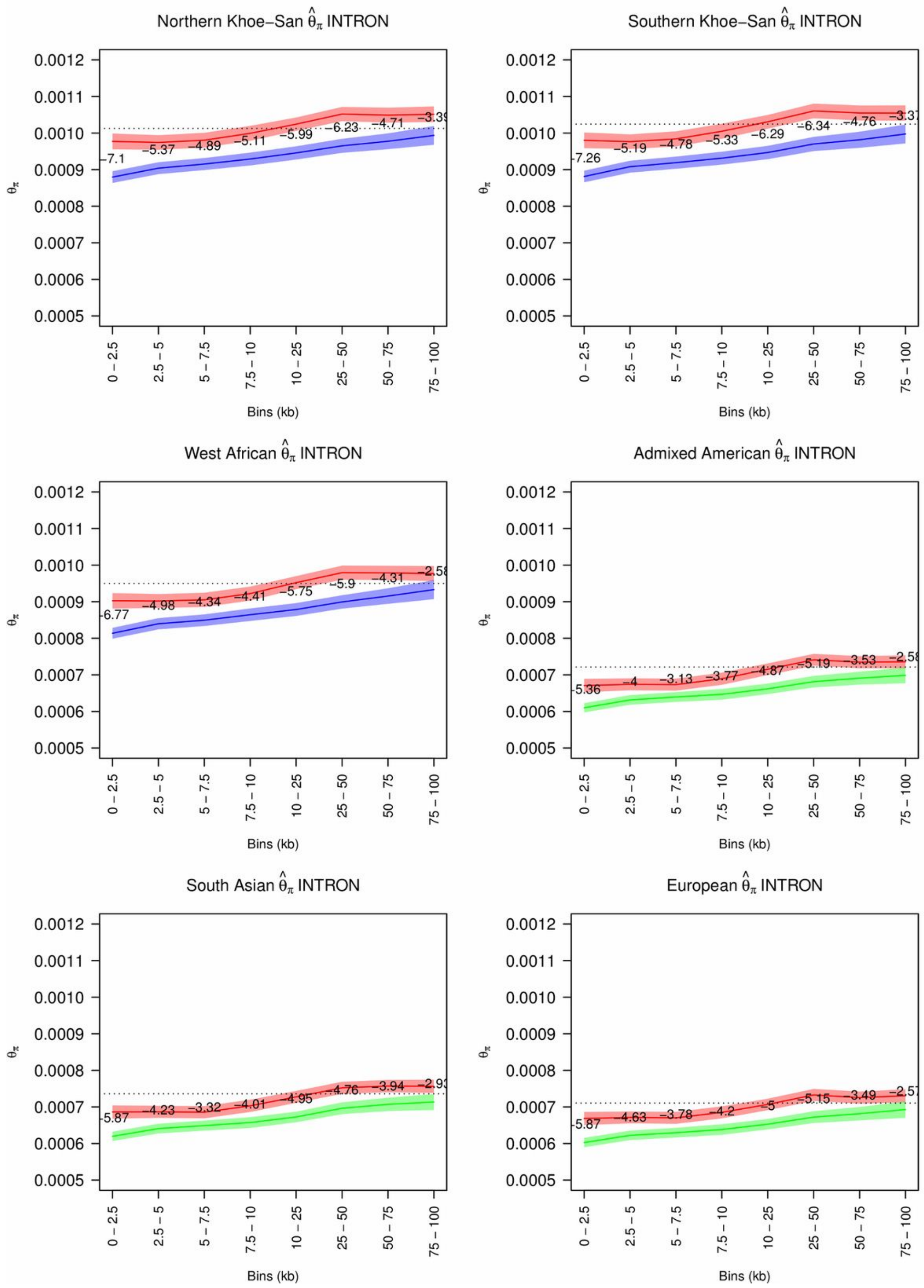

Figure S10: θ_π at varying distance from CDS for non-annotated sequence (red) versus introns, in the six global pools. Neutral reference is illustrated by dotted line. Shaded areas represent 95% confidence intervals, with Z-scores (non-annotated vs. annotation) shown per bin.

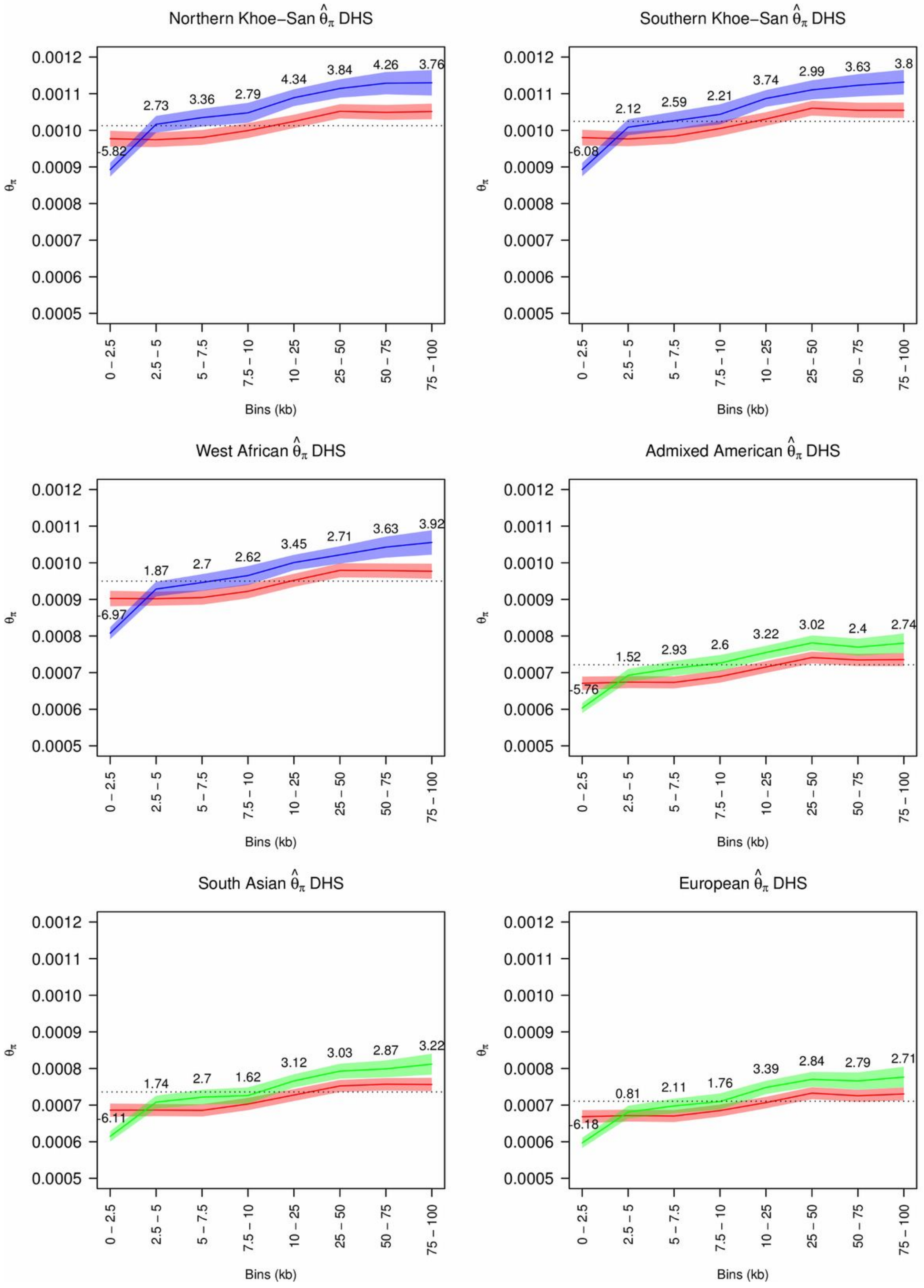

Figure S11: θ_π at varying distance from CDS for non-annotated sequence (red) versus DHS, in the six global pools. Neutral reference is illustrated by dotted line. Shaded areas represent 95% confidence intervals, with Z-scores (non-annotated vs. annotation) shown per bin.

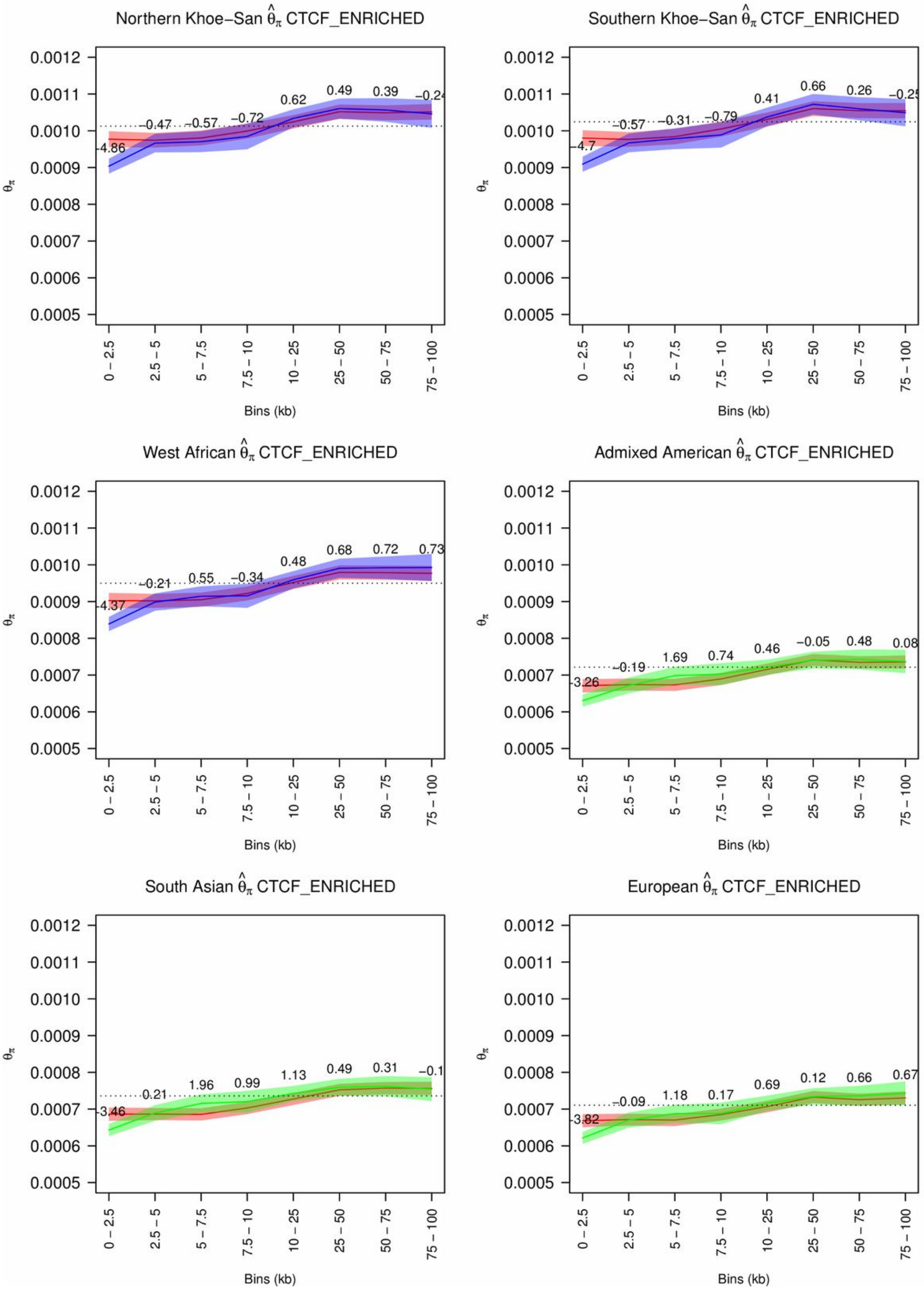

Figure S12: θ_π at varying distance from CDS for non-annotated sequence (red) versus CTCF binding sites, in the six global pools. Neutral reference is illustrated by dotted line. Shaded areas represent 95% confidence intervals, with Z-scores (non-annotated vs. annotation) shown per bin.

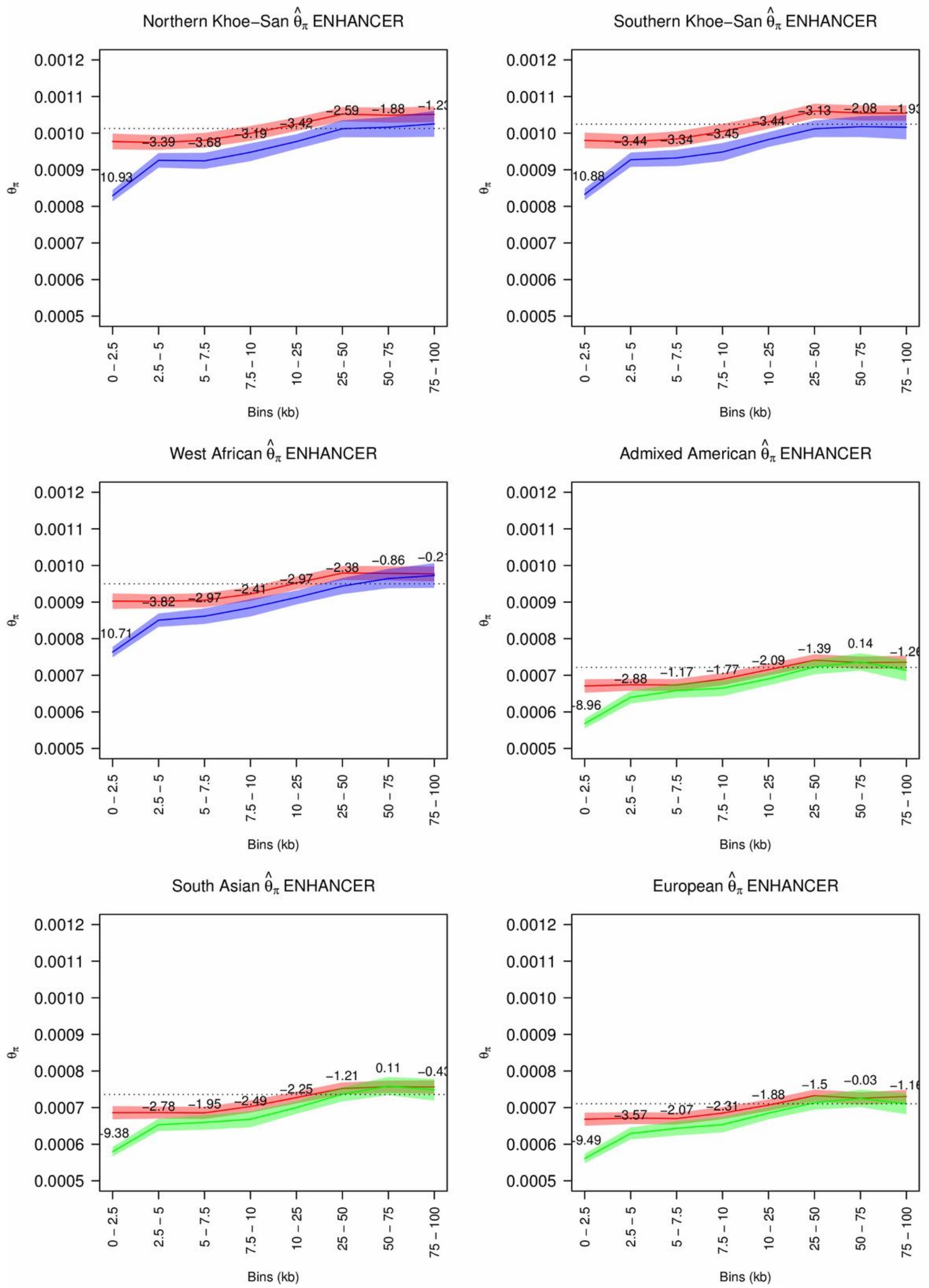

Figure S13: θ_π at varying distance from CDS for non-annotated sequence (red) versus enhancers, in the six global pools. Neutral reference is illustrated by dotted line. Shaded areas represent 95% confidence intervals, with Z-scores (non-annotated vs. annotation) shown per bin.

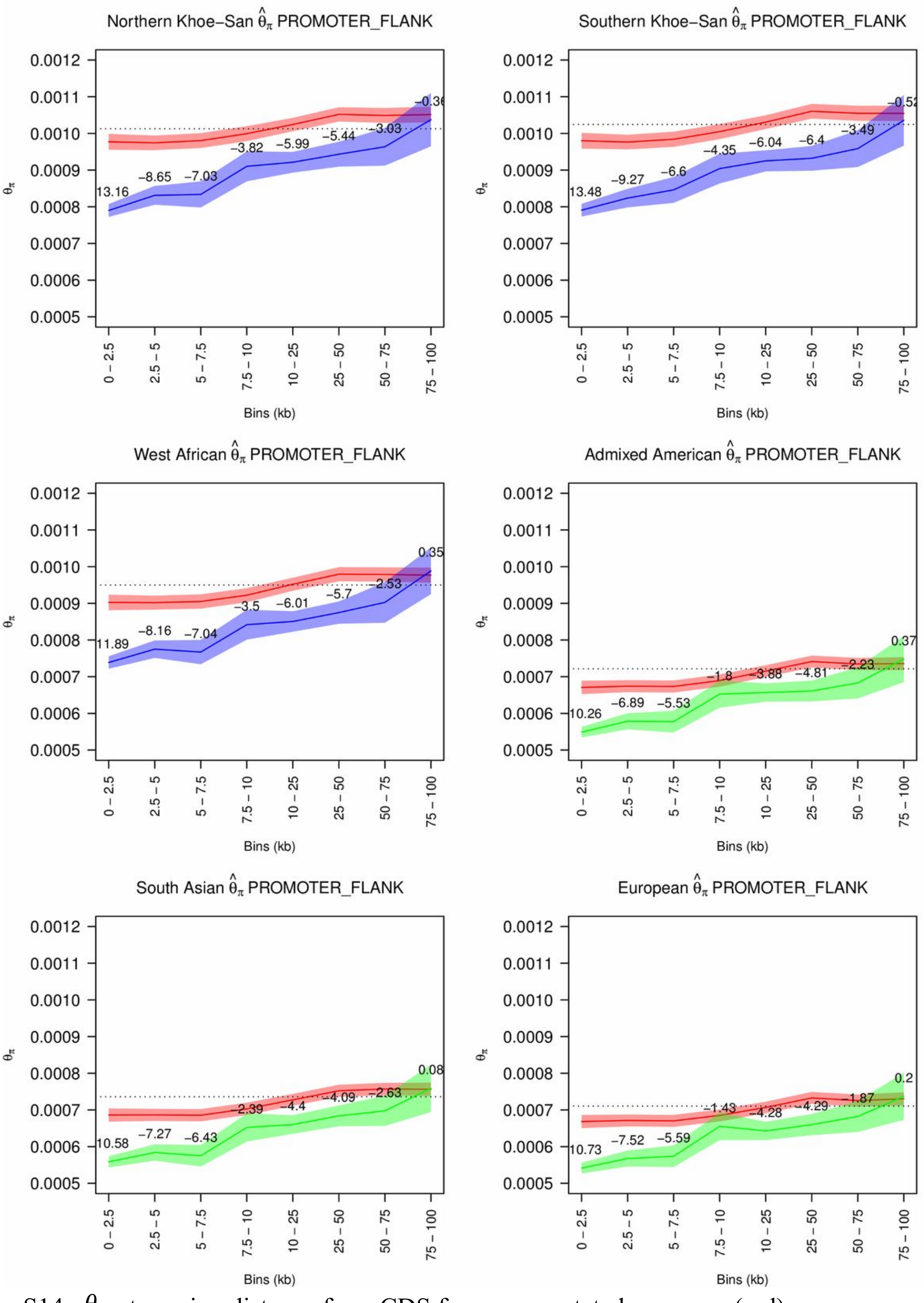

Figure S14: θ_π at varying distance from CDS for non-annotated sequence (red) versus promoter flanking regions, in the six global pools. Neutral reference is illustrated by dotted line. Shaded areas represent 95% confidence intervals, with Z-scores (non-annotated vs. annotation) shown per bin.

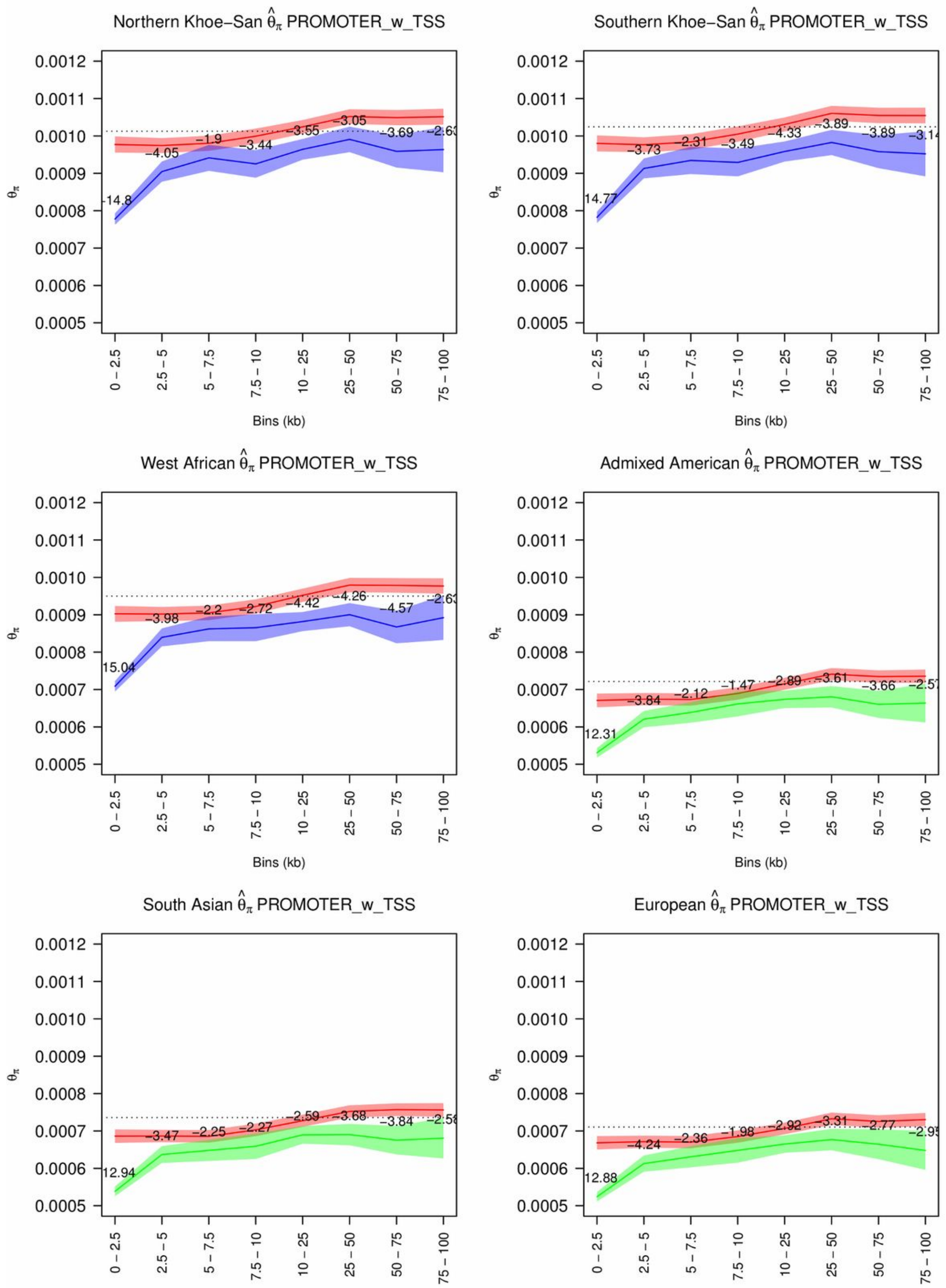

Figure S15: θ_π at varying distance from CDS for non-annotated sequence (red) versus promoters, in the six global pools. Neutral reference is illustrated by dotted line. Shaded areas represent 95% confidence intervals, with Z-scores (non-annotated vs. annotation) shown per bin.

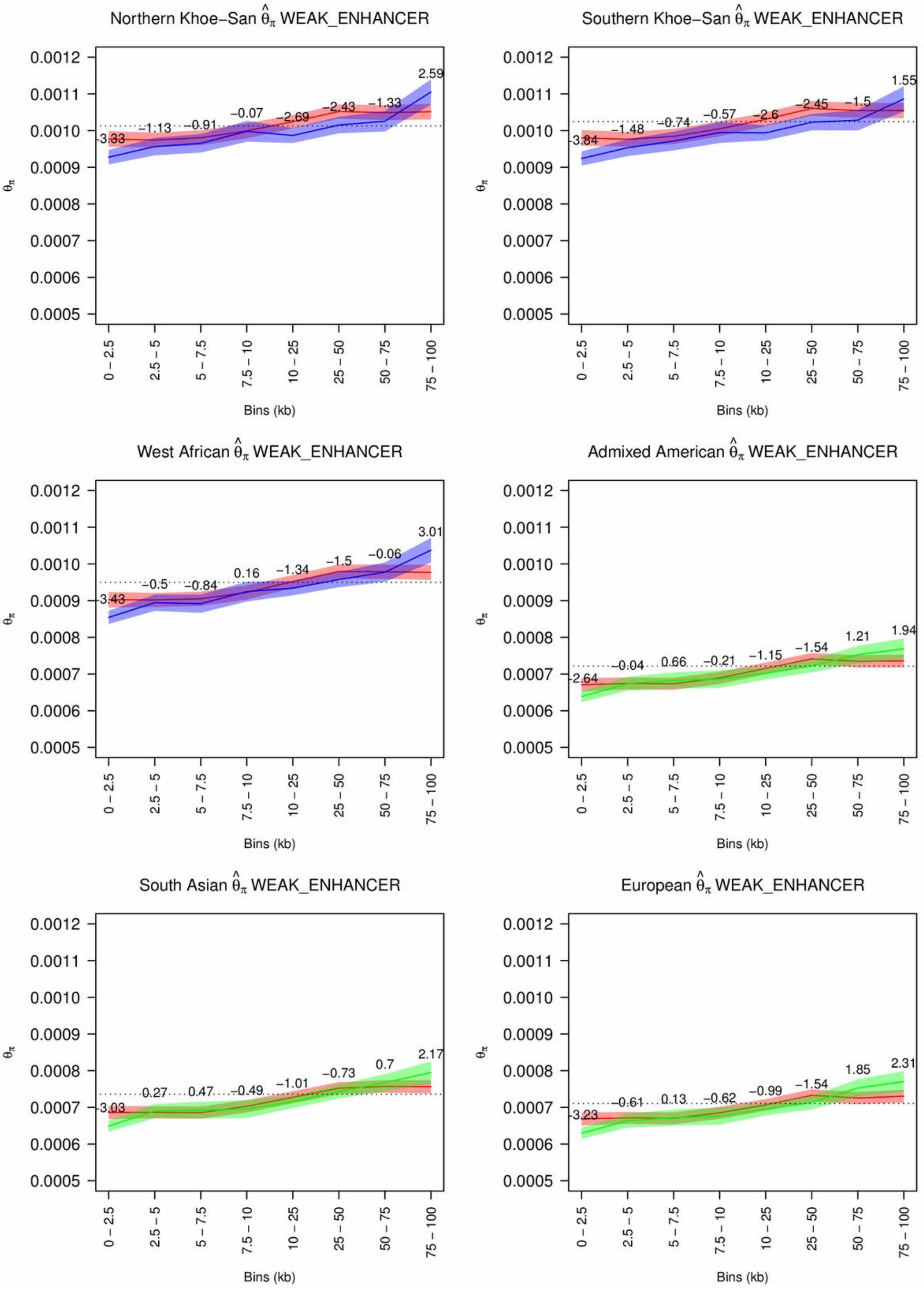

Figure S16: θ_π at varying distance from CDS for non-annotated sequence (red) versus weak enhancers, in the six global pools. Neutral reference is illustrated by dotted line. Shaded areas represent 95% confidence intervals, with Z-scores (non-annotated vs. annotation) shown per bin.

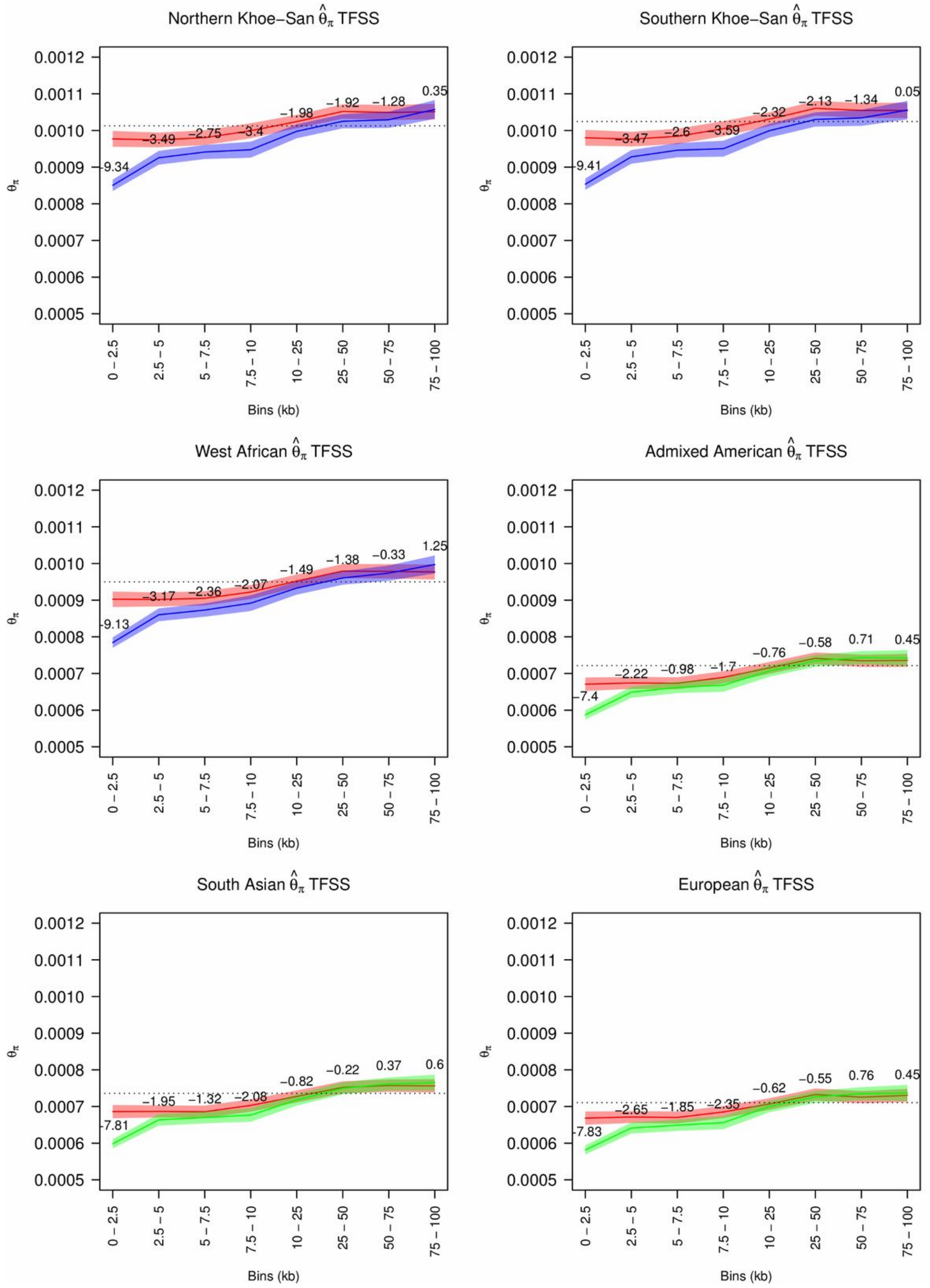

Figure S17: θ_π at varying distance from CDS for non-annotated sequence (red) versus sequence-specific TFs, in the six global pools. Neutral reference is illustrated by dotted line. Shaded areas represent 95% confidence intervals, with Z-scores (non-annotated vs. annotation) shown per bin.

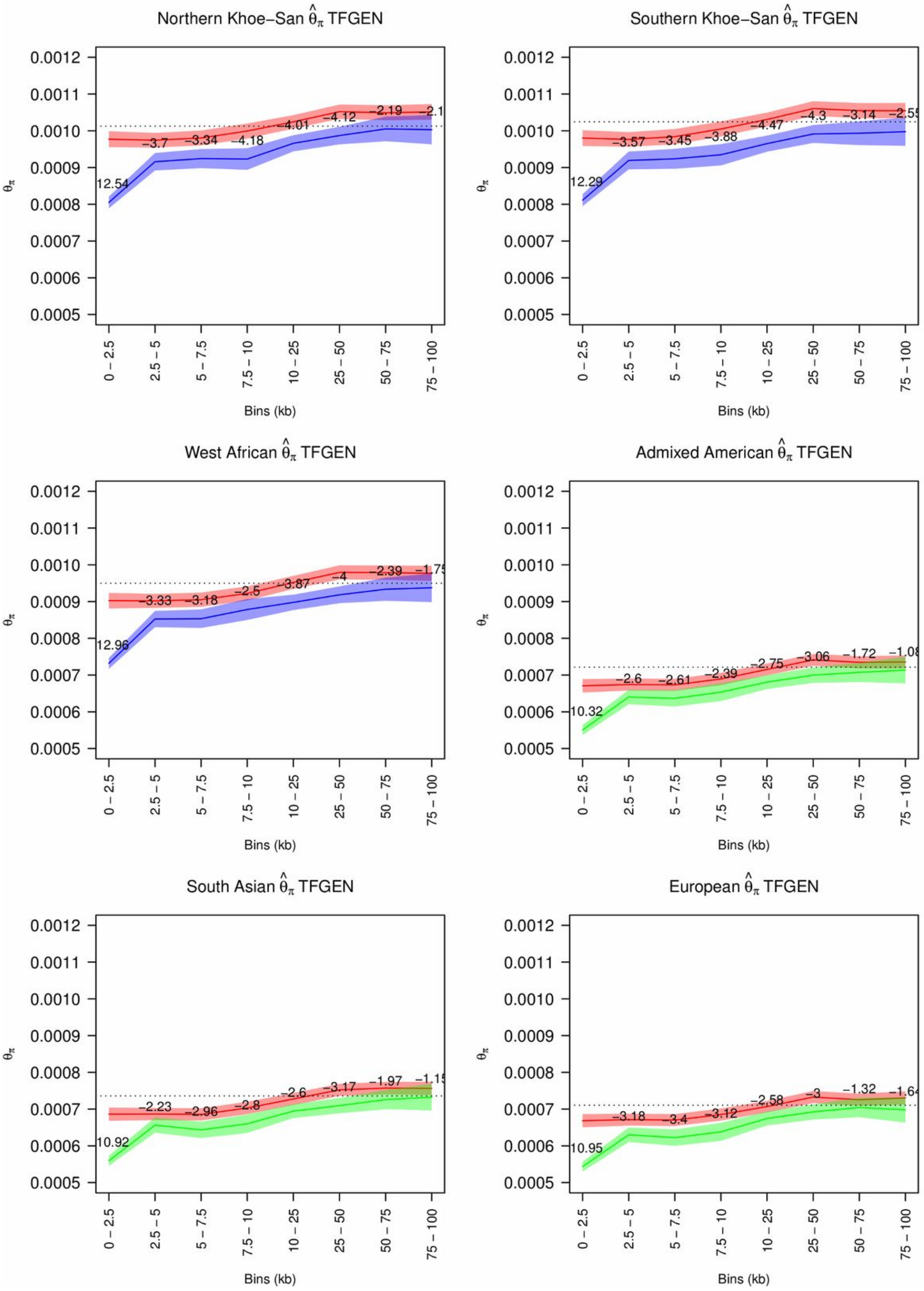

Figure S18: θ_π at varying distance from CDS for non-annotated sequence (red) versus general TFs, in the six global pools. Neutral reference is illustrated by dotted line. Shaded areas represent 95% confidence intervals, with Z-scores (non-annotated vs. annotation) shown per bin.

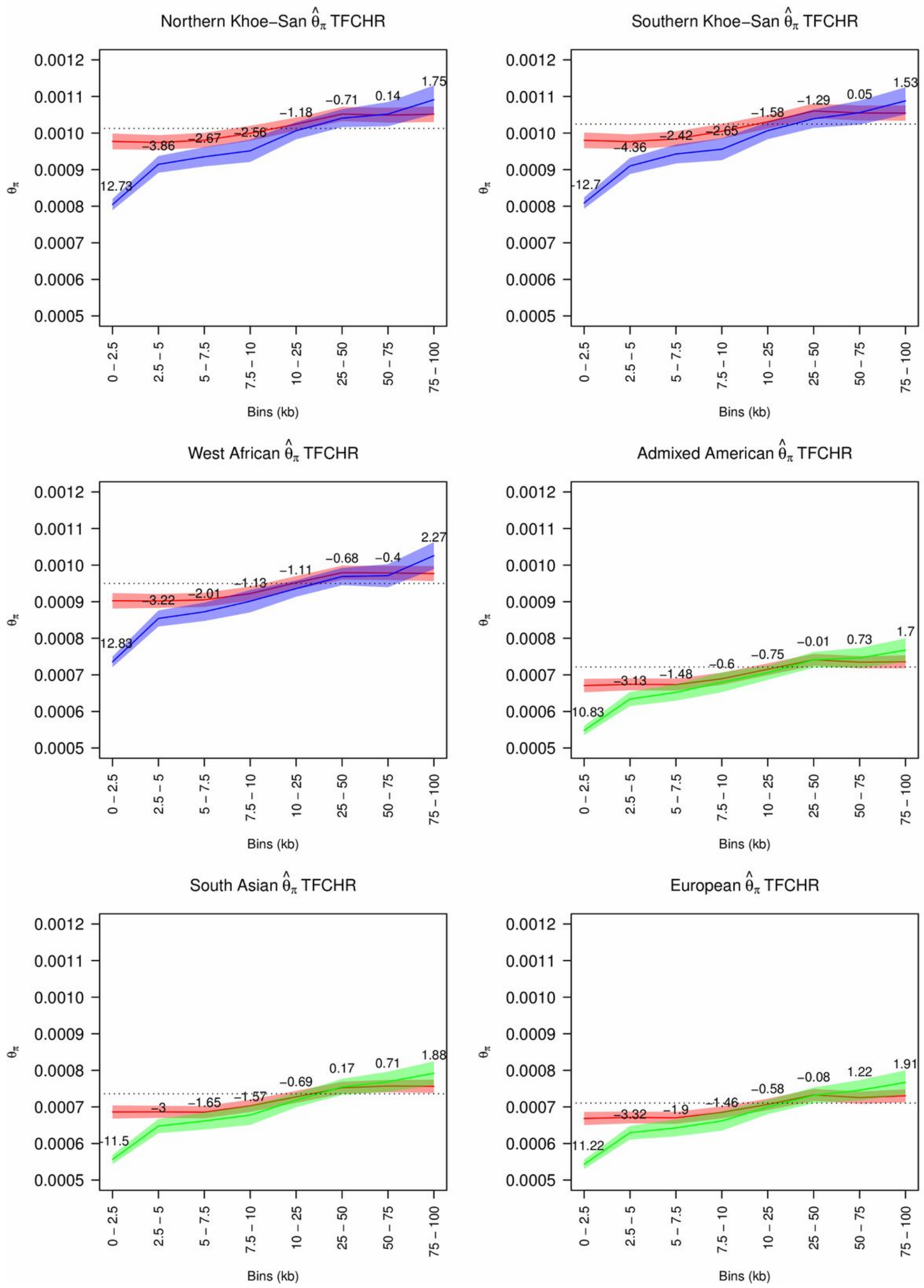

Figure S19: θ_π at varying distance from CDS for non-annotated sequence (red) versus chromatin-modifying TFs, in the six global pools. Neutral reference is illustrated by dotted line. Shaded areas represent 95% confidence intervals, with Z-scores (non-annotated vs. annotation) shown per bin.

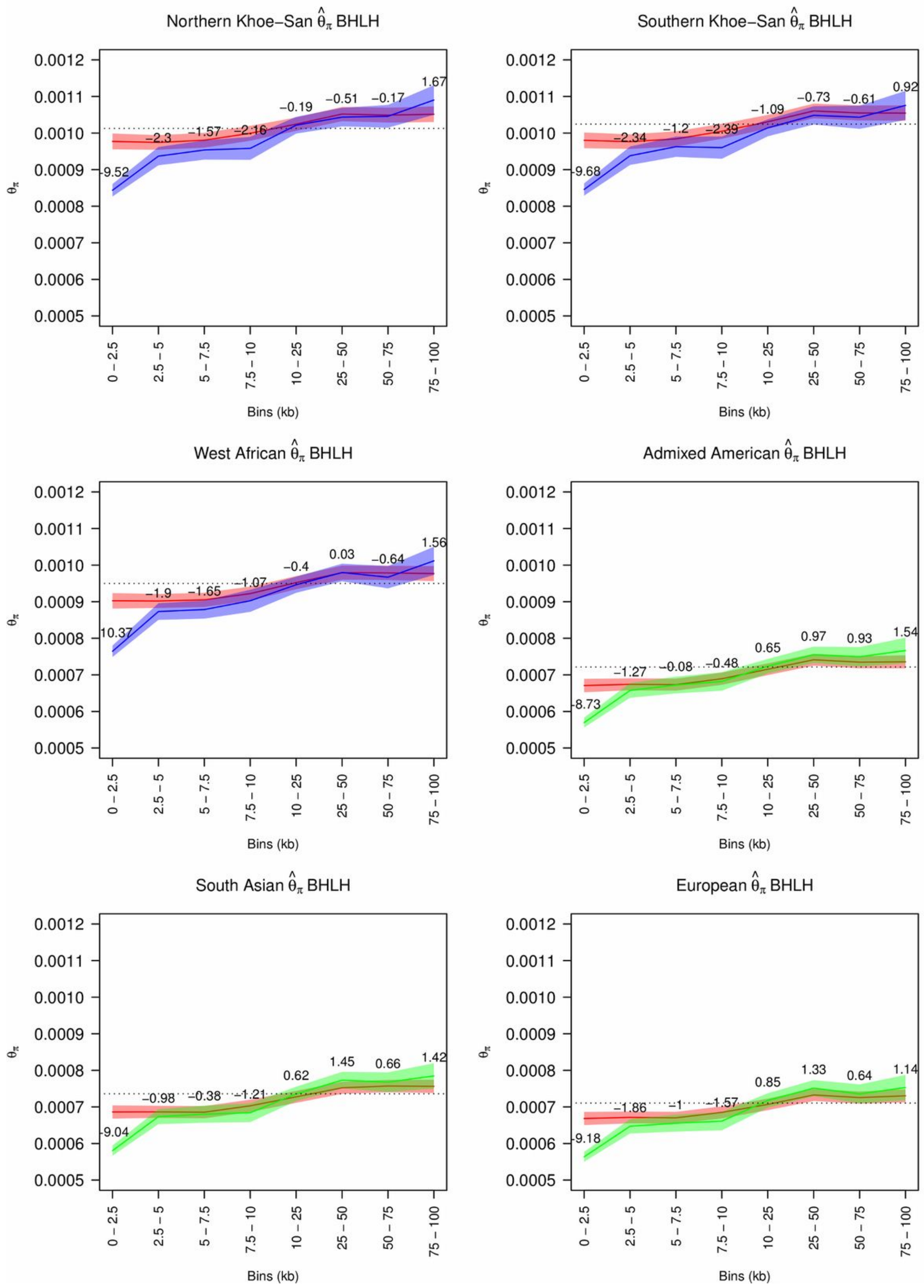

Figure S20: θ_π at varying distance from CDS for non-annotated sequence (red) versus bHLH family TFs, in the six global pools. Neutral reference is illustrated by dotted line. Shaded areas represent 95% confidence intervals, with Z-scores (non-annotated vs. annotation) shown per bin.

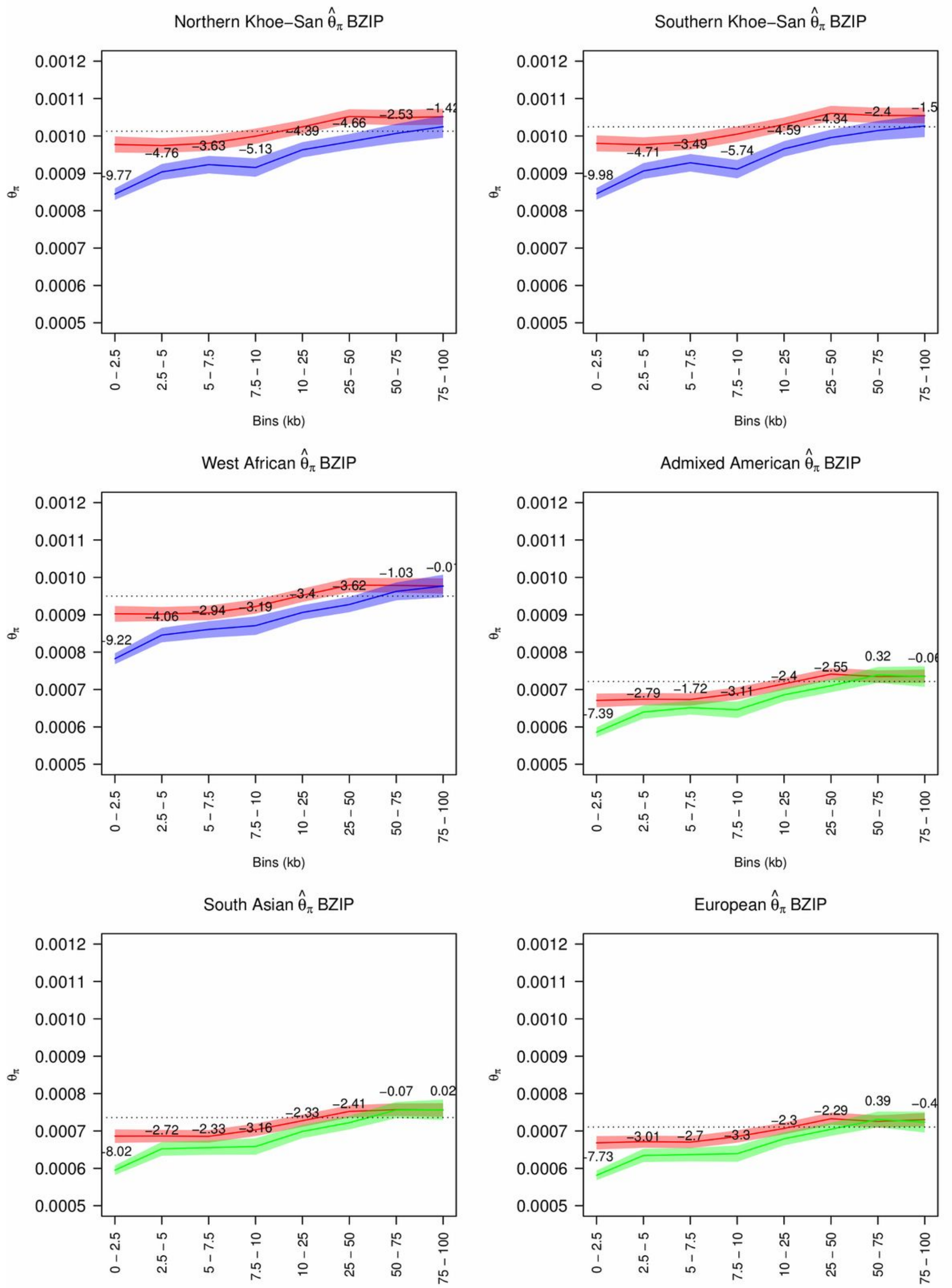

Figure S21: θ_π at varying distance from CDS for non-annotated sequence (red) versus bZIP family TFs, in the six global pools. Neutral reference is illustrated by dotted line. Shaded areas represent 95% confidence intervals, with Z-scores (non-annotated vs. annotation) shown per bin.

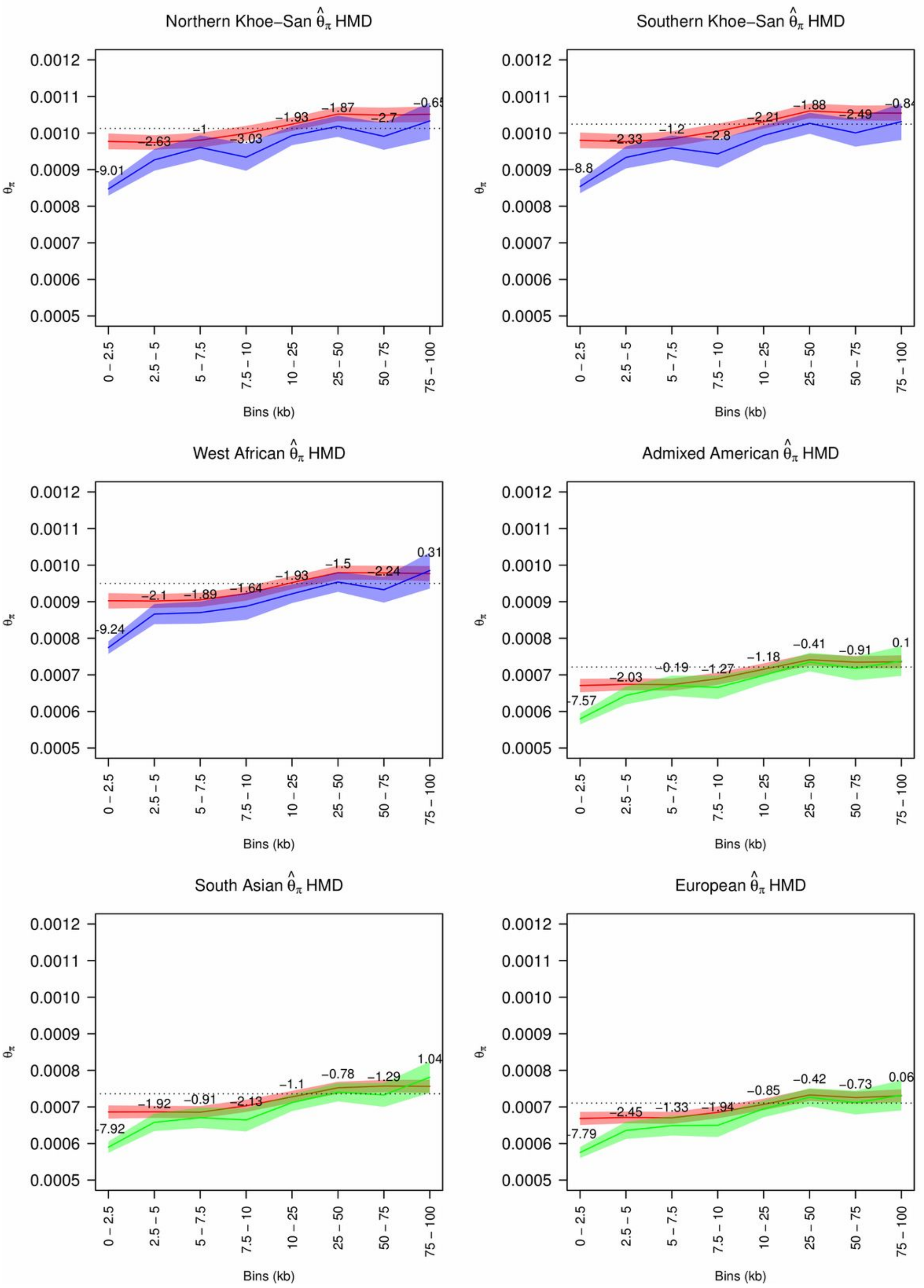

Figure S22: θ_π at varying distance from CDS for non-annotated sequence (red) versus HMD family TFs, in the six global pools. Neutral reference is illustrated by dotted line. Shaded areas represent 95% confidence intervals, with Z-scores (non-annotated vs. annotation) shown per bin.

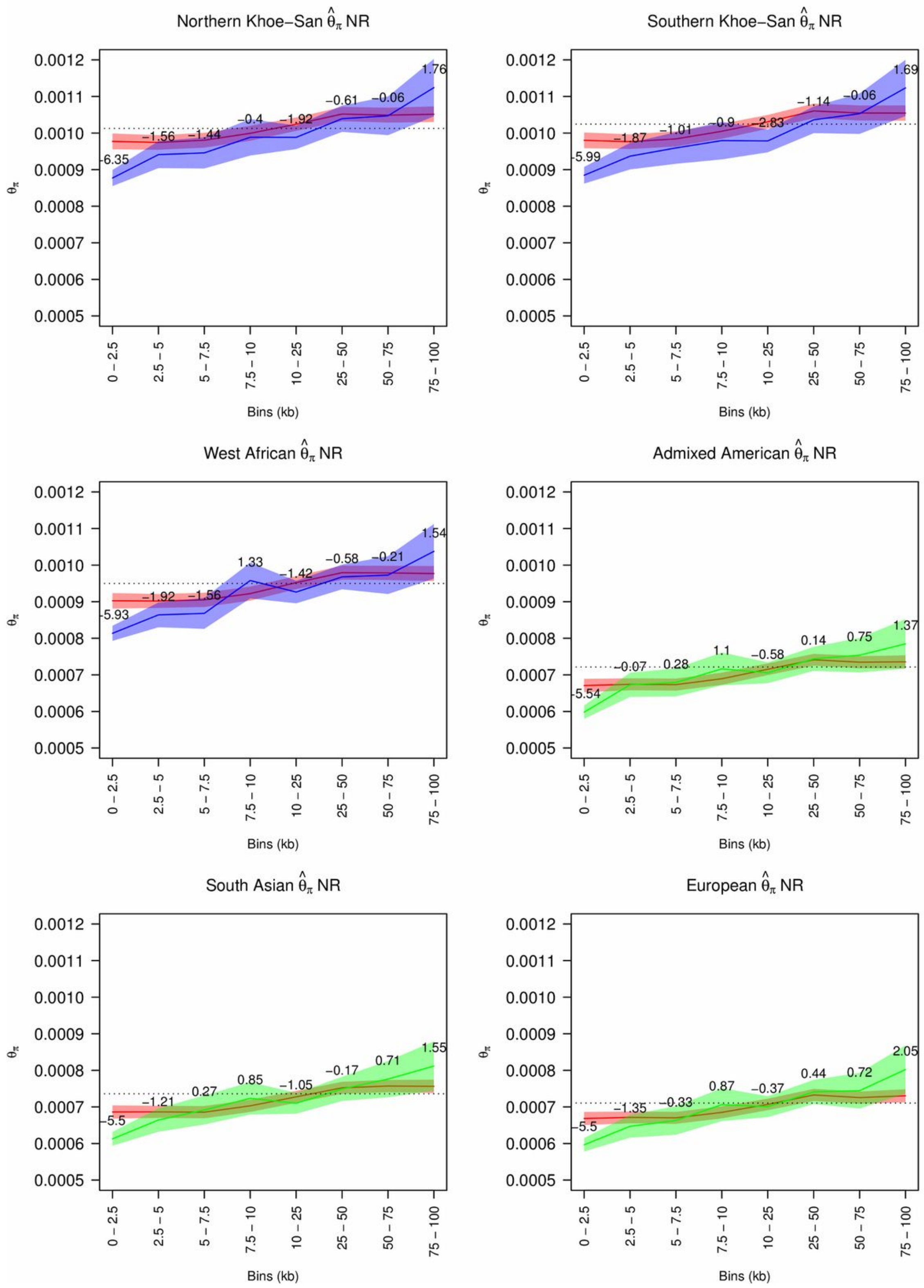

Figure S23: θ_π at varying distance from CDS for non-annotated sequence (red) versus NR family TFs, in the six global pools. Neutral reference is illustrated by dotted line. Shaded areas represent 95% confidence intervals, with Z-scores (non-annotated vs. annotation) shown per bin.

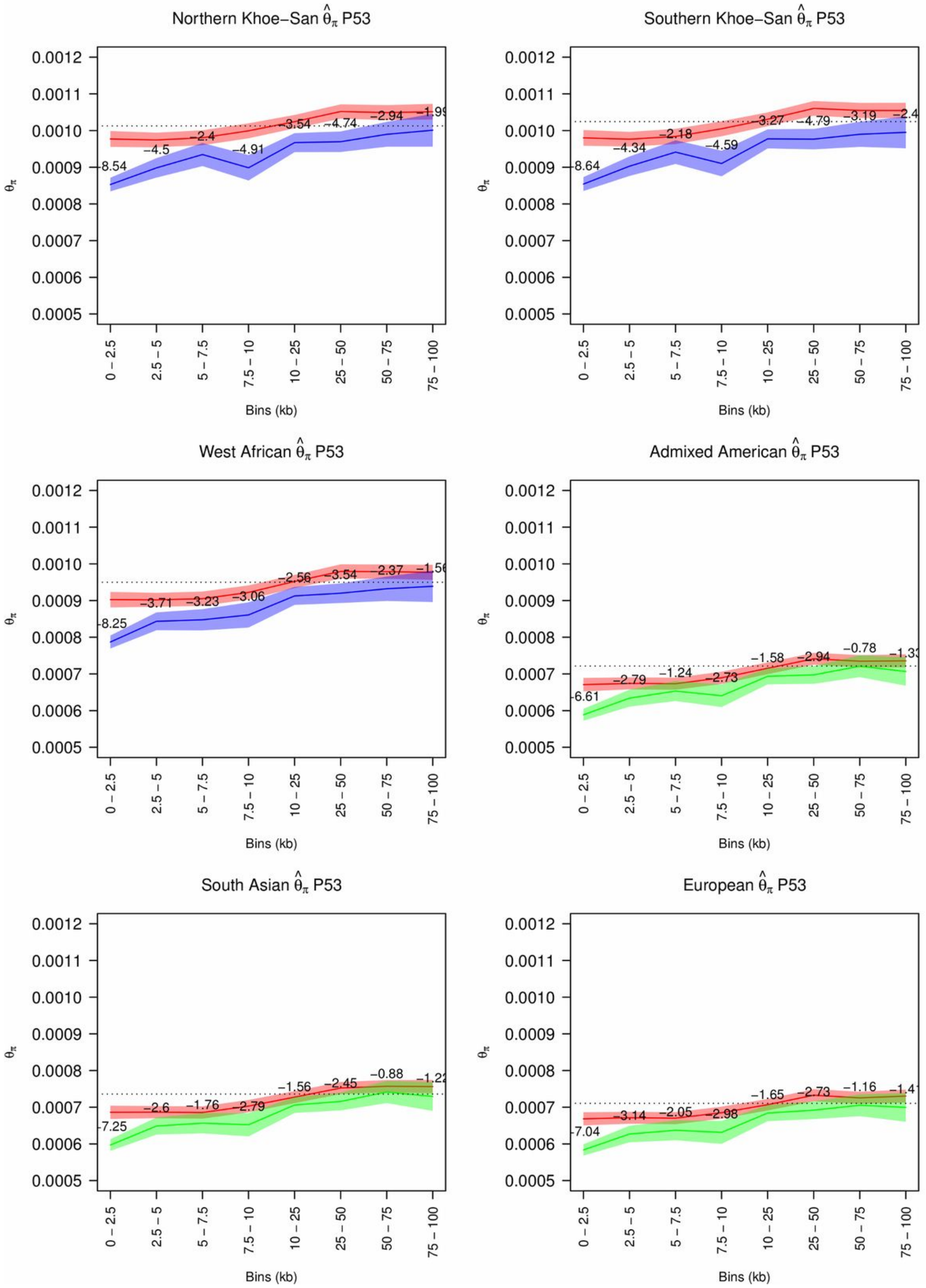

Figure S24: θ_π at varying distance from CDS for non-annotated sequence (red) versus P53 family TFs, in the six global pools. Neutral reference is illustrated by dotted line. Shaded areas represent 95% confidence intervals, with Z-scores (non-annotated vs. annotation) shown per bin.

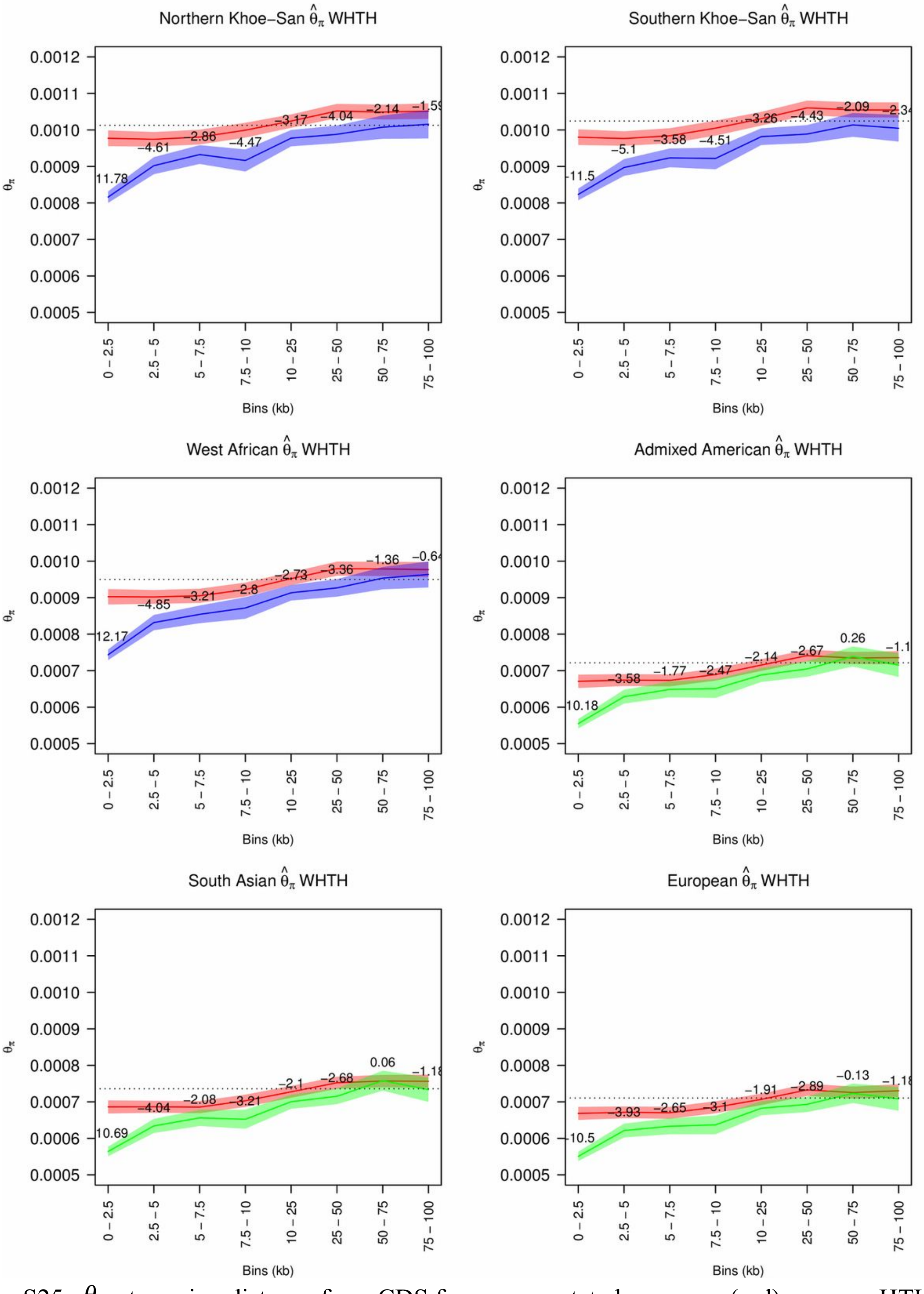

Figure S25: θ_π at varying distance from CDS for non-annotated sequence (red) versus wHTH family TFs, in the six global pools. Neutral reference is illustrated by dotted line. Shaded areas represent 95% confidence intervals, with Z-scores (non-annotated vs. annotation) shown per bin.

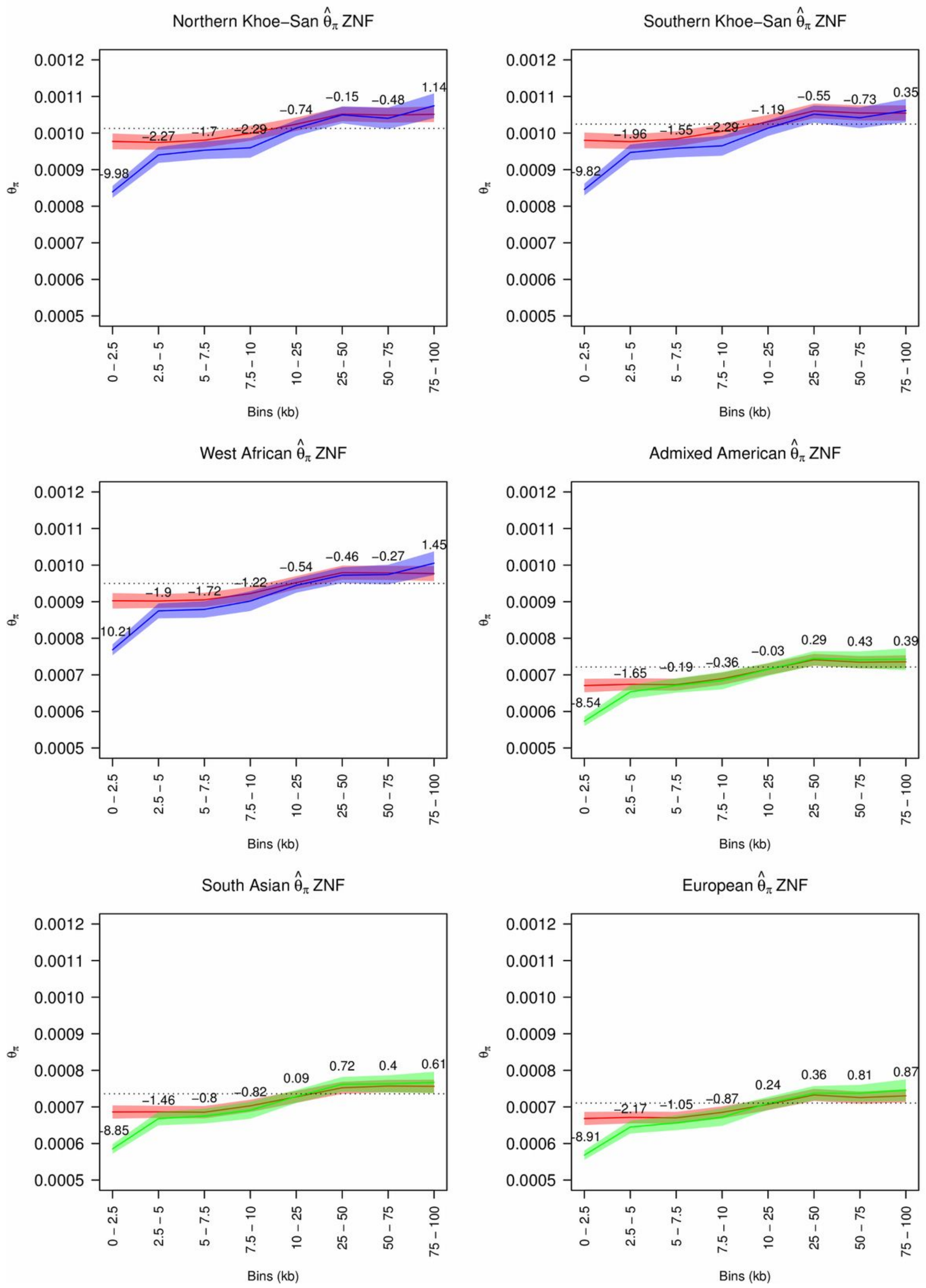

Figure S26: θ_π at varying distance from CDS for non-annotated sequence (red) versus ZNF family TFs, in the six global pools. Neutral reference is illustrated by dotted line. Shaded areas represent 95% confidence intervals, with Z-scores (non-annotated vs. annotation) shown per bin.

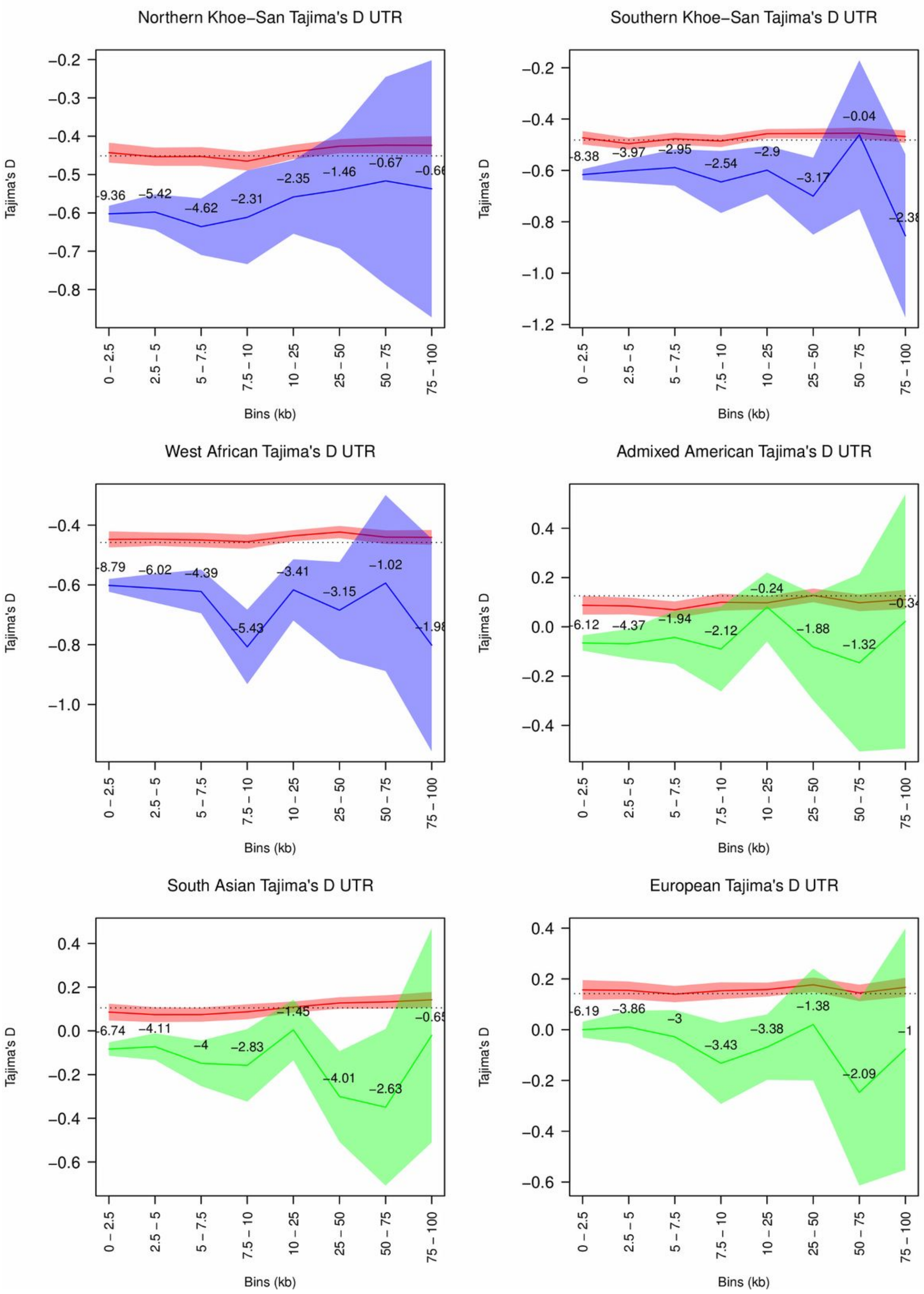

Figure S27: Tajima’s D at varying distance from CDS for non-annotated sequence (red) versus UTR, in the six global pools. Neutral reference is illustrated by dotted line. Shaded areas represent 95% confidence intervals, with Z-scores (non-annotated vs. annotation) shown per bin.

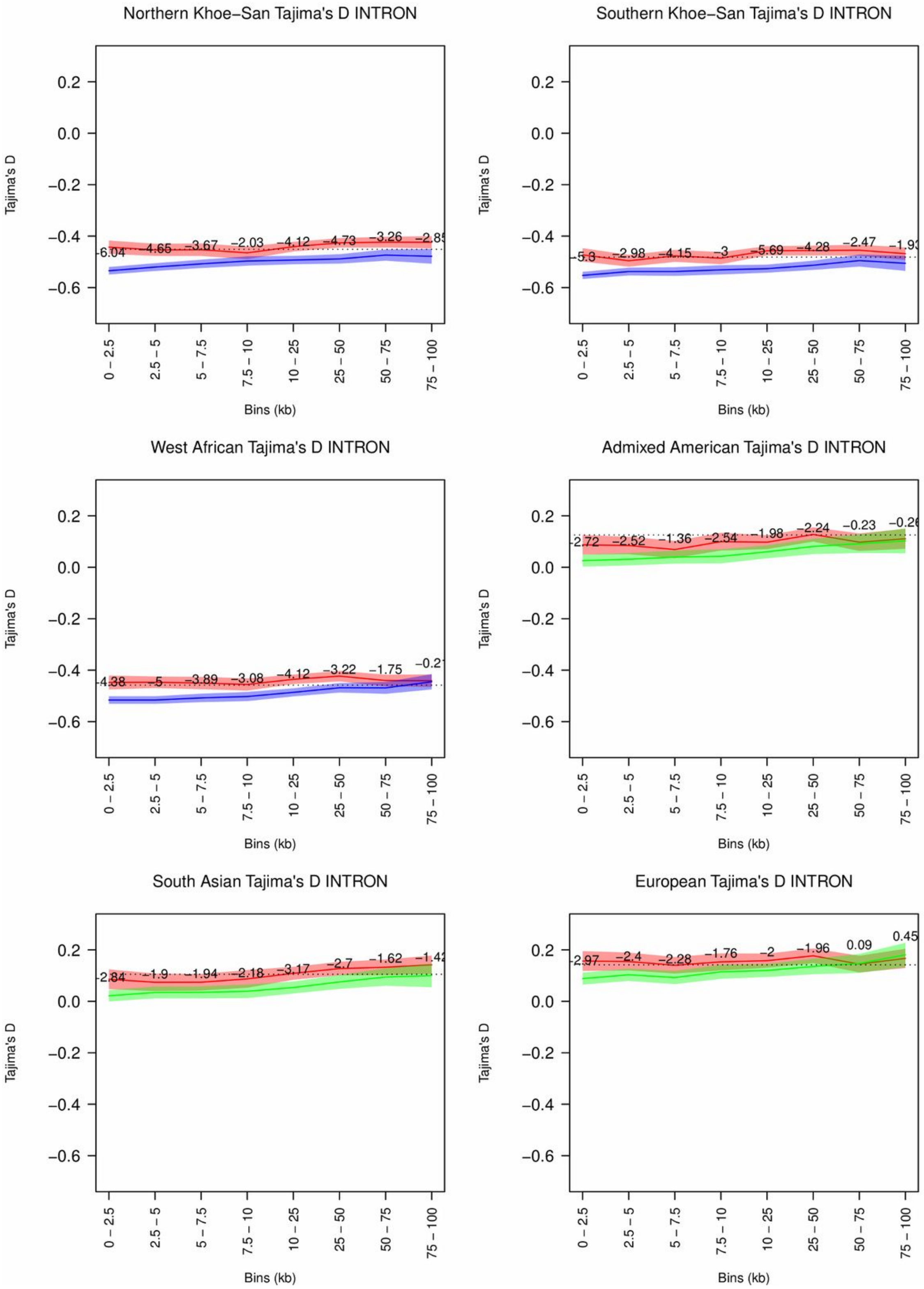

Figure S28: Tajima’s D at varying distance from CDS for non-annotated sequence (red) versus introns, in the six global pools. Neutral reference is illustrated by dotted line. Shaded areas represent 95% confidence intervals, with Z-scores (non-annotated vs. annotation) shown per bin.

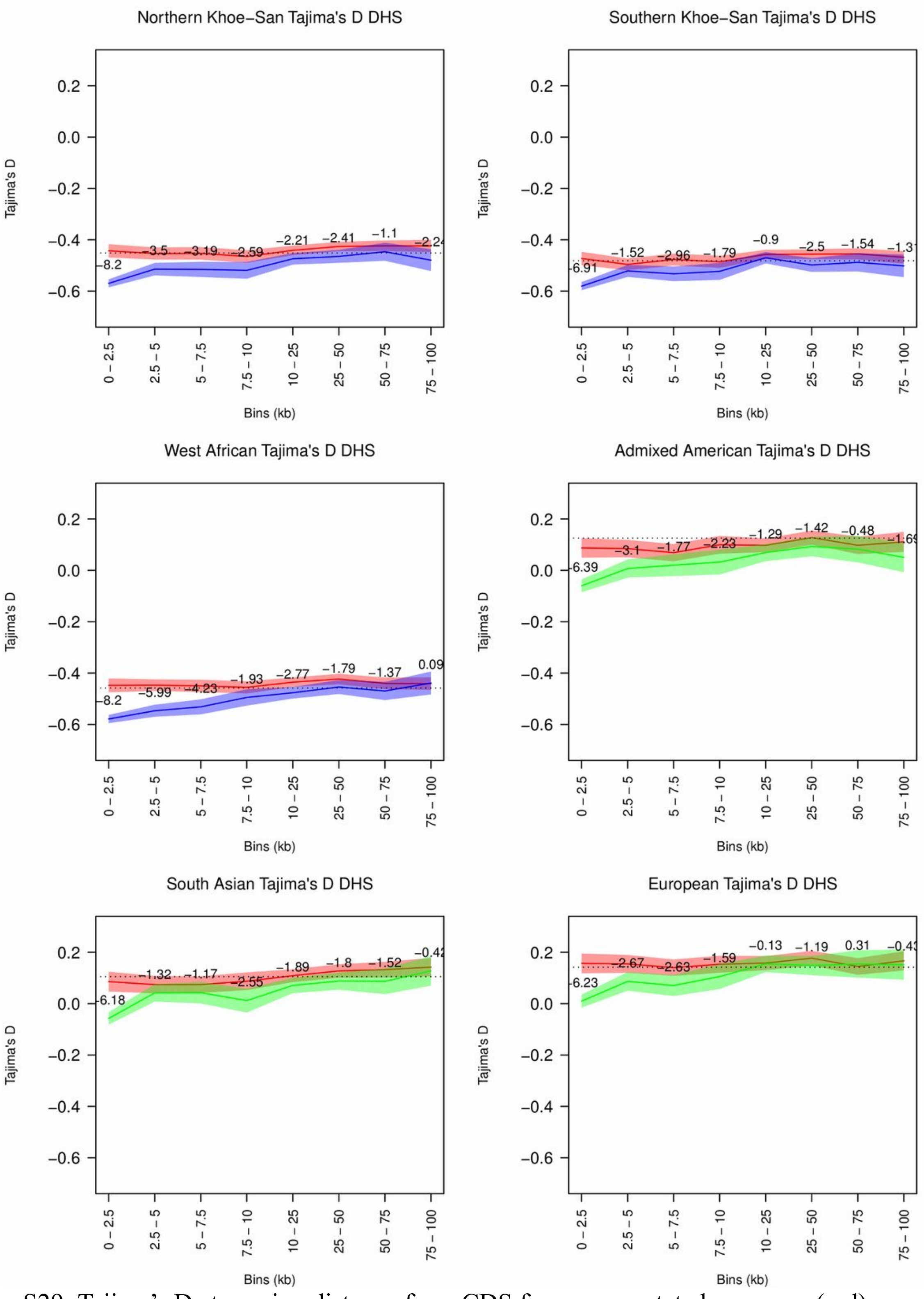

Figure S29: Tajima’s D at varying distance from CDS for non-annotated sequence (red) versus DHS, in the six global pools. Neutral reference is illustrated by dotted line. Shaded areas represent 95% confidence intervals, with Z-scores (non-annotated vs. annotation) shown per bin.

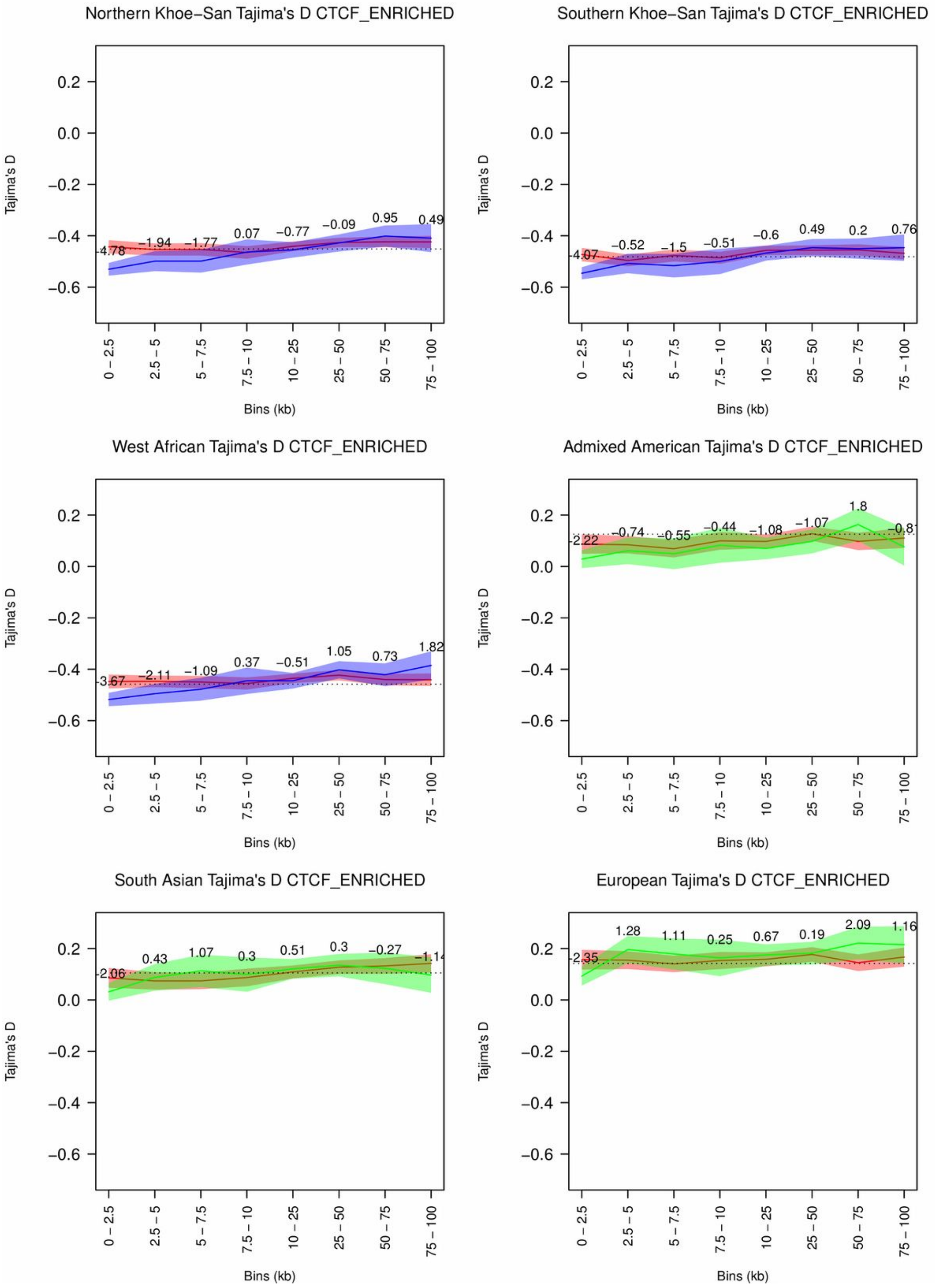

Figure S30: Tajima’s D at varying distance from CDS for non-annotated sequence (red) versus CTCF binding sites, in the six global pools. Neutral reference is illustrated by dotted line. Shaded areas represent 95% confidence intervals, with Z-scores (non-annotated vs. annotation) shown per bin.

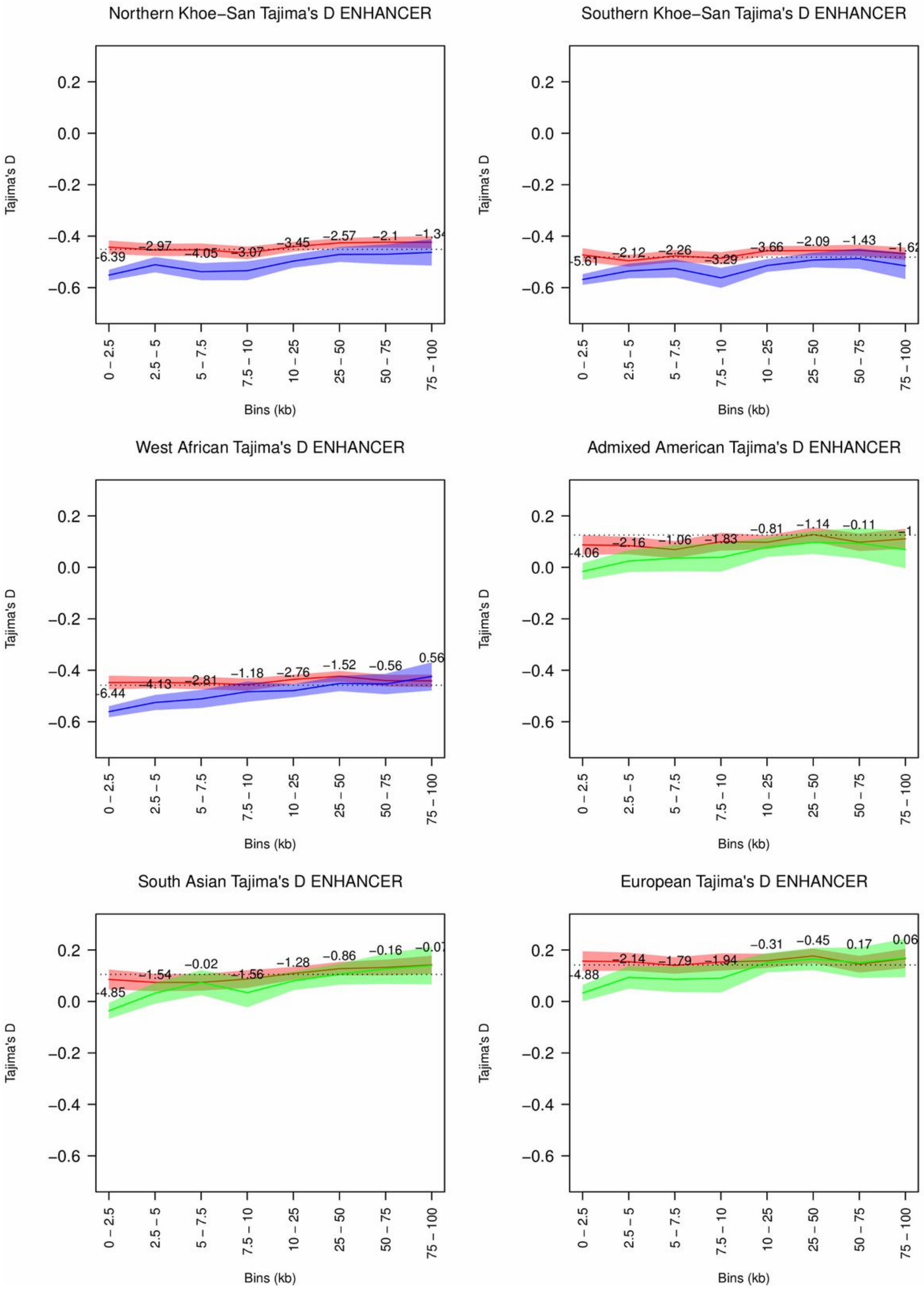

Figure S31: Tajima’s D at varying distance from CDS for non-annotated sequence (red) versus enhancers, in the six global pools. Neutral reference is illustrated by dotted line. Shaded areas represent 95% confidence intervals, with Z-scores (non-annotated vs. annotation) shown per bin.

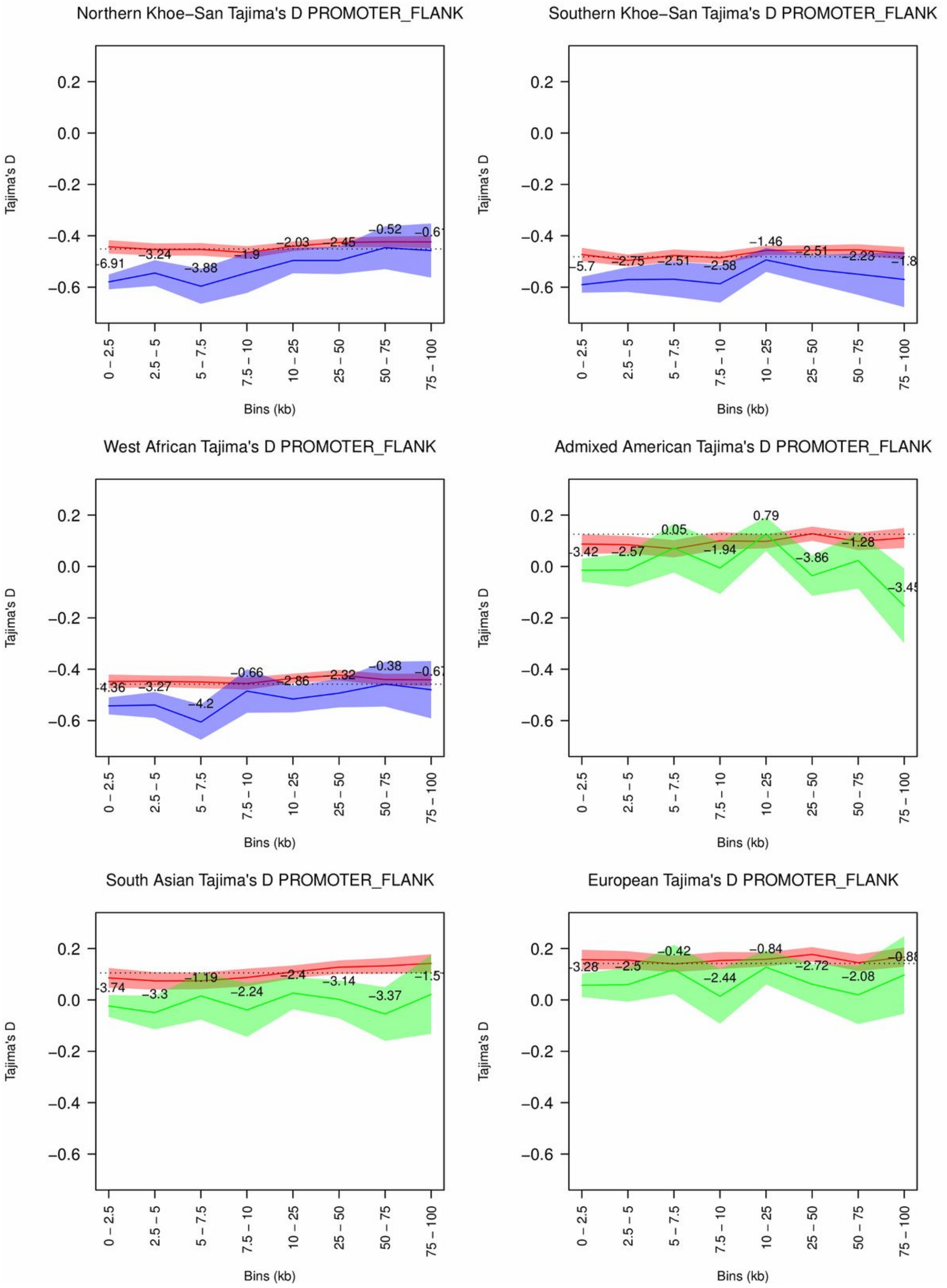

Figure S32: Tajima’s D at varying distance from CDS for non-annotated sequence (red) versus promoter flanking regions, in the six global pools. Neutral reference is illustrated by dotted line. Shaded areas represent 95% confidence intervals, with Z-scores (non-annotated vs. annotation) shown per bin.

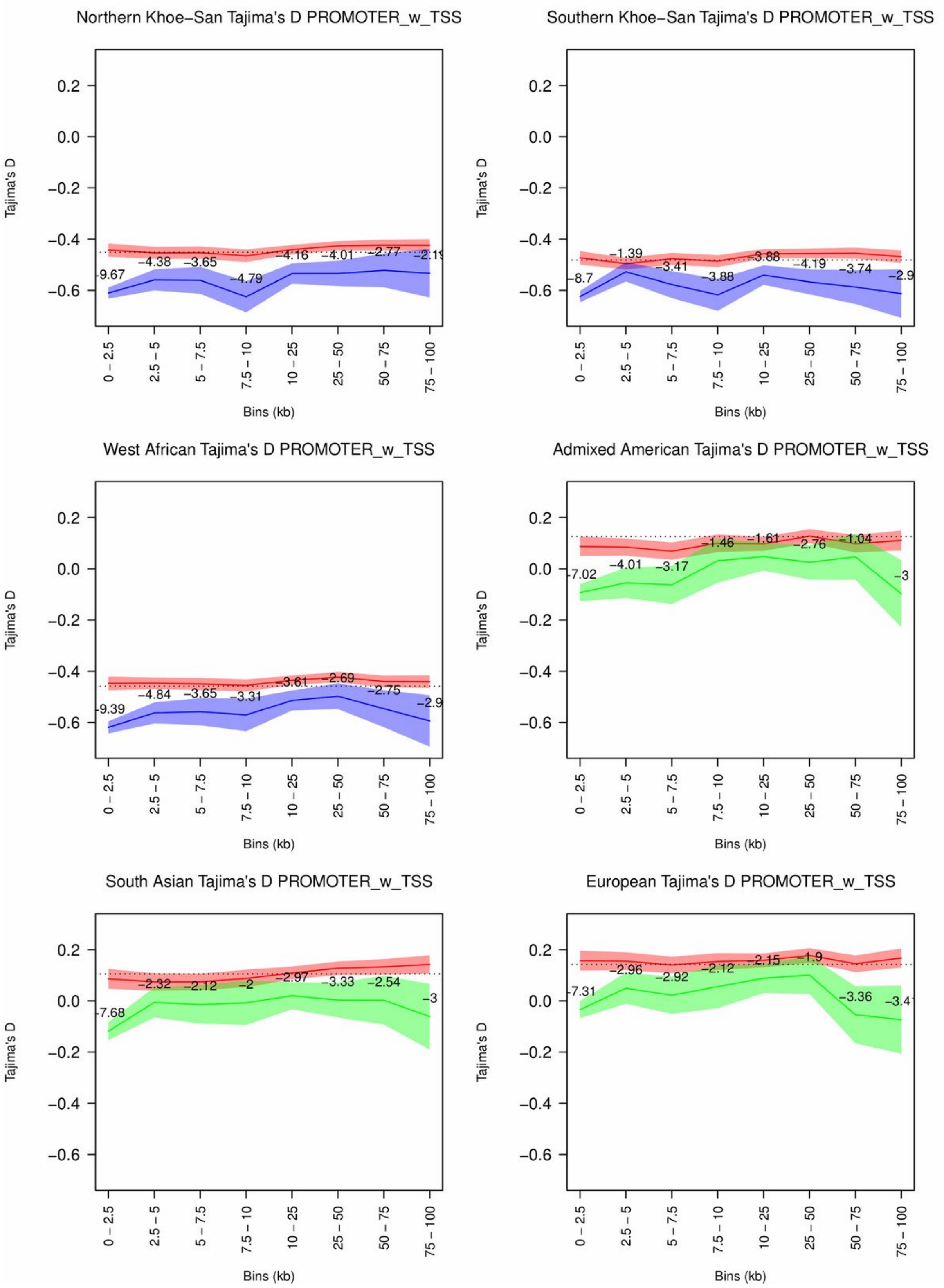

Figure S33: Tajima’s D at varying distance from CDS for non-annotated sequence (red) versus promoters, in the six global pools. Neutral reference is illustrated by dotted line. Shaded areas represent 95% confidence intervals, with Z-scores (non-annotated vs. annotation) shown per bin.

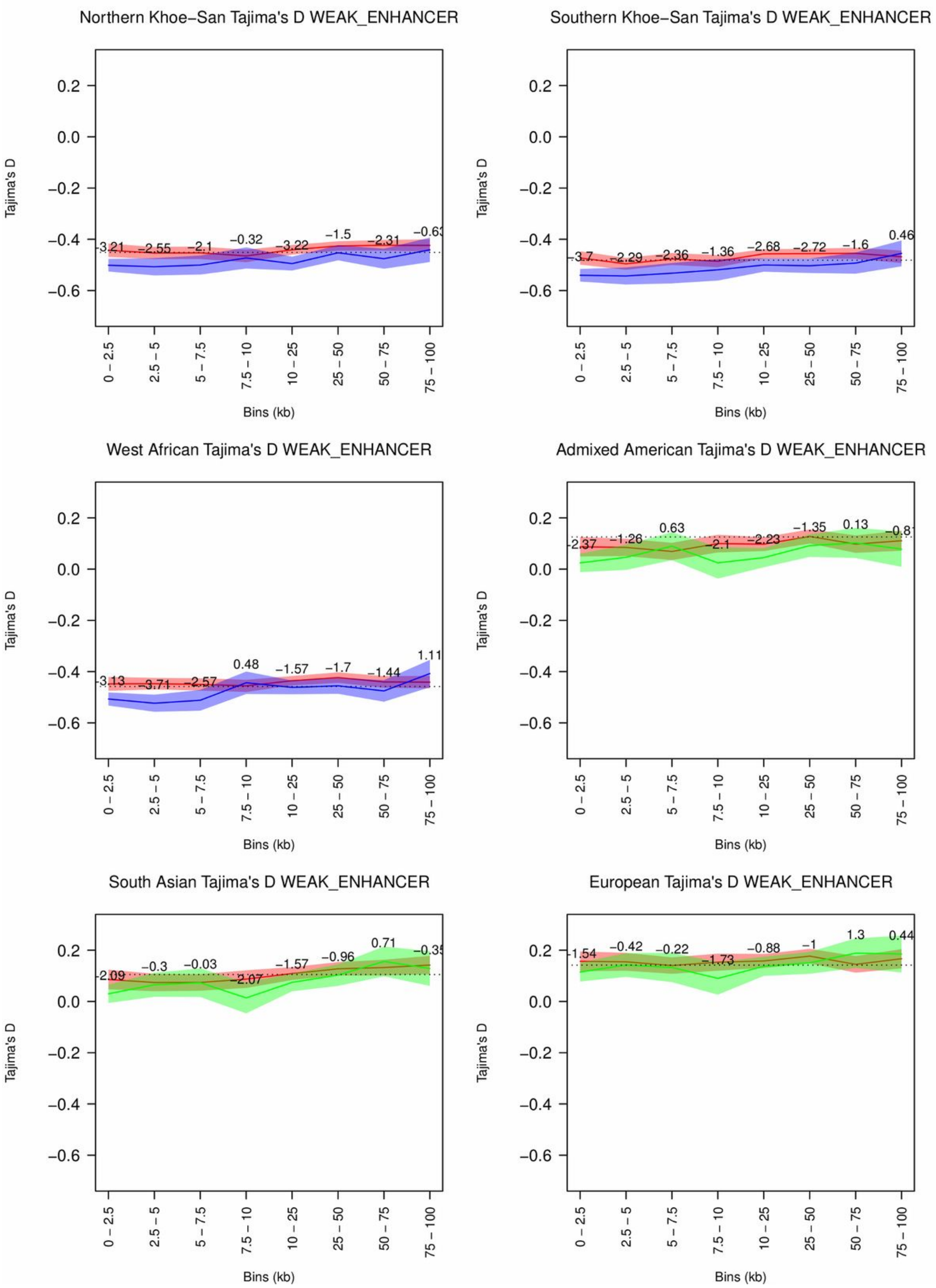

Figure S34: Tajima's D at varying distance from CDS for non-annotated sequence (red) versus weak enhancers, in the six global pools. Neutral reference is illustrated by dotted line. Shaded areas represent 95% confidence intervals, with Z-scores (non-annotated vs. annotation) shown per bin.

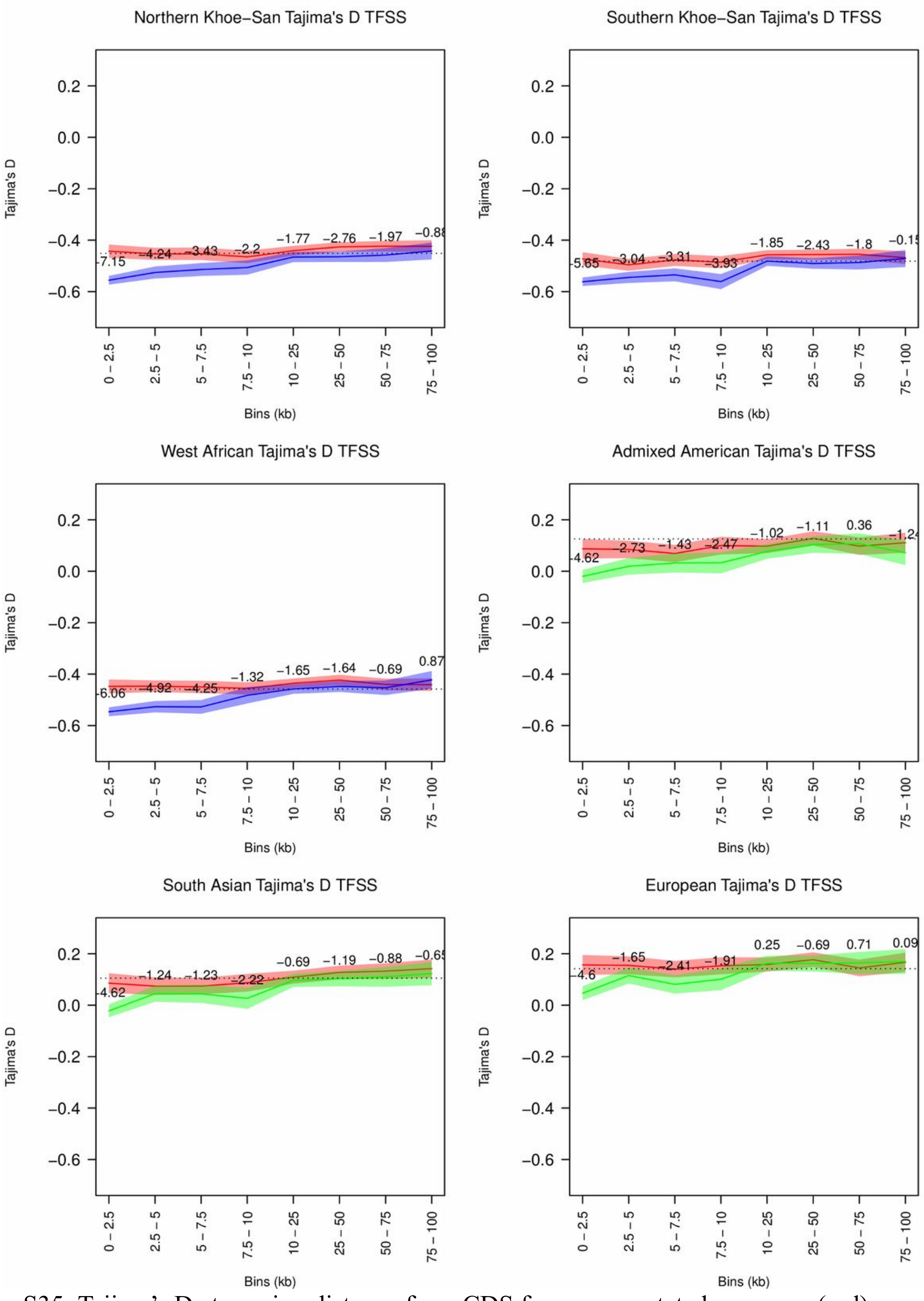

Figure S35: Tajima’s D at varying distance from CDS for non-annotated sequence (red) versus sequence-specific TFs, in the six global pools. Neutral reference is illustrated by dotted line. Shaded areas represent 95% confidence intervals, with Z-scores (non-annotated vs. annotation) shown per bin.

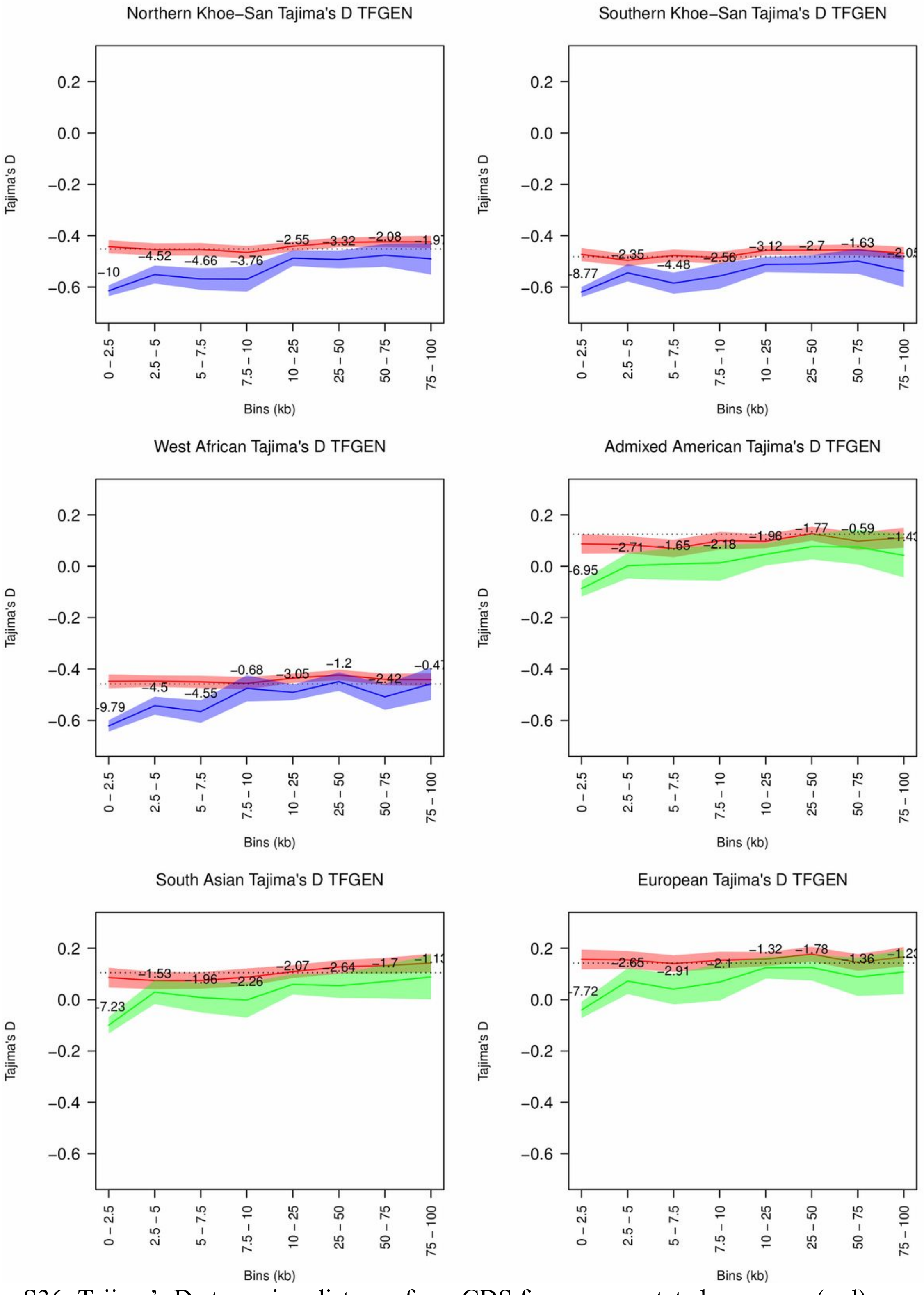

Figure S36: Tajima’s D at varying distance from CDS for non-annotated sequence (red) versus general TFs, in the six global pools. Neutral reference is illustrated by dotted line. Shaded areas represent 95% confidence intervals, with Z-scores (non-annotated vs. annotation) shown per bin.

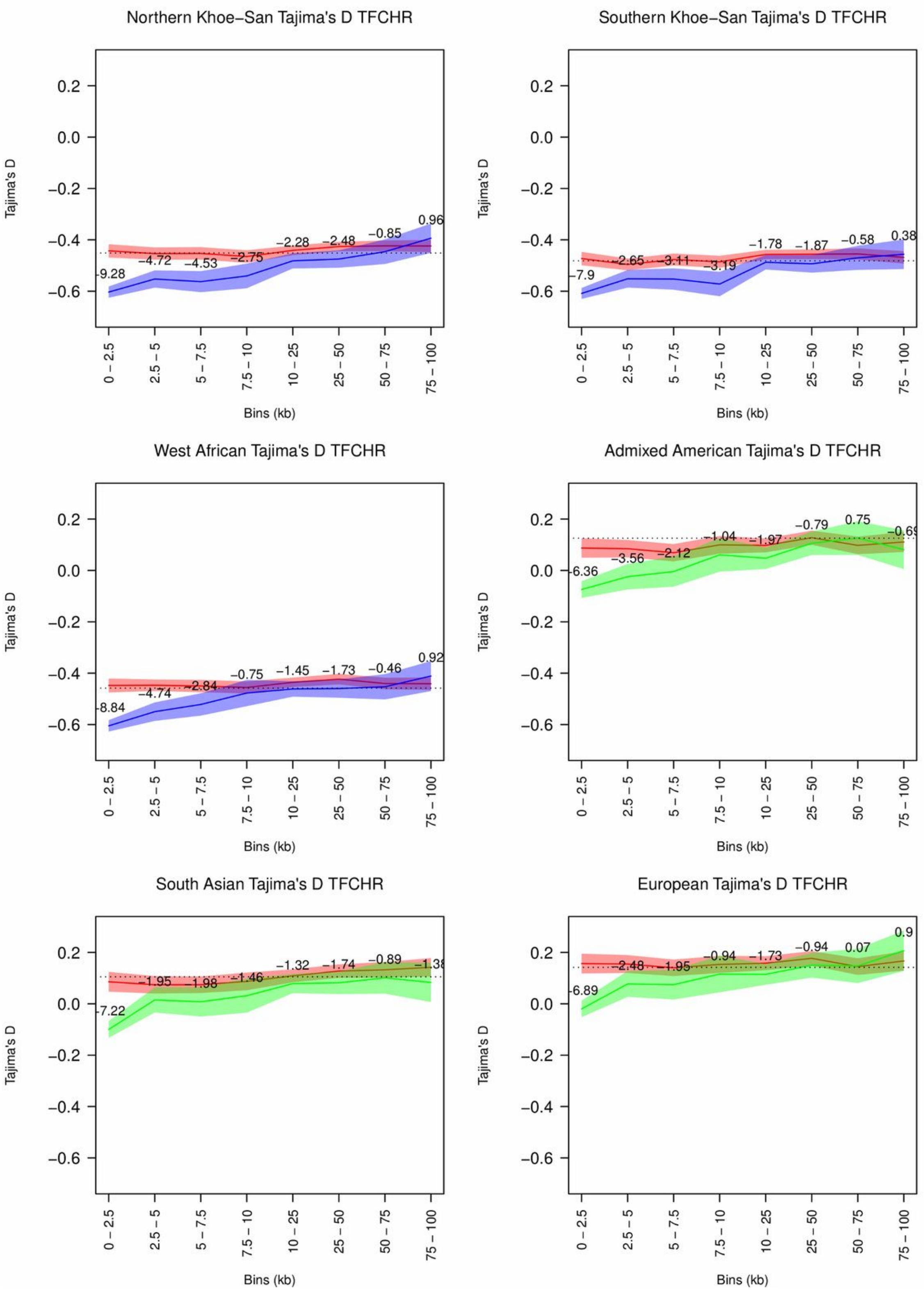

Figure S37: Tajima's D at varying distance from CDS for non-annotated sequence (red) versus chromatin-modifying TFs, in the six global pools. Neutral reference is illustrated by dotted line. Shaded areas represent 95% confidence intervals, with Z-scores (non-annotated vs. annotation) shown per bin.

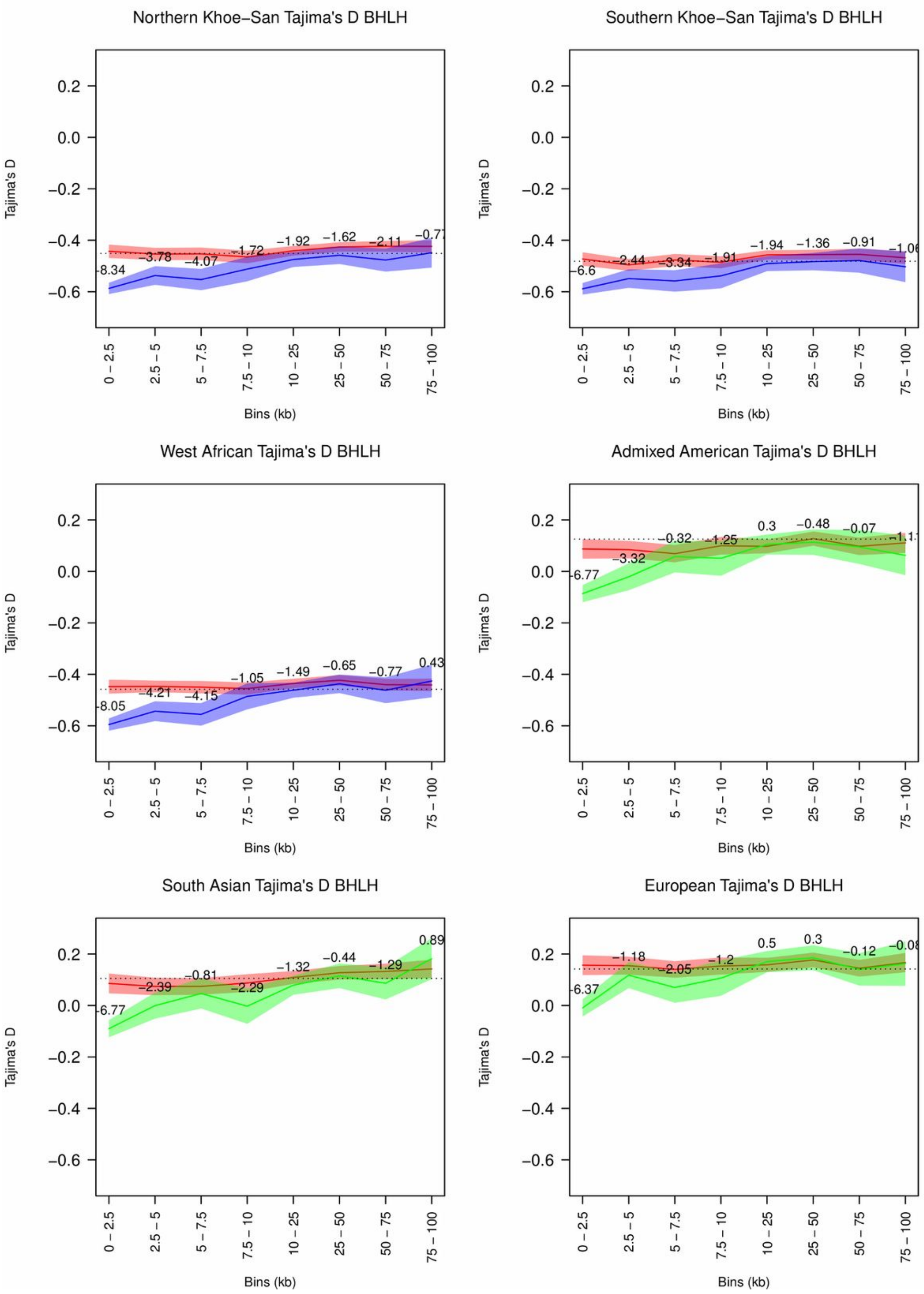

Figure S38: Tajima's D at varying distance from CDS for non-annotated sequence (red) versus bHLH family TFs, in the six global pools. Neutral reference is illustrated by dotted line. Shaded areas represent 95% confidence intervals, with Z-scores (non-annotated vs. annotation) shown per bin.

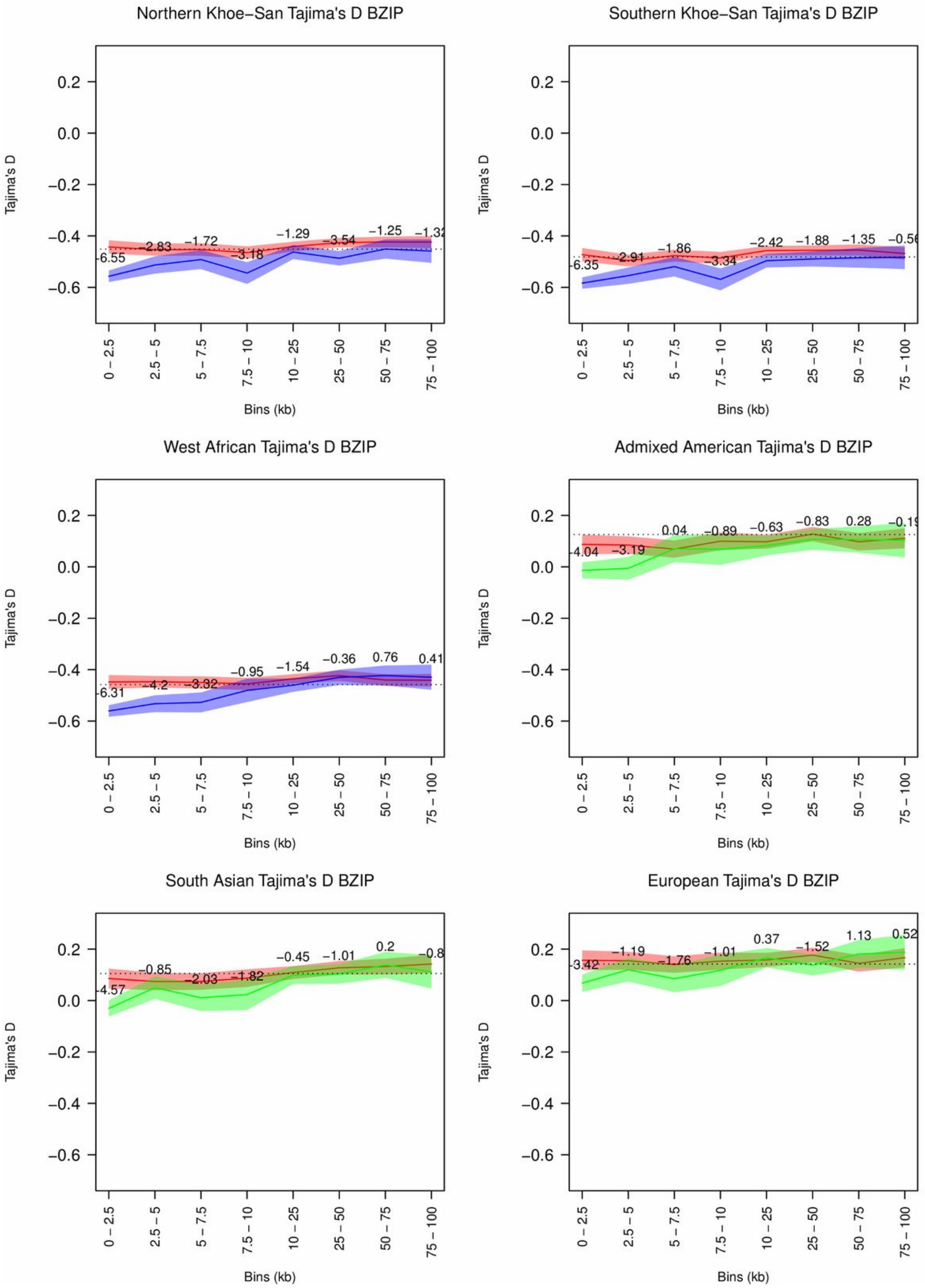

Figure S39: Tajima’s D at varying distance from CDS for non-annotated sequence (red) versus bZIP family TFs, in the six global pools. Neutral reference is illustrated by dotted line. Shaded areas represent 95% confidence intervals, with Z-scores (non-annotated vs. annotation) shown per bin.

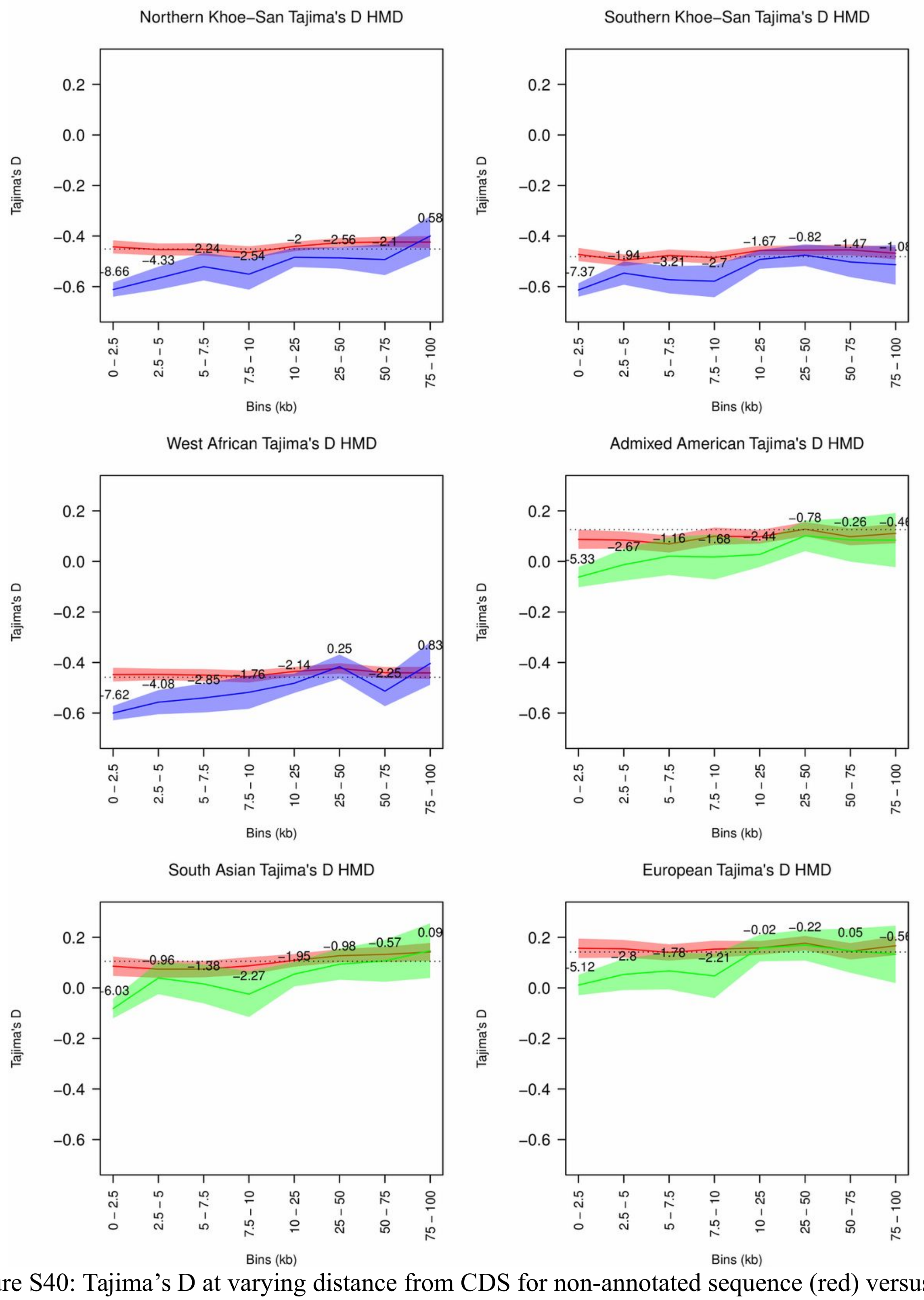

Figure S40: Tajima's D at varying distance from CDS for non-annotated sequence (red) versus HMD family TFs, in the six global pools. Neutral reference is illustrated by dotted line. Shaded areas represent 95% confidence intervals, with Z-scores (non-annotated vs. annotation) shown per bin.

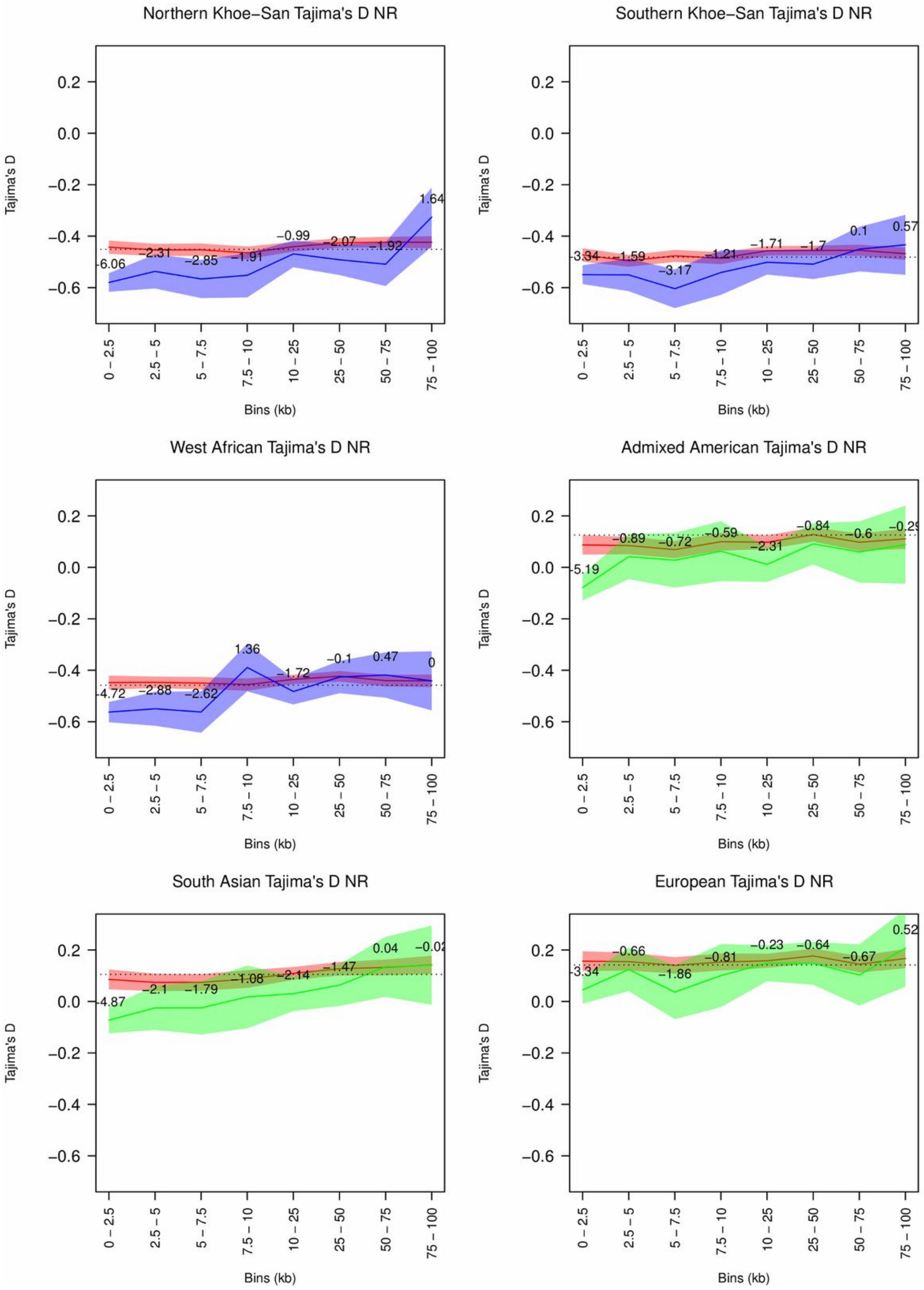

Figure S41: Tajima's D at varying distance from CDS for non-annotated sequence (red) versus NR family TFs, in the six global pools. Neutral reference is illustrated by dotted line. Shaded areas represent 95% confidence intervals, with Z-scores (non-annotated vs. annotation) shown per bin.

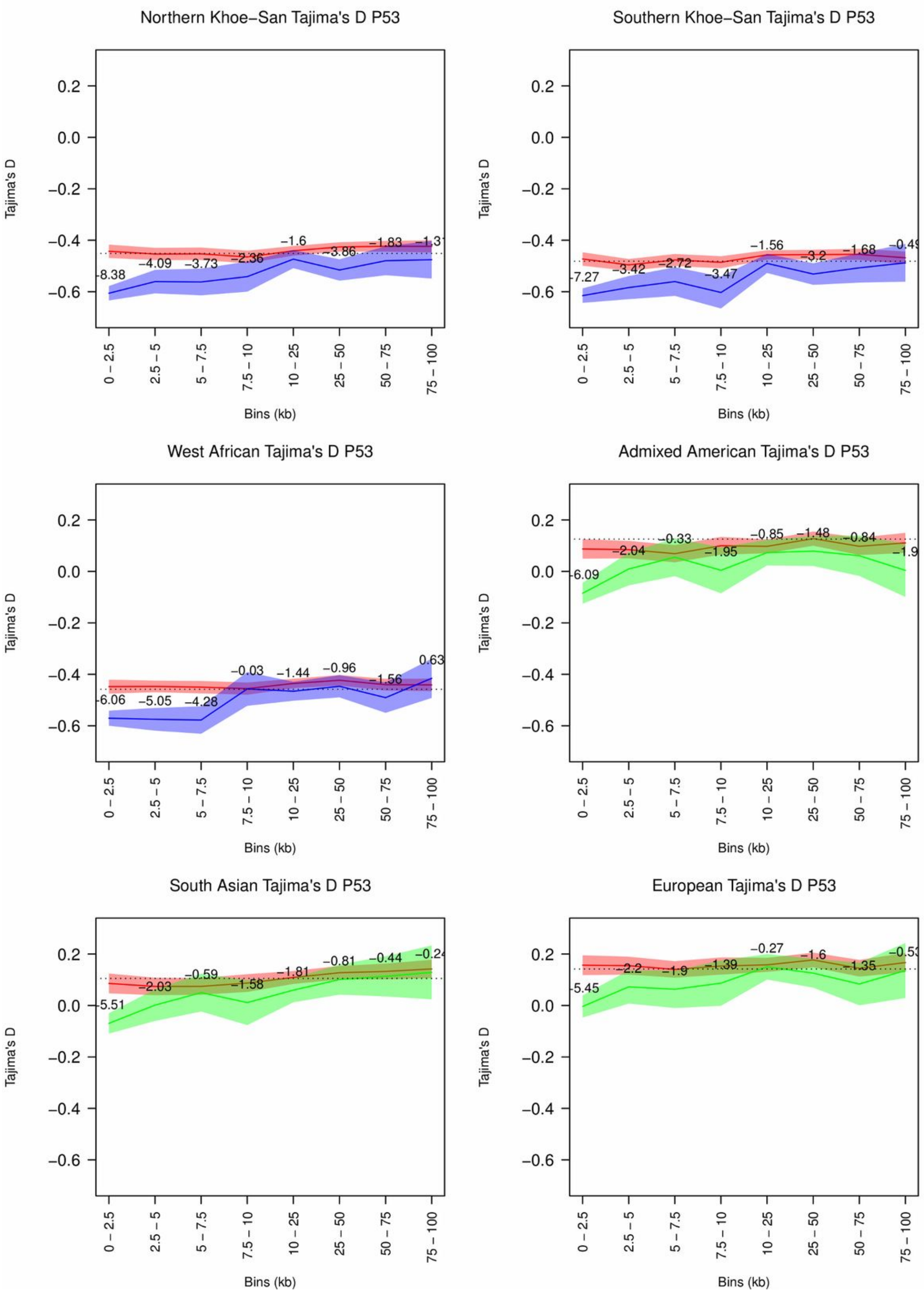

Figure S42: Tajima's D at varying distance from CDS for non-annotated sequence (red) versus P53 family TFs, in the six global pools. Neutral reference is illustrated by dotted line. Shaded areas represent 95% confidence intervals, with Z-scores (non-annotated vs. annotation) shown per bin.

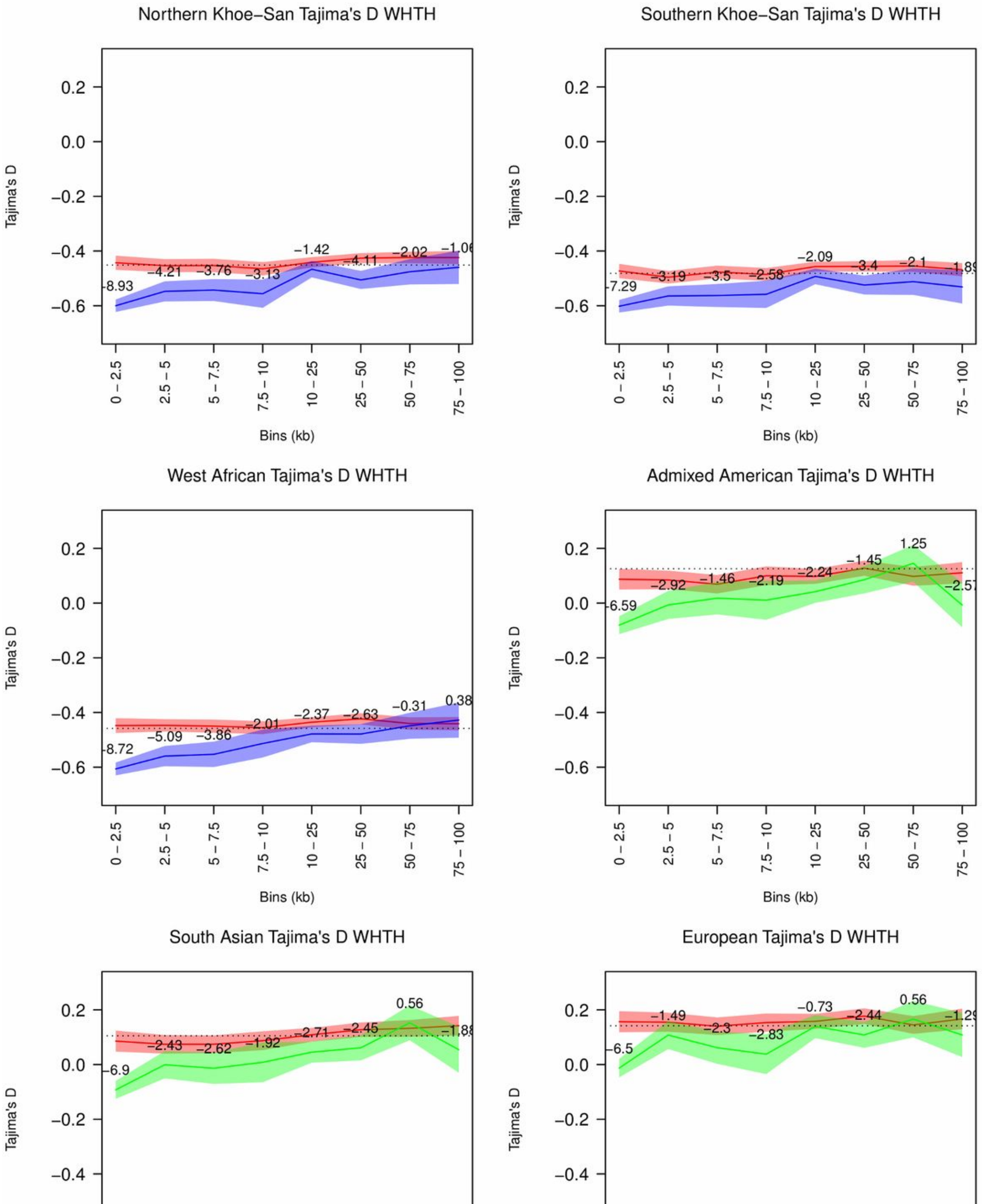

Figure S43: Tajima’s D at varying distance from CDS for non-annotated sequence (red) versus wHTH family TFs, in the six global pools. Neutral reference is illustrated by dotted line. Shaded areas represent 95% confidence intervals, with Z-scores (non-annotated vs. annotation) shown per bin.

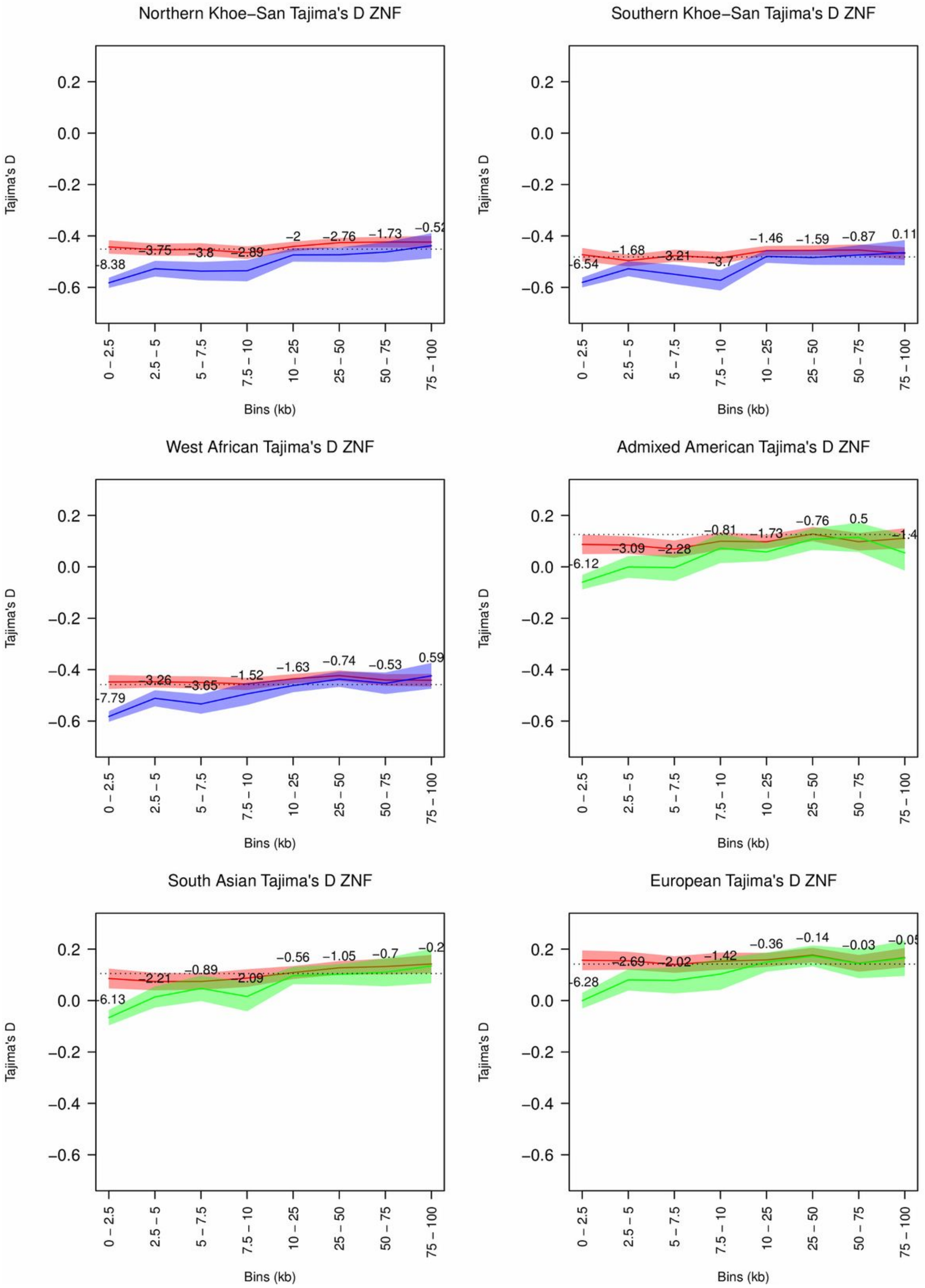

Figure S44: Tajima’s D at varying distance from CDS for non-annotated sequence (red) versus ZNF family TFs, in the six global pools. Neutral reference is illustrated by dotted line. Shaded areas represent 95% confidence intervals, with Z-scores (non-annotated vs. annotation) shown per bin.
